# Covalently constrained ‘Di-Gembodies’ enable parallel structure solutions by cryo-EM

**DOI:** 10.1038/s41589-025-01972-7

**Published:** 2025-08-15

**Authors:** Gangshun Yi, Dimitrios Mamalis, Mingda Ye, Loic Carrique, Michael Fairhead, Huanyu Li, Katharina L. Duerr, Peijun Zhang, David B. Sauer, Frank von Delft, Benjamin G. Davis, Robert J. C. Gilbert

**Affiliations:** 1 Division of Structural Biology, Centre for Human Genetics, University of Oxford, Oxford, UK; 2 Calleva Centre for Evolution and Human Science, Magdalen College, Oxford, UK; 3 Diamond Light Source, Harwell Science and Innovation Campus, Didcot, UK; 4 Department of Chemistry, University of Oxford, Oxford, UK; 5 The Rosalind Franklin Institute, Oxfordshire, UK; 6 Centre for Medicines Discovery, NDM Research Building, University of Oxford, Oxford, UK; 7 Chinese Academy of Medical Sciences Oxford Institute, University of Oxford, Oxford, UK; 8 Research Complex at Harwell, Harwell Science and Innovation Campus, Didcot, UK; 9 Department of Biochemistry, University of Johannesburg, Auckland Park, Johannesburg, South Africa; 10 Department of Pharmacology, University of Oxford, Oxford, UK; 11 Present Address: Mass Therapeutics, Ltd., Oxford, UK

**Keywords:** Protein design, Structural biology, Chemical tools, Chemical modification, Membrane proteins

## Abstract

Whilst cryo-electron microscopy(cryo-EM) has become a routine methodology in structural biology, obtaining high-resolution cryo-EM structures of small proteins (<100 kDa) and increasing overall throughput remain challenging. One approach to augment protein size and improve particle alignment involves the use of binding proteins or protein-based scaffolds. However, a given imaging scaffold or linking module may prove inadequate for structure solution and availability of such scaffolds remains limited. Here, we describe a strategy that exploits covalent dimerization of nanobodies to trap an engineered, predisposed nanobody-to-nanobody interface, giving Di-Gembodies as modular constructs created in homomeric and heteromeric forms. By exploiting side-chain-to-side-chain assembly, they can simultaneously display two copies of the same or two distinct proteins through a subunit interface that provides sufficient constraint required for cryo-EM structure determination. We validate this method with multiple soluble and membrane structural targets, down to 14 kDa, demonstrating a flexible and scalable platform for expanded protein structure determination.

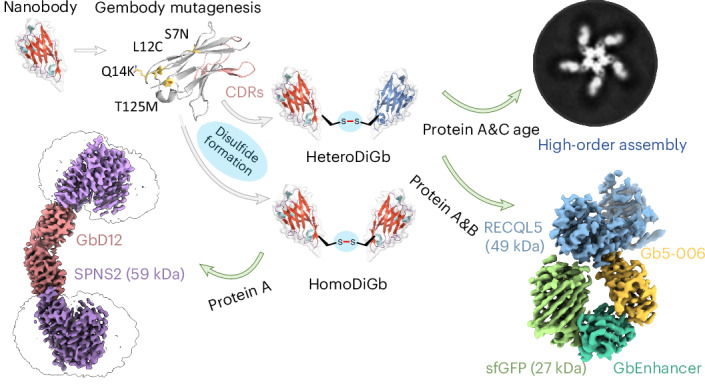

## Abstract (web summary)

Disulfide-based dimerization of modified identical and heterologous nanobody scaffolds enables higher-order assembly for high-resolution cryo-electron microscopy structure determination that is widely applicable to small protein targets.

## Main

Single-particle cryo-electron microscopy (cryo-EM) is a routine method for protein structure determination and has developed rapidly since it was first shown useful for elucidating structures of large protein complexes^1^. Nonetheless, structural determination of smaller proteins (<100 kDa) by cryo-EM remains challenging^2^. Consequently, <3.5% of deposited structures in the EM Data Bank (EMDB) have a molecular weight below 100 kDa (ref. ^3^), despite the fact that small proteins are abundant in nature, with 92.3% of protein-coding genes in humans generating products below 100 kDa and 74.5% generating products below 50 kDa (ref. ^4^). Therefore, to determine protein structures not accessible by other methods or in conditions often closer to physiological^5^, new tools and methods are needed to facilitate cryo-EM of small targets.

A major challenge for small-protein cryo-EM is the low signal-to-noise ratio of the particles^2^. This leads to difficulty in particle picking and alignment during data processing, with these inaccuracies ultimately resulting in high *B* factors and lower resolutions for three-dimensional (3D) reconstructions^2^. Therefore, cryo-EM reconstruction generally remains easier for larger targets and complexes, as they provide sufficient signal for particle alignment. Taking advantage of this, methods for increasing particle size and symmetry with fiducial markers have been developed^4^. Three categories of toolsets are currently available: high-symmetry scaffolding particles^6^ and fusions with^4,7^ or binding by additional protein structures^8–10^. Such binding modules include antibody fragments (Fab), nanobodies (Nb) and synthetic backbones with evolved or selected binding sequences. There are well documented and widely shared methods for binding module generation^11–15^ and they have become established tools in crystallographic and cryo-EM structure determination^4,16^; these are, in particular, increasingly supported for nanobodies^14,15^ but their small size can limit their standalone utility for mass enhancement. These modules can further be fused or bound to other proteins for still greater fiducial mass, including the NabFab^17^, megabody^18^, Legobody^19^ and BRIL-based^14^ technologies (Supplementary Fig. 1). BRIL-based methods, in particular, coupled with the engineering of rigidly bound epitopes into the target sequence, enables more generic Fab and nanobody targeting to multiple proteins^18,20^, thereby partially avoiding the sometimes time-consuming processes of binder generation and selection.

**Extended Data Fig. 1:**
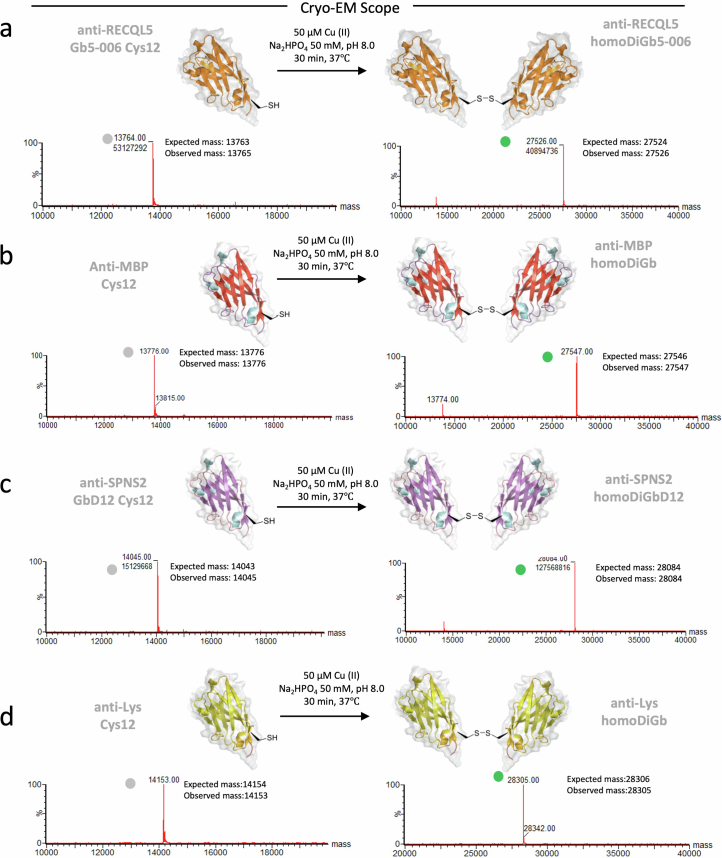
Modular generation of homo Di-Gembodies (homoDiGbs) for cryo-EM. (**a**) Gb5-006 (**b**) GbMBP (**c**) GbD12 (**d**) GbLysozyme. Intact protein mass spectra of Gembody monomers and homoDiGb with peaks indicated by circles in grey and green, respectively.

**Extended Data Fig. 2:**
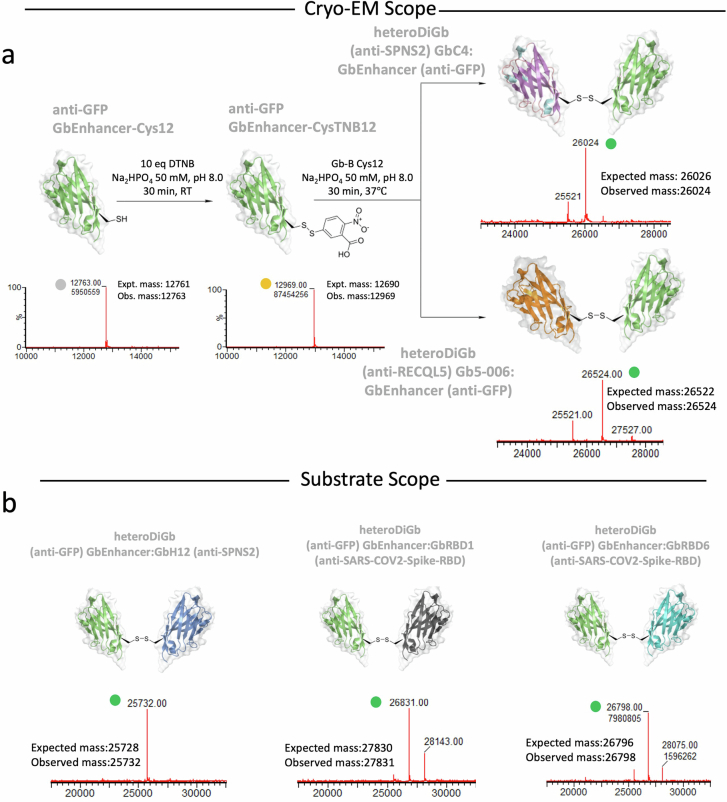
Modular generation of hetero Di-Gembodies (heteroDiGbs). (**a**) Representative generation process for heteroDiGbs used in the cryo-EM studies GbC4:GbEnhancer and Gb5-006:GbEnhancer (**b**) demonstrating the modularity. Intact protein mass spectra peaks for Gembody monomers, Gembody-TNB conjugates and heteroDiGbs are indicated by circles in grey, yellow and green, respectively.

**Extended Data Fig. 3:**
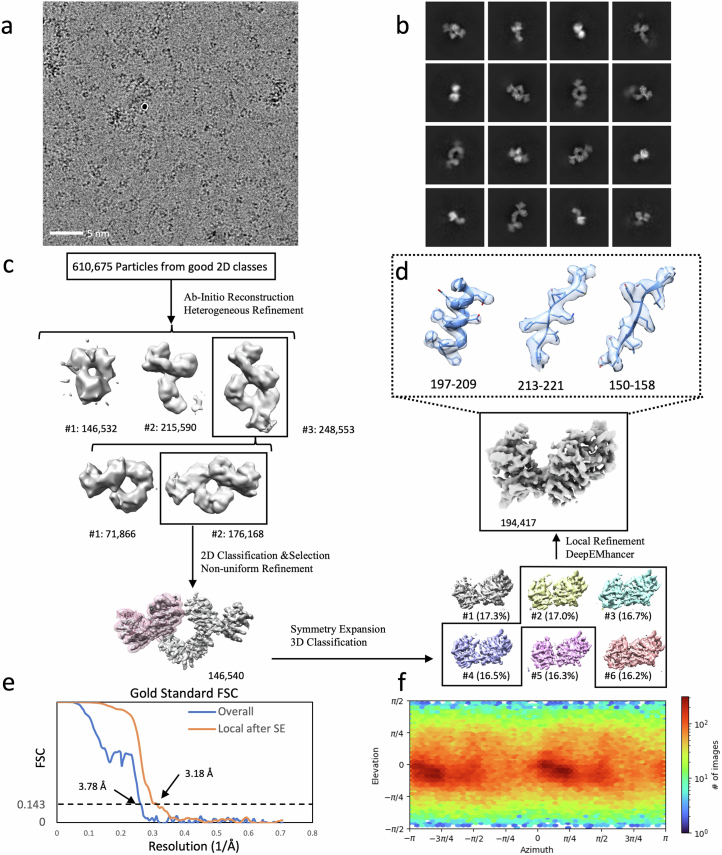
Cryo-EM structural determination of RECQL5 in complex with a homoDiGb5-006. (**a**) Exemplar raw micrographs. Similar images were captured on three separate occasions. (**b**) 2D classification results of initial particles after Topaz picking. (**c**) Cryo-EM 3D reconstruction pipeline. (**d**) Map details of RECQL5’s residues 197-209, 213-221, and 150-158, containing helical, beta sheet, and loop secondary structures, respectively. (**e**) The Fourier Shell Correlation curves of the overall complex and locally refined RECQL5 after symmetry expansion (SE). (**f**) Particle orientation distribution of the final locally refined map.

**Extended Data Fig. 4:**
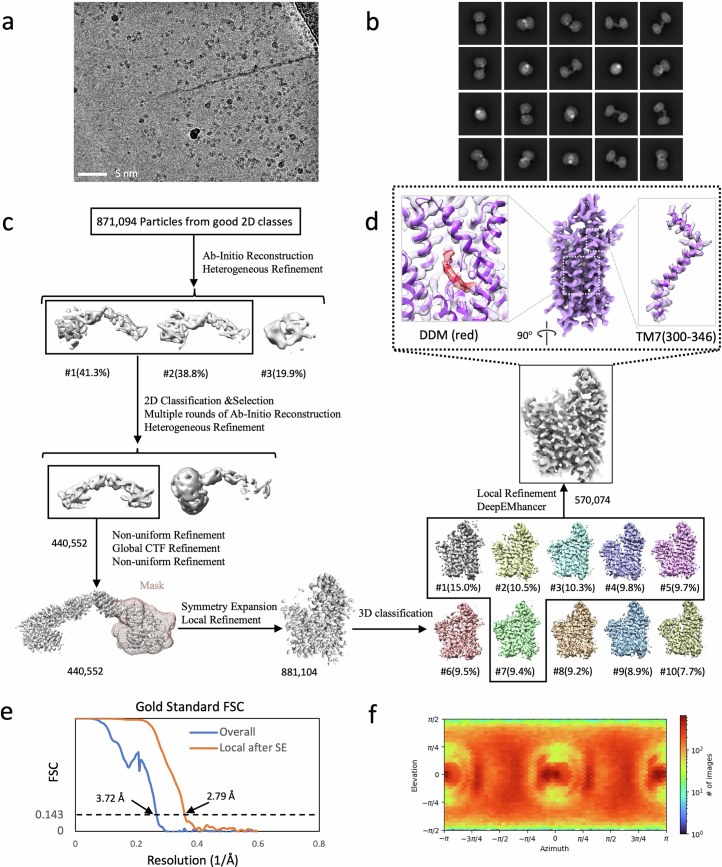
Cryo-EM structural determination of SPNS2 in complex with homoDiGbD12. (**a**) Exemplar raw micrograph. Similar images were captured on three separate occasions. (**b**) 2D classification of Topaz-picked particles. (**c**) Cryo-EM 3D reconstruction pipeline. (**d**) Cryo-EM map of SPNS2 monomer after symmetry expansion, with map-and-model overlays for TM7 residues 300-346 and central binding site bound with DDM. (**e**) The Fourier Shell Correlation curves of the overall dimer complex and locally refined SPNS2 after symmetry expansion (SE). (**f**) Particle orientation distribution of the final locally refined map.

**Extended Data Fig. 5:**
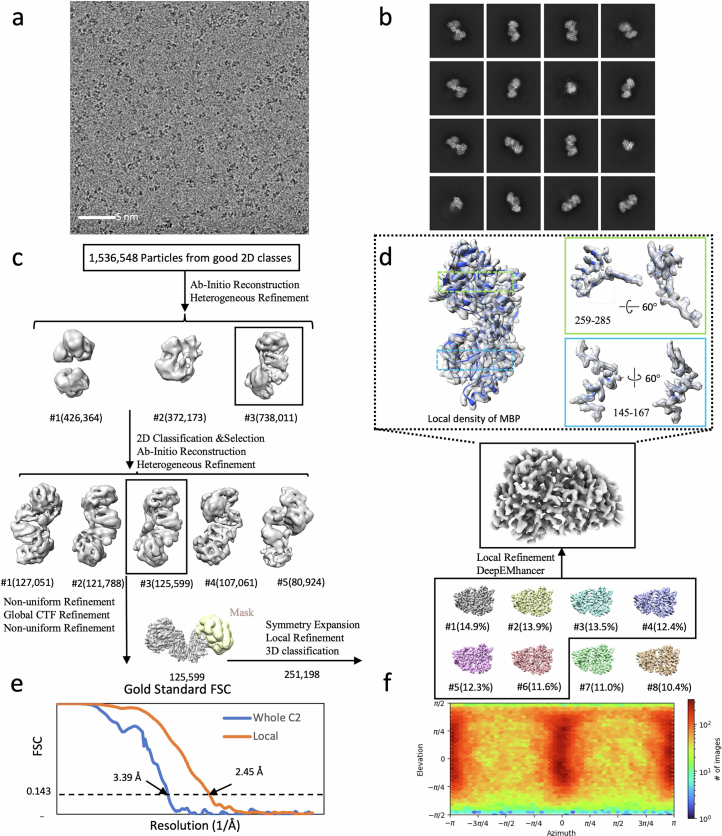
Cryo-EM structural determination of MBP in complex with homo Di-Gembody homoDiGbMBP. (**a**) Exemplar raw micrograph. Similar images were captured on three separate occasions. (**b**) 2D classification of Topaz-picked particles. (**c**) Cryo-EM 3D reconstruction pipeline. (**d**) Cryo EM map of MBP monomer after symmetry expansion, with map-and-model overlays for residues 145-167 and 259-285. (**e**) The Fourier Shell Correlation curves of the overall dimer complex and locally refined MBP after symmetry expansion (SE). (**f**) Particle orientation distribution of the final locally refined map.

**Extended Data Fig. 6:**
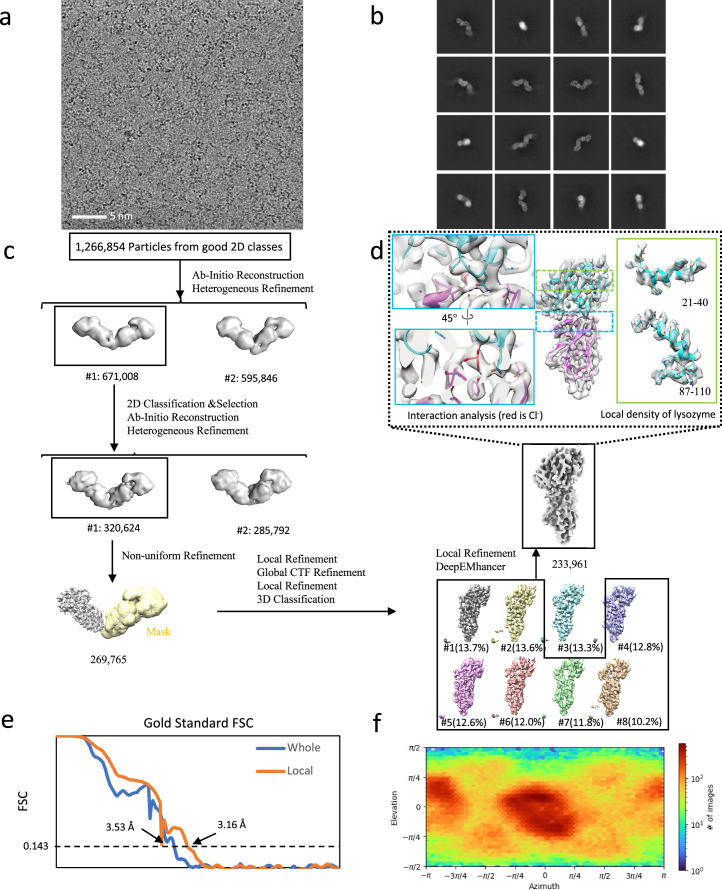
Cryo-EM structural determination of lysosome in complex with homo Di-Gembody homoDiGbLysozyme. (**a**) Exemplar raw micrograph. Similar images were captured on three separate occasions. (**b**) 2D classification of Topaz-picked particles. (**c**) Cryo-EM 3D reconstruction pipeline. (**d**) Cryo-EM map of lysozyme monomer after symmetry expansion, with map-and-model overlays for residues 21-40 and 87-110 and the binding site with the bound chloride. (**e**) The Fourier Shell Correlation curves of the overall dimer complex and locally refined SPNS2 after lysozyme. (**f**) Particle orientation distribution of the final locally refined map.

**Extended Data Fig. 7:**
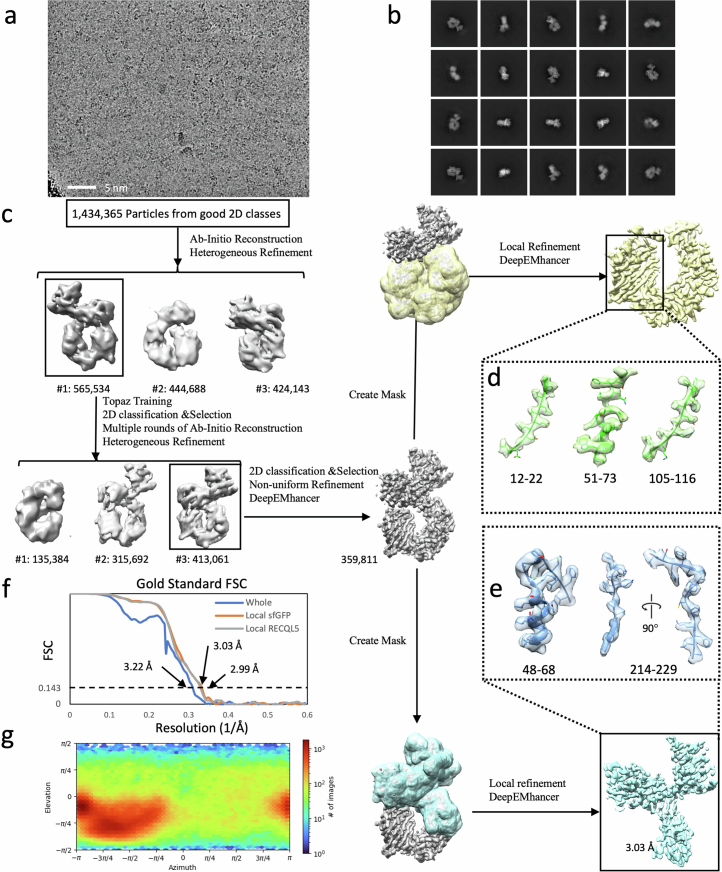
Cryo-EM structural determination of the RECQL5:heteroDiGb:sfGFP complex. (**a**) Exemplar raw micrograph. Similar images were captured on three separate occasions. (**b**) 2D classification of particles selected by Topaz picking. (**c**) Cryo-EM 3D reconstruction pipeline. (**d**) Overall view of sfGFP after local refinement, and structural details of peripheral regions of the central helix (51-73) and two beta sheets (12-22 and 105-116). (**e**) Map details of RECQL5 sub regions of the helix (48-68) and the region containing a beta sheet and a loop (214-229). (**f**) The Fourier Shell Correlation curves of the overall complex, the locally refined map of sfGFP, and the locally refined map of RECQL5. (**g**) Particle orientation distribution of the overall complex before local refinement of individual targets.

**Extended Data Fig. 8:**
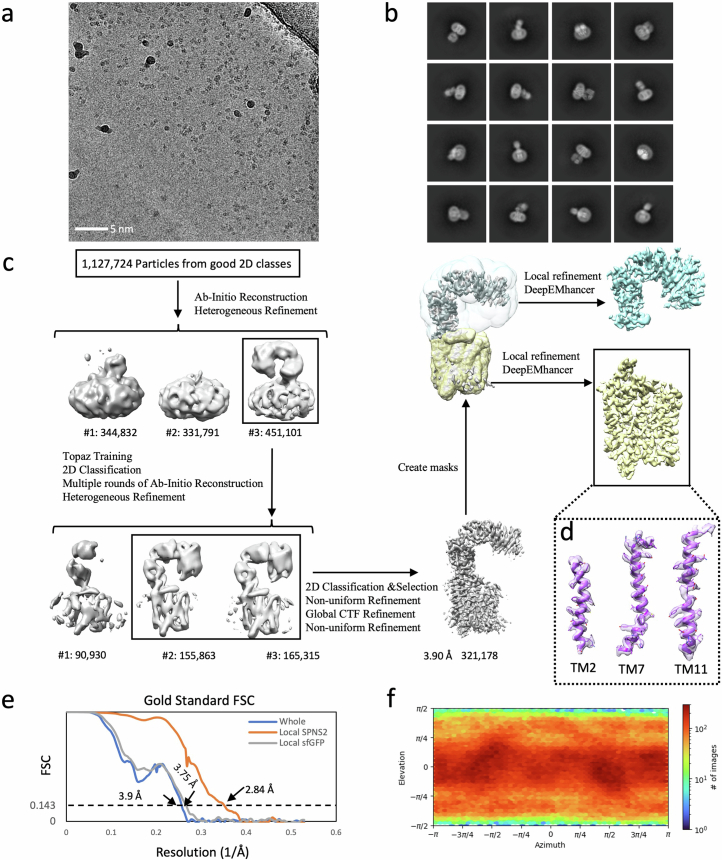
Cryo-EM structural determination of the SPNS2:heteroDiGb:sfGFP complex. (**a**) Exemplar raw micrograph. Similar images were captured on three separate occasions. (**b**) 2D classification of particles selected by Topaz picking. (**c**) Cryo-EM 3D reconstruction pipeline. (**d**) Map and model of SPNS2’s transmembrane helices of TM2, TM7 and TM11. (**e**) The Fourier Shell Correlation curves of the overall complex before local refinement, the locally refined map of SPNS2, and the locally refined map of sfGFP. (**f**) Particle orientation distribution of the dimer complex before local refinement.

Despite this demonstrated proof of principle, challenges with binder:target complex assembly still present problems for such fiducial-assisted single-particle cryo-EM. Fusing tags or inserting epitopes requires modifying the target itself and thereby risks altering the protein’s native structure, potentially also reducing its expression or requiring extensive screening of constructs^21^. In addition, most tools do not have symmetry, requiring chimeric constructs and, thus, needing subcloning and expression optimization^6,17,19^. Above all, these existing scaffolds are monospecific and, consequently, do not address generality or modularity; there is an urgent need for greater sample throughput (Supplementary Fig. 1). Therefore, there remains a need to develop complementary strategies for fiducial optimization, ideally with the potential to multiplex structure determination. Here, we show that a generic and kinetically biased ‘Gembody’ (Gb) interface, previously discovered through trapping in crystallo^22^, can also be trapped in solution by kinetically controlled side-chain-to-side-chain bond formation in a manner that leads to ready generation of complexes; these display ideally balanced interface flexibility and yet relative complex constraints, in turn allowing rapid cryo-EM structure determination, even of small targets. This approach was effectively used to assemble macromolecular complexes (Extended Data Figs. 1 and 2 and Supplementary Figs. 2–4), culminating in the determination of six cryo-EM structures at resolutions spanning 2.45 Å to 3.75 Å (Extended Data Figs. 3–8).

## Results

### HomoDiGbs aid small-protein structure determination

One ideal ‘plug-and-play’ design for this needed tool would be through the constrained complexation of two binding modules through a structurally robust interface away from their binding loops, in which case, modularity, symmetry and bispecificity are all achieved. Analysis of our previous crystallographic observations of protein modules, based on nanobodies that self-assemble or ‘twin’ in the solid phase, revealed a surprising and seemingly generic noncovalent in crystallo interface that we reasoned could be rendered stable through designed covalency. In this way, kinetically controlled trapping in crystallo might parallel and, thus, inform efficient kinetically controlled trapping in solution.

**Fig. 1:**
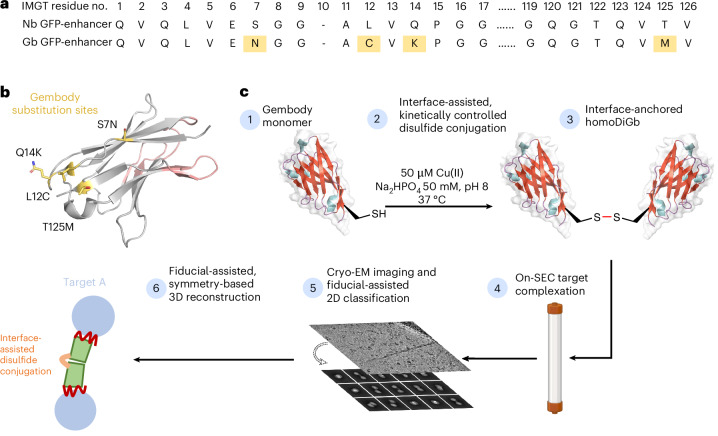
Application of homoDiGbs enables high-resolution cryo-EM structure determination for dual copies of small proteins. **a**, Designed Gb core substitutions (S7N;L12C;Q14K;T125M in gold) on the nanobody backbone enable chemically driven, covalent DiGb generation. The sequences are aligned using the IMGT scheme developed for immunoglobulin folds^35^. The sequence difference between anti-GFP nanobody NbEnhancer (PDB 3K1K)^27^ and its Gb equivalent (Supplementary Fig. 5) is shown as an example. **b**, Schematic cartoon of the anti-GFP nanobody NbEnhancer showing the Gb substitution sites (gold) away from CDRs (pink). **c**, Construction pipeline for homoDiGb. BioRender.com license for the SEC column item: XM289VST7B.

In principle, many covalent bond-forming (‘conjugation’) methods are available to link protein modules. However, few allow the generation of a minimally sized link. Furthermore, this method must balance the needed proximity for our designed interfacial constraint and yet a sufficiently efficient reaction under the inherently challenging second-order kinetics of protein dimerization at low concentrations. The observed critical role of pivotal residues in driving ‘twinning sites’ at protein–protein interfaces in the solid phase^22^ led us to test these as promising sites for such chemistry. Ultimately, after a survey of in-solution covalent bond-forming methods exploring differing natural and unnatural amino acid residues, we found that Cu(II)-catalyzed oxidation at a pivotal C12 residue site (together with the presence of needed Gb ‘gem’ substitutions previously discovered through iterative X-ray-guided engineering^22^) offered a route to direct and rapid, high-yielding formation of disulfide-linked homo Di-Gembody (homoDiGb) without side reactions (for example, off-site oxidation) (Fig. 1a–c, Extended Data Fig. 1 and Supplementary Figs. 5 and 6a) to form a robust covalent interface.

Specifically, a balance of flexibility and constraint of the DiGb interface was key to the chemical reaction and complexation required for cryo-EM. We reasoned that longer linkers generated by many current conjugation methods would engender too much flexibility; hence, minimally sized covalent side-chain-to-side-chain motifs were chosen (four or five bonds in length from Cα to Cα). Whilst lanthionine-based thioether (four-bond) conjugation was initially considered (as a contracted bond analog of cysteine-based, five-bond linker) and tested, this was previously only synthetically induced in proteins in an intramolecular manner^23^; the distances across and the nature of the DiGb interface apparently did not allow its intermolecular use here.

**Fig. 2:**
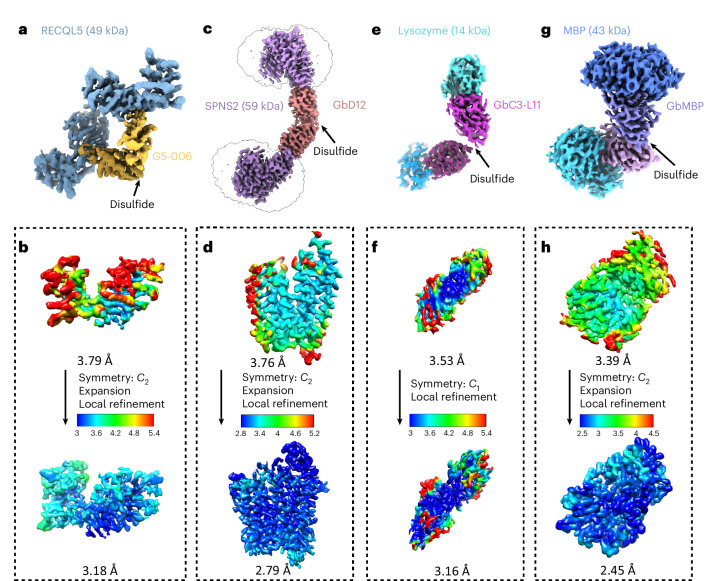
Application of homoDiGbs enables high-resolution cryo-EM structure determination for dual copies of small proteins. **a**–**g**, High-resolution cryo-EM reconstruction maps of RECQL5 (**a**), SPNS2 (**c**), lysozyme (**e**) and MBP (**g**) in complex with their respective homoDiGbs. Local resolution comparisons before and after local refinement with or without *C*_*2*_ symmetry expansion for RECQL5 (**b**), SPNS2 (**d**), lysozyme (**f**) and MBP (**h**).

To probe the applicability and value of these homoDiGbs to cryo-EM structure determination, we tested the 49-kDa DNA helicase RECQL5 as a challenging soluble target with both small size and substantial interdomain flexibility^24^. As an initial experiment, we compared the two-dimensional (2D) cryo-EM classes of RECQL5 alone, RECQL5 bound to a wild-type nanobody and RECQL5 in complex with the corresponding homoDiGb (homoDiGb5-006) generated from our chemically driven, covalent dimerization workflow (Fig. 1c and Supplementary Fig. 6a). With the same particle-picking strategies, homoDiGb resulted in many well-resolved classes for different views (Supplementary Fig. 7), appreciably outperforming the other RECQL5 samples. Next, a larger dataset of this RECQL5:homoDiGb complex on a 300-kV microscope with standard operating parameters (Supplementary Table 1) yielded a 3.79-Å reconstruction of the complex after imposing *C*_2_ symmetry. Because of minor flexibility within the DiGb interface, imposition of strict *C*_2_ symmetry prevents refinement to high-resolution signals. Therefore, we applied an approximate ‘nonstrict *C*_2_ symmetry’ that improved the alignment of the particle images (Methods). In this way, this initial map could be further improved by forgoing strict *C*_2_ symmetry, with symmetry expansion and local refinement of RECQL5 alone improving the resolution to 3.18 Å (Fig. 2a,b and Extended Data Fig. 3). While RECQL5:Gb5-006 could be resolved using the same pipeline without dimerization, the resolution and map quality were substantially worse (Supplementary Figs. 8a and 9). Similarly, the clinically-relevant, membrane protein Sphingosine-1-phosphate (S1P) transporter Spinster Homolog 2 (SPNS2) was resolved to 2.79 Å locally, when bound to the equivalent homoDiGbD12 derived from the SPNS2-binding nanobody NbD12 (ref. ^10^) (Fig. 2c,d and Extended Data Fig. 4). Notably, the homoDiGb-bound structure of SPNS2 was found in the same inward-facing *n*-dodecyl-β-d-maltoside (DDM)-bound state as when bound to wild-type nanobody NbD12, indicating minimal perturbation to the structure. The comparison showed clear improvements over the previously published structures of SPNS2 bound to NbD12 (EMD-18668)^10^ (with better-defined side chains, Supplementary Fig. 10a) and, importantly, over structures determined with NabFab (EMD-34104) or DARPin and maltose-binding protein (MBP) fusions (EMD-28650)^3,7^. This increased resolution usefully revealed interactions at the intracellular loops of SPNS2, including the important PtdIns(4,5)P_2_ pocket (site 2; Supplementary Fig. 10a)^25^. The complex of RECQL5 with an isolated (nondimerized) Gb shows orientational bias, whereas neither RECQL5 or SPNS2 structures with DiGb are noticeably affected by it (Extended Data Fig. 3f and Supplementary Fig. 9e,f). Thus, an additional benefit of the DiGb method may be through introducing more orientations to reduce the bias on a case-by-case basis, as well as the fundamental increases that we observe in alignment efficiency that arise from more views, driven by introducing characteristic DiGb modular shapes.

The rapid implementation of the homoDiGb method and the additional insights gained into target structure function led us to consider the limits of this technology. Current cryo-EM structure determination methods typically struggle with targets below 50 kDa, contrasting with our ready solution of the structure of 49 kDa RECQL5. We, therefore, chose to probe even smaller targets. Strikingly, within weeks, using an essentially identical workflow to that used for RECQ5 and SPNS2, the homoDiGb method was applied to 43-kDa MBP and even very small 14-kDa hen egg white lysozyme (Extended Data Fig. 1 and Supplementary Fig. 3), achieving reconstructions at 2.45 Å and 3.16 Å, respectively (Fig. 2e–h and Extended Data Figs. 5 and 6). Interestingly, for lysozyme, an archetype of X-ray structure determination that we solve here by cryo-EM single-particle analysis (SPA) for the first time, we found that *C*_1_ symmetry reconstruction followed by local refinement yielded better resolution than imposing *C*_2_ symmetry for the entire particle. This putative observation of nominal asymmetry for such extremely small proteins may offer strategic solutions for other very small targets as this field grows. Together, these data demonstrated that the homoDiGb approach provides a rapid, generic and efficient way for cryo-EM structural solution now even of small proteins.

### HeteroDiGbs enable dual resolution of two structures

**Fig. 3:**
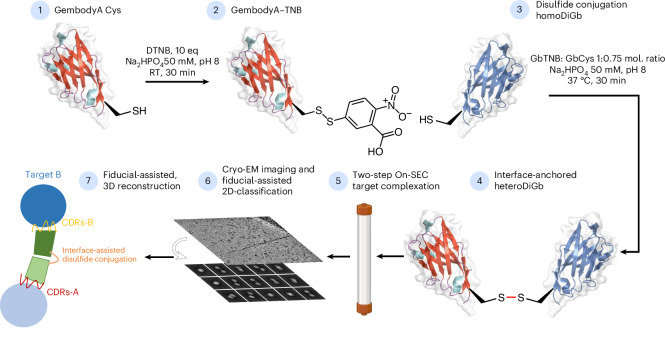
Synthesis of heteroDiGbs enables simultaneous high-resolution structure solution of two different small proteins. Construction pipeline for heteroDiGb, through a trapped intermediate. BioRender.com license for the SEC column item: XM289VST7B. RT, room temperature.

**Fig. 4:**
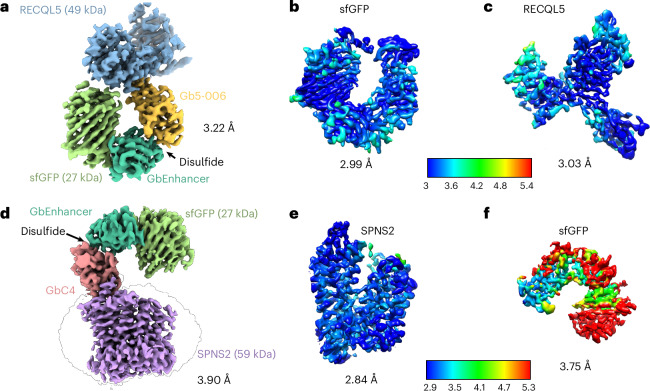
Synthesis of heteroDiGbs enables simultaneous high-resolution structure solution of two different small proteins. **a**, High-resolution cryo-EM map of RECQL5 and sfGFP in complex with heteroDiGb Gb5-006:GbEnhancer. **b**,**c**, Individual local refinement resulted in higher resolutions of sfGFP (**b**) and RECQL5 (**c**). **d**, High-resolution cryo-EM map of SPNS2 and sfGFP in complex with heteroDiGb GbC4:GbEnhancer. **e**,**f**, Individual local refinement resulted in higher resolutions of SPNS2 (**e**) and sfGFP (**f**).

Next we explored bi-specificity of this tool, by testing the extension of our modular DiGb method to the more challenging generation of heteroDiGbs. We reasoned that the needed kinetic control of covalency might be readily achieved through oxidative preactivation of a Gb followed by the addition of a second Gb to then yield a homogeneous heterodimeric population through a trapped oxidation relay. This involved the creation of an identifiable oxidized intermediate that could then be converted to the heteroDiGbs as a second oxidized product. This proved successful; specifically, quantitative functionalization at site 12 of the first binder with oxidant 5,5-dithio-bis-(2-nitrobenzoic acid) (DTNB)^26^ produced a clean, trapped intermediate that was then readily reacted with a second Gb to form the desired heteroDiGbs with high selectivities and yields (Fig. 3, Extended Data Fig. 2 and Supplementary Fig. 6b). Strikingly, using sequential purification, we generated heteroDiGbs bound to two distinct targets. Starting with a heteroDiGb of Gb5-006 and GbEnhancer, the RECQL5:heteroDiGb:sfGFP (superfolder GFP) complex structure was determined to an overall resolution of 3.22 Å (Fig. 4a and Extended Data Fig. 7). Moreover, when we locally refined each target separately, we found much-improved individual maps of 3.03 Å for RECQL5 and 2.99 Å for sfGFP (Fig. 4b,c and Extended Data Fig. 7). In an essentially identical workflow using heteroDiGb GbC4:GbEnhancer, the SPNS2:heteroDiGb:sfGFP complex structure was determined to 3.90 Å overall (Fig. 4d and Extended Data Fig. 8), with 2.84 Å for SPNS2 and 3.75 Å for sfGFP, individually (Fig. 4e,f, Extended Data Fig. 8). These results clearly demonstrated the efficiency offered by heteroDiGbs in duplexing structure determination. Further validating these methods, all of the structures obtained were largely consistent with deposited structures, with the only changes being small interdomain movements observed in RECQL5 and its slightly different Gb conformations (Supplementary Fig. 10c).

Notably the heteroDiGb strategy can also further overcome limitations of the homoDiGbs. While homoDiGbs improved resolvability and resolution for the RECQL5 and SPNS2 test cases, this strategy failed with sfGFP^27^. Alignment of a previously determined NbEnhancer:sfGFP complex to our determined homoDiGb structures suggested that the two target molecules would likely clash in the homoDiGb complex despite flexibility of the intra-DiGb angle (Supplementary Figs. 11a and 12). By contrast, heteroDiGbs constructed from NbEnhancer and the RECQL5 nanobody yielded high-resolution results.

**Extended Data Fig. 9:**
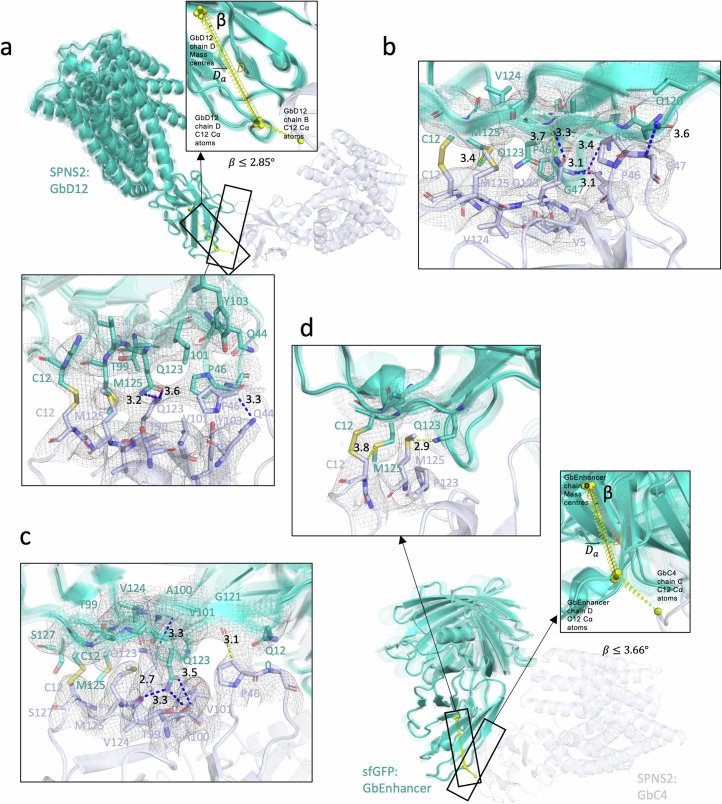
3D variability analyses of Di-Gembody structures. Sixty models of each structure are overlaid and aligned using the static Gembody. Static and moving Gembodies are shown in light blue and teal, respectively. The left insets indicate the reference points (yellow spheres), vectors ($\mathop{{D}_{i}}\limits^{ \rightharpoonup }$ shown as a yellow arrow) and angle $\beta$ for the moving Gembody used in the analyses. $\mathop{{D}_{a}}\limits^{ \rightharpoonup }$ shown as a black arrow is the average vector of $\mathop{{D}_{i}}\limits^{ \rightharpoonup }$. The right insets show the Di-Gembody interfaces with the local density shown in mesh, interacting residues shown in stick, and hydrogen bonds shown with blue dashes. Distances are shown in Å. The analyses include (**a**) SPNS2:homoDiGb (**b**) RECQL5:homoDiGb (whole map and wobbling angle shown in Fig. 3a) (**c**) RECQL5:heteroDiGb:sfGFP (whole map and wobbling angle shown in Fig. 3c) and (**d**) SPNS2:heteroDiGb:sfGFP. Source data

Summarizing all structures solved with DiGb, the five-bond, cystine-based side-chain covalency yielded varying intra-DiGb angles between 71.3° and 100.7° for different DiGb variants (Supplementary Fig. 11b and Supplementary Table 2) with distinct sets of interacting residues (Supplementary Table 2), implying an interface that may guide ‘twinning’ and yet allow sufficient flexibility for covalent reaction. Detailed 3D variability analysis (3DVA) revealed that, within a given single complex, the intra-DiGb angle varied by at most 8.1° (Extended Data Fig. 9 and Supplementary Table 2). Together, these results suggest that, once covalently linked, the DiGb interfaces of homomeric and heteromeric complexes settle into discrete local energy minima, thereby yielding a structurally homogeneous population, which potentially explains the ready application of balanced constraint (Discussion).

### Micromolar-affinity nanobodies are useful in DiGbs

**Fig. 5:**
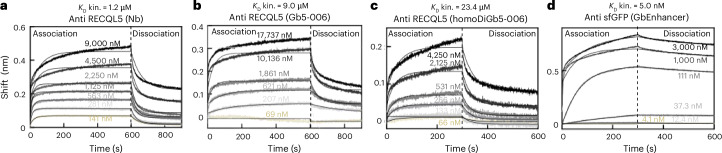
Kinetic trapping allows use of even modest underpinning affinities for protein targets. BLI measurements for RECQL5 nanobody, Gb and homoDiGb and sfGFP Gb show affinity reductions after Gb mutagenesis and generation of homoDiGbs. **a**–**d**, BLI measurement plots with fitted lines are shown in the sequence of anti-RECQL5 wild-type nanobody (**a**), anti-RECQL5 monomeric Gb5-006 (**b**), anti-RECQL5 homoDiGb5-006 (**c**) and anti-sfGFP monomeric GbEnhancer (**d**). The kinetic dissociation constants are indicated above the individual plot titles. One replicate is shown in each plot. Source data

The generation of protein complexes under kinetic control that underpins our use of homoDiGbs and heteroDiGbs raises the intriguing question of needed affinities (and associated limits) of homoDiGbs and heteroDiGbs for their targets. Interestingly, in each case, the dissociation constants measured for each were weaker than for corresponding nanobodies or indeed monovalent Gbs (Fig. 5; *K*_D_ = 1.2 μM for RECQL5 nanobody → 9.0 μM for Gb equivalent → 23.4 μM for homoDiGb; *K*_D_ = 0.5 nM (ref. ^27^) for GFP enhancer nanobody → 5 nM for its Gb). A brief survey of the literature suggests that nanobodies currently used for structural methods display *K*_D_ values in the range of 20 nM or less^28–30^. Whilst these data suggest at present that a *K*_D_ of ~20 μM or lower may be required for the Gb workflow, we cannot discount the possibility of efficacy for even weaker-binding Gb systems. We did not notice any practical effects on workflows for generating protein complexes (for example, during size-exclusion chromatography (SEC)) using weaker binders. Notably, these findings challenge a prevailing notion that tight binders are essential as fiducial markers for facilitating structure determination. They also further highlight the utility of our kinetically driven method in its accommodation, through such trapping, of complexes with even modest (micromolar) affinities.

### Side-chain covalency constrains the DiGb interface

**Fig. 6:**
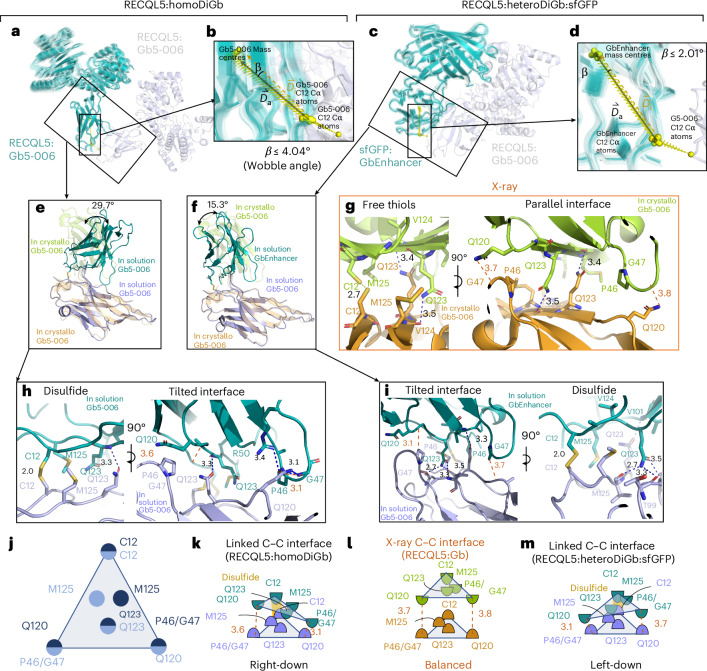
Side-chain-to-side-chain-linked DiGb interfaces are tilted and asymmetric. **a**–**d**, A 3DVA revealing the flexibility around the DiGb interfaces of RECQL5:homoDiGb (**a**) and RECQL5:sfGFP:heteroDiGb (**c**) with the respective insets (**b**,**d**) showing the degree of flexibility by wobbling angles. **e**, Comparison of homoDiGb to the G5-006 crystallographic pattern. The pairs are aligned to one copy of Gb5-006. The angle of deviation is indicated. **f**, Comparison of heteroDiGb Gb5-006:GbEnhancer to the G5-006 crystallographic pattern. The pairs are aligned to one copy of Gb5-006. The angle of deviation is indicated. **g**–**i**,The interacting residues of the cysteine–cysteine interfaces in crystallo (**g**), in homoDiGb5-006 (**h**) and heteroDiGb Gb5-006:GbEnhancer (**i**), with blue dashes showing hydrogen bonds and red dashes showing the minimum distances (not counting hydrogen atoms) between the P46 or G47 and Q120 pairs. **j**, Top-view cartoon of the cysteine–cysteine interface for all three types. **k**–**m**, Side-view cartoons of RECQL5:homoDiGb (**k**), in crystallo RECQL5:Gb5-006 (**l**) and RECQL5:heteroDiGb:sfGFP (**m**), with key residues annotated and the minimum distances (not counting hydrogen atoms) between the P46 or G47 and Q120 pairs in red dashes, identical to **g**–**i**. Source data

The striking ease of application of the homoDiGb and heteroDiGb method to cryo-EM-enabled structure solution led us to probe its structural and biophysical origins, particularly in the noncanonical, side-chain-to-side-chain covalency that we exploit here to link protein modules (Fig. 6). These analyses revealed a remarkable balance. All six structures have DiGbs at distinct intra-Gb angles (Supplementary Fig. 11b and Supplementary Table 2), showing the remarkable compatibility of the side-chain-to-side-chain covalent linkage that is trapped at site 12. Yet, at the same time, within each structure, the DiGb interfaces exhibit <10° of wobble, suggesting that this interface is relatively constrained, thus enabling refinement with *C*_2_ symmetry (Fig. 6a–d, Extended Data Fig. 9 and Supplementary Table 2).

By closely inspecting the interfaces of RECQL5:homoDiGb and RECQL5:sfGFP:heteroDiGb that are both trapped by covalency and comparing them to the in crystallo interface that is trapped by crystallization, we discovered subtle differences in the interfaces (Fig. 6e,m), causing effectively different intra-DiGb angles (Fig. 6e,f). Interfaces in solution and in crystallo are approximately arranged in an isosceles triangular form with C12 at the top and the Q120 and P46 or G47 pairs at the bottom (Fig. 6g–j). However, comparing the minimum distances between the interacting bottom residue pairs (P46 or G47 and Q120), the in crystallo generated interface is aligned, whereas the in-solution interfaces are tilted (Fig. 6g–i). We noticed that, in both cases, Q123 always serves as a supporting residue, forming hydrogen bonds with the backbones on the opposing Gb (Fig. 6g–i), clearly highlighting the vital role of twinning in driving trapping. Because of tilt, hydrogen bonds only form in the closer half of the triangular interface (Fig. 6l,m), forming the interaction network together with the disulfide.

The side chains of crucial residues also change the DiGb interface. Apart from the triangular cysteine–cysteine interface discussed above (Fig. 6j and Supplementary Fig. 13), we observed a much narrower interface in the SPNS2:homoDiGb structure. Both sides of residue 120 on homoDiGb are lysines instead of the typical glutamine, causing the interface to be shifted to a new stable state, with P46 and G47 interacting instead with Q44 (Supplementary Fig. 13).

Lastly, we also observed clear structural evidence for covalent bond formation as a driving trapping force for formation of the DiGb complex. In the MBP:homoDiGb structure, there is a unique interface different from those discussed above or any class of nanobody crystal contacts^22^. The side-chain-to-side-chain covalent disulfide bond gives the Gbs a ‘U-turn’ in this joint, enabling the first β-sheet of both Gbs to come into contact but not close enough to form direct hydrogen bonds (Supplementary Fig. 13). Nonetheless, the resulting assembly proved constrained enough for cryo-EM structural determination, with or without local refinement, further highlighting the value of this kinetically controlled approach.

### DiGbs allow a ready method for structure determination

The Gb workflow appears robust because chromatographic, electrophoretic and stability characterization (Supplementary Figs. 3, 4, 8 and 14–16) reveal the preparation for complex formation and structure determination from a range of homogeneities from ~50% to >95%. The Gb-associated substitutions also do not destabilize nanobodies, in fact inducing a slight thermal stabilization both before and after DiGb formation (Supplementary Fig. 16). Yield of Gb varied on a case-by-case basis without any great magnitude changes between nanobody and Gb (Supplementary Fig. 14). Particles other than the target DiGb complexes, such as those containing a single target complexed with monomeric nanobody or DiGb, can be excluded during initial model generation. This subset of particles is also unable to generate high-resolution 3D maps because of suboptimal particle distribution, as we demonstrate here with the RecQL5:Gb complex (Supplementary Fig. 9). Moreover, seemingly close-packed samples have been previously observed for particles that appear monodisperse on SEC and nonetheless lead to successful structure determination; recent illustrative examples include protein–protein complexes^19,31^ and protein–RNA complexes^32^. For soluble protein complexes, we suggest concentration ranges from 0.5 to 1.0 mg ml^−1^. For membrane protein SPNS2, we used ~9.0 mg ml^−1^ for the homoDiGb complex and ~4.0 mg ml^−1^ for the heteroDiGb complexes.

The Gb interface has a pivotal role. Nanobody framework regions are highly conserved in both sequences and 3D structures; analyses have revealed that the root-mean-square deviation of over 150 nanobody V_H_H framework regions is ~ 1 Å (ref. ^33^). The key residues involved in the Gb interface (Q120, Q123, T125, Q14 and S7) are all conserved (>80%, >90%, >95%, >95% and >95% frequency, respectively). Residue 12, which we use here for side-chain covalency, is usually a leucine but can be a serine in 30% of all cases. This combined minimal level of side-chain alteration suggests that, when needed, Gb variations are applicable to different nanobodies; whilst some variations of intra-Gb angles might ensue, these will not be disruptive. To provide further insight into the engineering of the intra-Gb interface around residue 12, we also performed a mutational analysis of residue 125 that is directly adjacent (Fig. 6j). Using mountable crystal counts^22^ as a proxy for tolerance of the interface to disruption, we assessed mutational variation (Supplementary Fig. 17); this further supported M125 as a recommended DiGb substitution. Initial inspection speculatively suggests that methionine may best provide a hydrophobic cavity for the side-chain disulfide and putatively identified intra-DiGb interface sites Q120 and Q123 (Fig. 6j) for future optimization.

### Gbs allow assembly of higher-order multiGb systems

Our use of noncanonical, side-chain-to-side-chain covalency in the design of Gbs offers the possibility of higher-order multiplexing even beyond DiGbs (multiGbs). We, therefore, tested the potential that multiple targets might be assembled onto one scaffold with multiple pairs of heteroDiGbs to yet further increase cryo-EM throughput in a manner that would be difficult to achieve in conventional linear or fusion scaffolds.

For the assembly of the higher-order complex with the same target, we evaluated available nanobody-bound structures resolved to better than 3 Å. We selected the pentameric *Escherichia coli* Shiga toxin subunit Stx2aB (ref. ^34^) on the basis of a balance between avoidance of unwanted multimerization versus insufficient display constraints. Constructs for both Stx2aB and a Gb variant of a corresponding anti-Stx2aB nanobody were generated; the respective proteins were then expressed and purified as used as per the Gb workflow.

**Extended Data Fig. 10:**
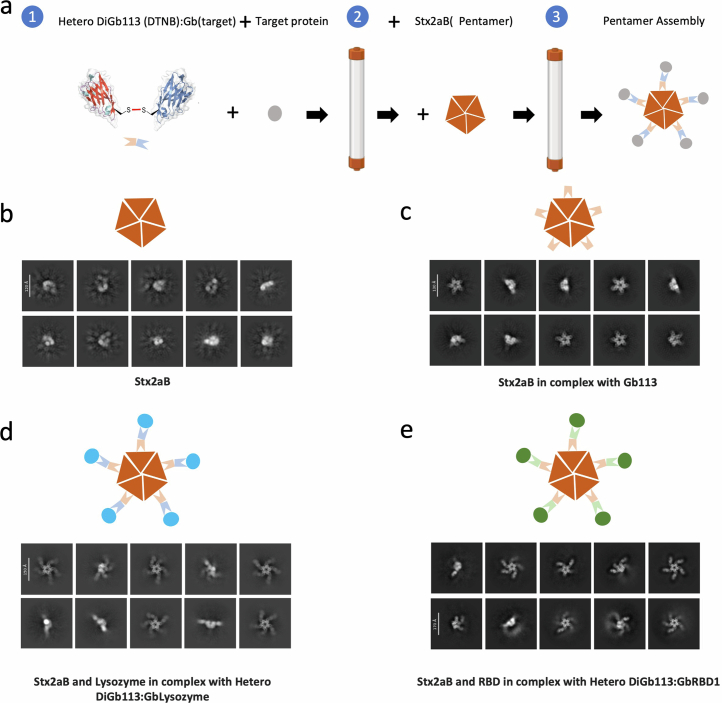
A penta-hetero-DiGb workflow that allows assembly of higher-order complexes. (**a**) Construction pipeline for pentameric complexes via hetero DiGembody. 2D classification results for (**b**) Stx2aB alone, (**c**) Stx2aB in complex with newly constructed anti-Stx2aB, Gb113, (**d**) Stx2aB and lysozyme in complex via penta-hetero DiGb113:GbLysozyme. (**e**) Stx2aB and SARS-CoV2-RBD in complex via penta-hetero-DiGb113:GbRBD1. Data collected from 300 kV Krios microscope.

Using this pentameric Stx2aB scaffold, we readily and successfully addressed two targets in multimeric form (SARS-CoV-2 receptor-binding domain (RBD) and lysozyme) using this novel penta-heteroDiGb system. In brief, heteroDiGbs and targets were first mixed and purified on SEC (Supplementary Fig. 8c,d); fractions corresponding to target:heteroDiGb complex were pooled, mixed directly with purified Stx2aB (Supplementary Fig. 8b) and then directly frozen onto cryo-EM grids. Comparisons of the cryo-EM 2D classification results clearly demonstrated that the targets were successfully loaded onto the pentameric Stx2aB scaffold (Extended Data Fig. 10).

## Discussion

Together, our results suggest that the success we see here in forming balanced, constrained assemblies is enabled by (1) homoDiGbs and heteroDiGbs settling into robust conformational minima at noncanonical dimerization interfaces with sufficiently balanced chemical flexibility and yet structural constraint and (2) the validity and robust nature of these interfaces being tested and reinforced by a chemically driven, kinetically controlled workflow that samples the equilibria that underpin the covalent interface-assisted events that are feasible in solution. Furthermore, the success of DiGbs for high-resolution structural solution is enabled by their relatively small mass contribution to the imaged complex while increasing the maximum particle diameter for better particle alignment (Supplementary Table 3).

A potential caveat to our Gb technology is the possibility for clashes between targets, which we observed for homoDiGb analysis of sfGFP and overcame through the use of an alternative heteroDiGb system. To estimate the general applicability of the DiGb method, we evaluated 973 published protein:nanobody complexes by placing them onto the homoDiGb scaffolds with the smallest (homoDiGb5-006) and the largest (homoDiGbD12) intra-Gb angles. Overall, 43.9% and 51.0% of the proteins incurred no clash when assembled onto these two scaffolds, respectively. Furthermore, 84.1% and 59.7% of the proteins tested incurred less than 5% of maximal clash (Methods). This suggests that the DiGb method should be a generally feasible approach for a variety of target proteins.

In summary, our plug-and-play DiGb strategy improves cryo-EM resolvability and resolution of small proteins through constraint and increased particle size with a simple and robust workflow and can even enable simultaneous structure determination of two challenging targets in one dataset. HeteroDiGbs, in particular, enable varied partner Gbs and target proteins that will likely dramatically change the chemical and physical properties of the complex and, thus, its behavior on the cryo-EM grid. Therefore, the screening of heteroDiGb complexes also provides a novel mechanism for potentially reducing preferred orientation, if needed. Future exploitation of this modular plug-and-play tool may allow examples of higher-order multiGb:target complexes or multispecific assemblies when combined with other scaffold proteins beyond those that we demonstrated here.

The kinetic interface trapping that underpins this method was discovered in crystallo and taken to its current modular form through rare, kinetically controlled noncanonical side-chain-to-side-chain covalent bond formation in solution. It should be noted, therefore, that the generic, rapid and modular nature of this method cannot and will not, essentially by definition, be achieved through traditional fusion protein methods. Moreover, our results also highlight that current dogmas of ‘tight’ *K*_D_ values, which indeed drive the assessment of most binding proteins, are less relevant to single-particle cryo-EM.

Lastly, our rapid implementation of the homoDiGb and heteroDiGb method suggests that it has the potential to become a powerful tool for cryo-EM SPA structure determination.

## Methods

### Conversion of the nanobody numbering to the IMGT scheme

Because of variable lengths of nanobody complementarity-determining regions (CDRs), the same positions on the nanobody scaffold have different residue numbers in different nanobodies. Therefore, we used the IMGT scheme^35^ through the server ANARCI^36^ for consistent numbering and clarity in the Gb substitutions (Fig. 1a and Supplementary Fig. 5).

### Expression and purification of RECQL5, sfGFP, SPNS2, MBP, lysozyme, Stx2aB and SARS-COV-2 RBD

RECQL5 was expressed and purified as previously described^22^. Briefly, the truncated RECQL5 protein (11–453) was subcloned into the pNIC-Bsa4 vector with a tobacco etch virus (TEV)-cleavable 6×His tag at the N terminus. The protein was expressed using the BL21-DE3-pRARE strain in autoinduction TB medium (Formedia) containing kanamycin and 0.01% antifoam 204 at 37 °C for 5.5 h followed by 40–44 h at 18 °C. The base buffer we used for purification contained 5% glycerol, 10 mM HEPES pH 7.5 and 500 mM NaCl. Bacteria were harvested by centrifugation at 4,000*g* and resuspended in three volumes of base buffer + 30 mM imidazole, 1% Triton, 0.5 mg ml^−1^ lysozyme and 10 μg ml^−1^ homemade benzonase, followed by storage in −80 °C freezer overnight for complete cell lysis. The purification started the next day with thawing the frozen pallets in a room-temperature water bath, followed by centrifugation at 5,000*g* for 1 h to obtain clear supernatant. The supernatant was then applied to Ni-NTA prepacked columns (GE healthcare) pre-equilibrated with base buffer + 30 mM imidazole. After thoroughly washing the Ni-NTA columns with base buffer, protein was eluted using 2.5 ml of base buffer + 500 mM imidazole directly into base-buffer-equilibrated PD-10 columns (GE healthcare). Next, 3.5 ml of base buffer was applied to PD-10 columns to elute the RECQL5 protein in base buffer. Then TEV protease was added to protein solution with a 1:10 mass ratio for overnight incubation; 20 mM imidazole was also added to the solution. On the next day, Ni-NTA columns were pre-equilibrated with base buffer + 20 mM imidazole and the RECQL5 solution with TEV was applied to the columns to get rid of RECQL5 with uncleaved 6×His tag, TEV protease and contaminants. Flowthrough fractions were collected and flash-frozen to make nanobody complexes. SPNS2 was expressed and purified in DDM as described previously^10^. The full-length human *SPNS2* gene was cloned into the pHTBV1.1 plasmid containing a C-terminal TEV protease cleavage site followed by EGFP, twin-Strep and 10×His affinity tags. Baculovirus was then generated according to the previously described protocols. The resulting baculovirus was used to infect Expi293F cells in Freestyle 293 expression medium (Gibco) in the presence of 5 mM sodium butyrate. Infected cells were grown in an orbital shaker for 48 h at 37 °C, 8% CO_2_ and 75% humidity, harvested by centrifugation, washed with PBS, flash-frozen and stored at −80 °C until further use. The cell pellets were resuspended in extraction buffer (300 mM NaCl, 50 mM HEPES pH 7.5 and 1% DDM) in the presence of cOmplete protease inhibitor cocktail tablets (Roche) and solubilized at 4 °C for 1 h with gentle rotation. The insoluble materials were pelleted at 50,000*g* for 40 min. The supernatants were incubated with pre-equilibrated TALON resin (Takara) and allowed to bind for 1 h at 4 °C. The resin was poured onto a gravity-flow column and washed with column buffer (300 mM NaCl, 50 mM HEPES pH 7.5 and 0.03% DDM (Anatrace) supplemented with 10 mM MgCl_2_, 1 mM adenosine triphosphate and 10 mM imidazole. Protein was eluted with elution buffer (300 mM NaCl, 50 mM HEPES pH 7.5, 300 mM imidazole and 0.03% DDM). The eluate was incubated with pre-equilibrated Strep-Tactin XT Superflow resin (IBA Lifesciences) for 1 h at 4 °C. The resin was poured onto a gravity-flow column and washed with column buffer. Protein was eluted with column buffer supplemented with 50 mM d-biotin, followed by tag cleavage with TEV protease overnight and reverse immobilized metal ion affinity chromatography purification using TALON resin. The tag-cleaved SPNS2 proteins were concentrated using a centrifugal concentrator with a 100-kDa cutoff (Sartorius) and subjected to SEC using a Superdex 200 10/300 GL column (GE Healthcare), pre-equilibrated with gel filtration buffer (150 mM NaCl, 20 mM HEPES pH 7.5 and 0.025% DDM). Peak fractions were pooled and concentrated for subsequent experiments.

The sfGFP and MBP protein was expressed using the BL21-DE3-pRARE strain in autoinduction TB medium (Formedia, AIMTB0260) containing 50 μg ml^−1^ kanamycin and 0.01% antifoam 204 at 37 °C for 5.5 h followed by 40–44 h at 18 °C. Bacteria were harvested by centrifugation at 4,000*g* and resuspended in a three cell pellet volumes of base buffer (5% glycerol, 10 mM HEPES pH 7.5 and 500 mM NaCl) supplemented with 30 mM imidazole, 1% Triton, 0.5 mg ml^−1^ lysozyme and 10 μg ml^−1^ benzonase and then stored in a −80 °C freezer overnight for freeze–thaw cell lysis. Cell pellets were thawed in a room temperature water bath and then clarified by centrifugation at 5,000*g* for 1 h. The supernatant was then applied to a Ni-NTA prepacked column (Cytiva, 11003399), pre-equilibrated with base buffer supplemented with 30 mM imidazole. After washing the Ni-NTA column with ten column volumes of base buffer, protein was eluted using 2.5 ml of base buffer supplemented with 500 mM imidazole and then immediately loaded onto a PD-10 column (Cytiva, 17-0851-01) that was equilibrated with base buffer. The sfGFP or MBP protein was then eluted from PD-10 columns. TEV protease at a 1:10 mass ratio and 20 mM imidazole were added to the protein solution and incubated overnight. The next day, Ni-NTA columns were pre-equilibrated with base buffer + 20 mM imidazole and the sfGFP or MBP solution with TEV was applied to the columns to remove sfGFP or MBP with uncleaved 6×His tag, TEV protease and contaminants. Eluted fractions were collected and flash-frozen until use.

Lysozyme was purchased from Sigma (L6876-1G) and dissolved in base buffer (5% glycerol, 10 mM HEPES pH 7.5 and 500 mM NaCl) before complexation with the binding homoDiGb.

The gene for Stx2aB (ref. ^34^) was synthesized (Twist Bioscience) and the amplified Stx2aB gene fragment was cloned into the pNIC-MBP2-LIC vector. Protein purification was performed using the same protocol as for sfGFP.

The SARS-CoV-2 RBD plasmid was generously provided by D. Zhou. Protein expression and purification were performed as previously described^37,38^. Briefly, the His-tagged SARS-CoV-2 RBD plasmid was transiently expressed in HEK293T cells (American Type Culture Collection, CRL-11268). The culture medium was concentrated using a QuixStand benchtop system and subsequently purified using a 5-ml HisTrap Nickel column (GE Healthcare). Glycan removal was followed by concentration for SEC (Superdex 200 Increase 10/300, GE) in a buffer containing 20 mM HEPES (pH 7.8) and 100 mM NaCl at 4 °C in a cold room. Protein fractions with a purity exceeding 90% were pooled, concentrated to 2 mg ml^−1^ using a 10-kDa-cutoff Centricon (Millipore) and stored at −80 °C.

### Generation of homoDiGbs

The genes of Gbs for homoDiGb generation were amplified from synthetic genes from Twist Bioscience (Gb5-006, GbD12, GbS2A4 (ref. ^39^), GbH12 (ref. ^10^), GbLysozyme, GbHIV and GbRBD3) or existing clones with Gb mutations implemented in the primers (GbEnhancer and GbMBP)^40^. GbEnhancer, GbLysozyme, GbHIV and GbMBP had the minimal Gb substitutions S7N;L12C;Q14K;T125M implemented. Gb5-006 had solubility substitutions G40T;Q49E;L52W;I101V plus K84E in addition to S7N;L12C;Q14K;T125M. GbD12 had S7N;L12C;Q14K;K84E;P123Q;T125M implemented. GbS2A4 had Q5V;S7N;L12C;Q14K;Q44R;T125M implemented. GbH12 had L2V;S7N;L12C;Q14K;K84E;P123Q;T125M implemented. GbRBD3 had S7N;L12C;Q14K;T125M;K84E implemented. They all shared the core substitutions of S7N;L12C;Q14K;T125M. NbH12 (wild-type GbH12) was generated in the same screening batch as NbD12 (wild-type GbD12)^10^. The amplified GbMBP gene fragment was cloned onto the pNIC-NHStIIT vector, the GbGFP gene fragment was cloned onto the pNIC-GST-bio vector and all other amplified genes were subsequently cloned onto the pNIC-MBP2-LIC vector by ligation-independent cloning. The Gbs were produced as previously described except that no reducing agent was used throughout purification. The purified monomeric Gbs were exchanged into 50 mM sodium phosphate (Na_2_HPO_4_) buffer (pH 8.0) using PD MiniTrap G25 columns (Cytiva, 28918007), snap-frozen and stored at −80 °C until further use.

To generate homoDiGbs, purified Gb at 2 mg ml^−1^ in 50 mM Na_2_HPO_4_ pH 8, was treated with 50 μM Cu(II) acetate and the resulting solution was incubated at 37 °C temperature for 30 min. Conversion was monitored by intact mass spectrometry on Waters G2-XS quadrupole time-of-flight (QToF) mass spectrometers equipped with a Waters Acquity ultrahigh-performance liquid chromatograph (UPLC). Separation was achieved using a Thermo Scientific ProSwift RP-2H monolithic column (4.6 mm × 50 mm) with water + 0.1% formic acid (solvent A) and acetonitrile + 0.1% formic acid (solvent B) as the mobile phases over a 5-min linear gradient. Spectra were deconvoluted using MassLynx 4.1 (Waters). Upon completion, DiGb was purified by SEC chromatography using a Superdex 75 Increase 10/300 column (Cytiva, 29-1487-21). Detailed data for Gb5-006, GbD12, GbLysozyme and GbMBP are shown in Extended Data Fig. 1a–d (left). Additional pairs of homoDiGbs are shown in Fig. 1a–d (right). The Gb sequences and the corresponding primers used for cloning are listed in Supplementary Tables 4 and 5.

### Generation of heteroDiGbs

The genes of Gbs for heteroDiGb generation were amplified from synthetic genes from Twist Bioscience (Gb5-006, GbH12 (ref. ^10^), GbRBD1 (ref. ^41^) and GbRBD6 (ref. ^41^)) or existing clones with Gb substitutions implemented in the primers (GbEnhancer and GbC4)^10^. Gb113 had the minimal Gb substitutions S7N;L12C;Q14K;T125M implemented. GbC4 had L2G;S7N;L12C;Q14K;T125M implemented. GbRBD1 and GbRBD6 had S7N;L12C;Q14K;T125M;K84E implemented. They all shared the core substitutions of S7N;L12C;Q14K;T125M. NbC4 (wild-type GbC4) was generated in the same screening batch as NbD12 (wild-type GbD12)^10^. All amplified genes were subsequently cloned onto the pNIC-MBP2-LIC vector by ligation-independent cloning^42^. The Gbs were produced as previously described except that no reducing agent was used throughout purification^22^. The purified monomeric Gbs were exchanged into 50 mM sodium phosphate (Na_2_HPO_4_) buffer pH 8.0 using PD MiniTrap G25 columns (Cytiva, 28918007), snap-frozen and stored at −80 °C until further use.

For the generation of heteroDiGbs, the first Gb (GbA) at 1.0 mg ml^−1^ in 50 mM Na_2_HPO_4_ pH 8.0 was treated with DTT at a tenfold molar concentration of GbA at room temperature. After 15 min, DTT was removed from GbA using PD MiniTrap G25 columns (Cytiva, 28918007) equilibrated in 50 mM Na_2_HPO_4_ pH 8.0. GbA was then treated with a tenfold molar concentration of DTNB at room temperature. After 30 min, DTNB was removed by buffer exchange using G25 columns in 50 mM Na_2_HPO_4_ pH 8 and concentrated with Ultra 0.5 ml 3-kDa centrifugal filters (Amicon, UFC500308). Conversion of GbA’s C12 to C12TNB was confirmed on Waters G2-XS QToF mass spectrometers equipped with a Waters Acquity UPLC. Separation was achieved using a Thermo Scientific ProSwift RP-2H monolithic column (4.6 mm × 50 mm) with water + 0.1% formic acid (solvent A) and acetonitrile + 0.1% formic acid (solvent B) as the mobile phases over a 5-min linear gradient. Spectra were deconvoluted using MassLynx 4.1 (Waters). Samples of GbB at 1.0 mg ml^−1^ were reduced with DTT using the same protocol as for GbA, desalted using minitrap G25 columns and then mixed with GbA-C12TNB at a twofold molar concentration and incubated for 30 min at 37 °C. Small-molecule byproducts from the resulting mixture were removed using a G25 column in a buffer containing 50 mM Na_2_HPO_4_ pH 8.0, flash-frozen in liquid nitrogen and kept at –80 °C until further use. Detailed data for Gb5-006:GbEnhancer, GbC4:GbEnhancer and additional pairs of heteroDiGbs are shown in Extended Data Fig. 2.

### In vitro reconstitution of DiGb:target complexes

All proteins before this stage were in a buffer without reducing agents as described above; thus, all proteins hereafter are under nonreducing conditions.

To prepare complexes of RECQL5 with the homoDiGb Gb5-006, DiGb was mixed with RECQL5 at a molar ratio of 1:2. The complex was PEGylated with NHS-PEG_4_-azide (Thermo Fisher) at a final concentration of 2 mM. This reaction was performed on ice for 2 h, quenched by adding 50 mM Tris buffer pH 8.0 and subsequently purified by SEC on a Superdex 200 10/300 GL Increase column (Cytiva, 28-9909-44), pre-equilibrated in 20 mM HEPES pH 7.5 and 150 mM NaCl. The dimer peak was pooled, concentrated by 10-kDa Vivaspin 20 centrifugal concentrators (Vivaproducts, VS2001) and used for cryo-EM specimen preparation (Supplementary Fig. 3a). The same procedure was applied to lysozyme (Supplementary Fig. 3c) and MBP (Supplementary Fig. 3d).

To prepare complexes of sfGFP with the homoDiGb GbEnhancer, DiGb was mixed with sfGFP at a molar ratio of 1:2 and subsequently purified by SEC on a Superdex 200 10/300 GL Increase column (Cytiva, 28-9909-44), pre-equilibrated in 20 mM HEPES pH 7.5 and 150 mM NaCl. The dimer peak was pooled, concentrated by 10-kDa Vivaspin 20 centrifugal concentrators (Vivaproducts, VS2001) and used for cryo-EM specimen preparation.

To prepare complexes of SPNS2 with the homoDiGb GbD12, the purified DiGb was first supplemented with 0.025% DDM (Anatrace, D310LA) and then mixed with SPNS2 at a molar ratio of 1:2. The complex was then purified by SEC on a Superdex Increase 200 10/300 GL column (Cytiva. 28-9909-44), pre-equilibrated in 20 mM HEPES pH 7.5, 150 mM NaCl and 0.025% DDM. The dimer peak was pooled, concentrated by 100-kDa Vivaspin 20 centrifugal concentrators (Vivaproducts, VS2041) and used for cryo-EM specimen preparation (Supplementary Fig. 3b).

The RECQL5:heteroDiGb:sfGFP complex was prepared by first mixing Gb5-006:GbEnhancer heteroDiGb with RECQL5 at a molar ratio of 1:1.5. The complex was PEGylated with NHS-PEG_4_-azide (Thermo Fisher) at a final concentration of 2 mM on ice for 2 h and quenched by adding 50 mM Tris buffer pH 8.0. The RECQL5:heteroDiGb complex was then purified by SEC on the Superdex Increase 75 10/300 GL column (Cytiva, 29-1487-21) pre-equilibrated in 20 mM HEPES pH 7.5 and 150 mM NaCl. The peak corresponding to the heteroDiGb and RECQL5 complex was pooled (Supplementary Fig. 4b, left), mixed with sfGFP at a molar ratio of 1:1.5, and further purified by SEC on the Superdex Increase 75 10/300 GL column. The peak containing both targets was then pooled, concentrated by 10-kDa Vivaspin 20 centrifugal concentrators (Vivaproducts, VS2001) and used for cryo-EM specimen preparation (Supplementary Fig. 4b, right).

The SPNS2:heteroDiGb:sfGFP complex was prepared by first mixing GbC4:GbEnhancer heteroDiGb with sfGFP at a molar ratio of 1:1.5. The sfGFP:heteroDiGb complex was then purified by SEC on the Superdex Increase 200 10/300 GL column (Cytiva, 28-9909-44), pre-equilibrated in 20 mM HEPES pH 7.5, 150 mM NaCl and 0.025% DDM. The peak corresponding to the heteroDiGb and sfGFP complex was pooled (Supplementary Fig. 4a, left), mixed with SPNS2 at a molar ratio of 1:1 and further purified by SEC on the Superdex Increase 200 10/300 GL column. The peak containing both targets was then pooled, concentrated by 100-kDa Vivaspin 20 centrifugal concentrators (Vivaproducts, VS2041) and used for cryo-EM specimen preparation (Supplementary Fig. 4a, right).

### Cryo-EM specimen preparation and data acquisition

Cryo-EM grids of the RECQL5:homoDiGb, sfGFP:homoDiGb, MBP:homoDiGb, lysozyme:homoDiGb and RECQL5:heteroDiGb:sfGFP complexes were prepared by applying freshly purified complexes to Au C‑flat, 2/1, 200-mesh grids (Jena Bioscience, X-302-AU200) at 0.8, 0.75, 2.5, 0.85 and 1.1 mg ml^−1^, respectively. Grids were blotted using a Vitrobot (FEI) at 4 °C and 100 % humidity for 4 s with a force of −6 and a 5-s waiting time, followed by plunging into liquid ethane.

Cryo-EM grids of the SPNS2:homoDiGb and SPNS2:heteroDiGb:sfGFP complexes were prepared by applying freshly purified complex to Quantifoil Copper, 1.2/1.3, 300-mesh grids (Quantifoil) at 9.0 and 4.2 mg ml^−1^, respectively. Grids were blotted using a Vitrobot (FEI) at 4 °C and 100 % humidity for 8 s with force of −10 and a 5-s waiting time, followed by plunging into liquid ethane.

Cryo-EM data were collected using a FEI Titan Krios operating at 300 kV with a Gatan K3 with GIF Quantum camera or Falcon 4 with GIF Quantum camera. All data were automatically collected using EPU software (Thermo Fisher) with a defocus range targeting −1.5 to −2.5 µm for SPNS2:homoDiGb and SPNS2:heteroDiGb:sfGFP complexes, −1.2 to −2.6 µm MBP:homoDiGb and lysozyme:homoDiGb or −1.5 to −3 µm for RECQL5:homoDiGb and RECQL5:heteroDiGb:sfGFP complexes. Other parameters such as magnification, total dose and frames used varied between different sample collections and are provided in Supplementary Table 1.

### Image processing of electron micrographs

All datasets were subject to a similar protocol for image processing and reconstruction by cryoSPARC^43^. Raw micrographs were motion-corrected, contrast transfer function (CTF)-estimated and curated manually to remove those images with poor image quality (that is, CTF fit > 4 Å, ice thickness > 1.1 and astigmatism > 1,000). For each dataset, 500 micrographs were then used for blob picking and several rounds of 2D classifications. The particles with the best 2D class averages were selected for Topaz training and particle picking using all micrographs. After 2D classifications of Topaz-picked particles, all particles from well-resolved 2D classes were merged after removing duplicated particles. The output particles were used for ab initio reconstruction, followed by heterogeneous refinement. Well-resolved classes were selected for nonuniform refinement (with *C*_2_ symmetry applied to RECQL5:homoDiGb, SPNS2:homoDiGb and MBP:homoDiGb and *C*_1_ symmetry applied to lysozyme:homoDiGb, SPNS2:heteroDiGb:sfGFP and RECQL5:heteroDiGb:sfGFP complexes), followed by local refinement with target-specific masks. The masks were generated using the ‘molmap’ command in UCSF Chimera^44^ on the basis of roughly docked atomic models for the target proteins. For the homoDiGb complexes (except lysozyme:homoDiGb), the particles were symmetry-expanded, followed by further rounds of 3D classifications. The particles from best-resolved classes were then merged for a final round of local refinement. Maps were further sharpened by DeepEMhancer^45^. RECQL5:Gb5-006 used the same processing pipeline as for the processing of RECQL5:homoDiGb5-006 complex.

In all cases, the resolution was determined by gold-standard Fourier shell correlation. The local resolution estimation was calculated by cryoSPARC and presented using UCSF Chimera^44^ on the basis of the output maps from cryoSPARC. Next, 3D conformational variability analysis was carried out using 3DVA^46^. The processing details for each dataset are shown in Extended Data Figs. 3c, 4c, 5c, 6c, 7c and 8c.

### Model building and refinement

Initial models of RECQL5 and Gb5-006 were obtained from deposited crystal structures (Protein Data Bank (PDB) 7ZMV)^22^. Those of sfGFP and GbEnhancer were obtained by modifying the existing models (PDB 3K1K). For lysozyme and GbLysozyme, the initial model was obtained from a deposited structure (PDB 6JB2). For MBP and GbMBP, the initial model was obtained from a deposited structure (PDB 5M13). Models of SPNS2, GbD12 and GbC4 used the SPNS2:NbD12 structure published previously (PDB 8QV6)^10^ as starting models. Model were rebuilt as necessary in Coot^47^ and then refined with PHENIX real-space refinement^48^.

### Affinity measurement of nanobodies and Gbs

Purified RECQL5 and sfGFP proteins were biotinylated using an EZ-Link Sulfo-NHS-LC-biotinylation kit (Thermo Scientific, 21435) following the manufacturer’s instructions. Biolayer interferometry (BLI) was then performed using Octet RED384. All experiments were performed using buffer containing 10 mM HEPES and 150 mM NaCl (pH 7.5) for dilution and incubation. All incubation was conducted while shaking at 1,000 rpm at room temperature. All experiments were performed in triplicate.

For affinity measurements of anti-RECQL5 nanobody, Gb5-006 (monomer), the biotinylated target protein solution RECQL5 was diluted to 3 μM. Streptavidin-coated biosensors (ForteBio, lot 2104023111) were first incubated in buffer for 1 min, then dipped into protein solution for 2 min and returned to buffer for 1 min. The arrays of sensors were then dipped into eight concentrations (including a zero concentration for subtraction) of nanobody and Gb5-006 for 10 min for the association step. Finally, the sensors were transferred to buffer for the dissociation step for 5 min to complete the measurement. The concentration gradient for anti-RECQL5 nanobody was 9 μM, 4.5 μM, 2.250 μM, 1.125 μM, 562.5 nM, 281.25 nM, 140.625 nM and 0. The concentration gradient for anti-RECQL5 Gb5-006 was 17.7 μM, 10.1 μM, 4.3 μM, 1.9 μM, 620.6 nM, 206.9 nM, 69.0 nM and 0.

For affinity measurements of anti-RECQL5 homoDiGb, the biotinylated target protein solution RECQL5 was diluted to 1.62 μM. Streptavidin-coated biosensors (ForteBio) were first incubated in buffer for 1 min, then dipped into protein solution for 2 min and returned to buffer for 2 min. The arrays of sensors were then dipped into eight concentrations (including a zero concentration for subtraction) of homoDiGb5-006 for 5 min for the association step. Finally, the sensors were transferred to buffer for the dissociation step for 5 min to complete the measurement. The concentration gradient for anti-RECQL5 homoDiGb5-006 was 4.3 μM, 2.1 μM, 1.1 μM, 531.25 nM, 265.6 nM, 132.8 nM, 66.4 nM and 0.

For affinity measurements of anti-sfGFP GbEnhancer, the biotinylated target protein solution sfGFP was diluted to 4.5 μM. Streptavidin-coated biosensors (ForteBio, 18-5019) were first incubated in buffer for 1 min, then dipped into protein solution for 2 min and returned to buffer for 2 min. The arrays of sensors were then dipped into eight concentrations (including a zero concentration for subtraction) of GbEnhancer for 5 min for the association step. Finally, the sensors were transferred to buffer for the dissociation step for 5 min to complete the measurement. The concentration gradient for anti-GFP GbEnhancer was 3 μM, 1 μM, 333 nM, 111 nM, 37.3 nM, 12.4 nM, 4.1 nM and 0.

For data processing, all traces with Gb and nanobody concentrations above 0 were subtracted by the values from the zero-concentration trace and aligned to the beginning of the association step. Nonlinear regression was performed using Graphpad Prism 10.2.3 to estimate the dissociation constants of the Gbs and nanobodies.

### Dynamic analysis of the DiGb interface

Selected particles used for the final rounds of nonuniform refinement^49^ for the dimer structures of RECQL5:homoDiGb, SPNS2:homoDiGb, RECQL5:heteroDiGb:sfGFP and SPNS2:heteroDiGb:sfGFP structures were used as inputs of 3DVA on cryoSparc^43^, yielding 60 density maps. The atomic models for RECQL5:homoDiGb, SPNS2:homoDiGb, RECQL5:heteroDiGb:sfGFP and SPNS2heteroDiGb:sfGFP were then split at the DiGb interface and each model was half-docked into the 3D variability density maps using ChimeraX^50^.

The movements of the DiGb interfaces were analyzed by first aligning a reference (static) Gb. Vectors $\stackrel{\rightharpoonup}{{\bf{D}}_{i}}(i=\mathrm{1,2},\ldots ,60)$ and its average $\stackrel{\rightharpoonup}{{\bf{D}}_{a}}=\mathop{\sum }\nolimits_{1}^{60}\stackrel{\rightharpoonup}{{\bf{D}}_{i}}$/60, defined by each substituent Gb’s center of mass and the Cα atoms of C12, were then calculated for both static and the unaligned (mobile) Gb. Motion within a single DiGb complex was quantified as the wobble angle ${\beta }_{i}=\arccos (\frac{\stackrel{\rightharpoonup}{{\bf{D}}_{a}}\bullet \stackrel{\rightharpoonup}{{\bf{D}}_{i}}}{|\stackrel{\rightharpoonup}{{\bf{D}}_{a}}|\bullet |\stackrel{\rightharpoonup}{{\bf{D}}_{i}}|})(i=\mathrm{1,2},\ldots ,60)$ using the mobile Gb vectors, while the intra-DiGb angle was calculated between the $\stackrel{\rightharpoonup}{{\bf{D}}_{a}}$ of the static and mobile Gbs.

### Estimation of the applicability of homoDiGb scaffolds to existing structures by clash scoring

In total, 973 structures of target:nanobody complexes from the PDB were used to examine the clashing potential when dimerized using homoDiGbs. In silico (Pymol 3), two copies of the target:nanobody complex were aligned against two Gb copies of exemplar homoDiGbs, homoDiGbD12 or homoDiGb5-006. Target protein atoms within a sphere of 4 Å from the other copy were scored as ‘clashed’ and a corresponding clashing score was calculated on the basis of the total number of clashing atoms.

### Thermal stability characterization by nanoDSF

Nanobodies, Gbs and homoDiGbs were diluted to around 0.1 mg ml^−1^ and incubated at room temperature before measurement in a Prometheus NT.48 device (Nanotemper). The excitation power was 100% and temperature gradient was from 20 to 95 °C with a slope of 1 °C min^−1^. Data were analyzed using PR ThermControl software (Nanotemper). All measurements were performed in technical triplicates.

### Workflow for a pentameric DiGb imaging scaffold

For the generation of heteroDiGbs, heteroDiGb113:GbLysozyme and heteroDiGb113:GbRBD1 were applied through the same pipeline used for generating heteroDiGb Gb5-006:GbEnhancer and heteroDiGb GbC4:GbEnhancer except that, to avoid side products of homoDiGb113, Gb113 was labeled with DTNB and then reacted with GbLysozyme and GbRBD1.

For the in vitro reconstitution of DiGb:target complexes, the lysozyme:heteroDiGb:Stx2aB complex was prepared by first mixing Gb113:GbLysozyme heteroDiGb with lysozyme at a molar ratio of 1:3. The complex was PEGylated with NHS-PEG_4_-azide (Thermo Fisher) at a final concentration of 2 mM on ice for 2 h and quenched by adding 50 mM Tris buffer (pH 8.0). The lysozyme:heteroDiGb complex was then purified by SEC on the Superdex 200 Increase 5/150 (GE), pre-equilibrated in 20 mM HEPES (pH 7.5) and 150 mM NaCl. The peak corresponding to the heteroDiGb and lysozyme complex was concentrated (Supplementary Fig. 8c), mixed with Stx2aB at a molar ratio of 1:0.8 and used for cryo-EM specimen preparation. The RBD:heteroDiGb:Stx2aB complex was prepared by first mixing Gb113:GbRBD1 heteroDiGb with RBD at a molar ratio of 2:1. The complex was PEGylated with NHS-PEG_4_-azide (Thermo Fisher) at a final concentration of 2 mM on ice for 2 h and quenched by adding 50 mM Tris buffer (pH 8.0). The RBD:heteroDiGb complex was then purified by SEC on the Superdex Increase 75 10/300 GL column (Cytiva, 29-1487-21), pre-equilibrated in 20 mM HEPES pH 7.5 and 150 mM NaCl. The peak corresponding to the heteroDiGb and RBD complex was concentrated (Supplementary Fig. 8d), mixed with Stx2aB at a molar ratio of 1:0.8 and used for cryo-EM specimen preparation.

For cryo-EM specimen preparation and data acquisition, cryo-EM grids of the Stx2aB, Stx2aB:Gb113 and lysozyme:heteroDiGbLysozyme:Gb113:Stax2aB complexes were prepared by applying freshly purified complex to Quantifoil copper, 1.2/1.3, 300-mesh grids (Quantifoil) at 0.5, 0.6 and 0.8 mg ml^−1^, respectively. RBD:heteroDiGbRBD1:Gb113:Stax2aB complexes were prepared by applying freshly purified complex to Ultralfoil, 1.2/1.3, 300-mesh grids (Quantifoil) at 0.75 mg ml^−1^. Grids were blotted using a Vitrobot (FEI) at 4 °C and 100% humidity for 3.5 s with force of −15 on 3 µl samples, followed by plunging into liquid ethane. Cryo-EM data were collected using a FEI Titan Krios operating at 300 kV with a Gatan K3 with GIF Quantum camera or Falcon 4 with GIF Quantum camera. All data were automatically collected using EPU software (Thermo Fisher) with a defocus range targeting −1.6 to −2.6 µm for Stx2aB, Stx2aB:Gb113, lysozyme:heteroDiGbLysozyme:Gb113:Stax2aB and RBD:heteroDiGbRBD1:Gb113:Stax2aB complexes with around 1,500 micrographs. RECQL5:Gb5-006 was automatically collected using EPU software (Thermo Fisher) with defocus range targeting −1.5 to −2.5 µm with around 5,000 micrographs.

### Reporting summary

Further information on research design is available in the Nature Portfolio Reporting Summary linked to this article.

## Online content

Any methods, additional references, Nature Portfolio reporting summaries, source data, extended data, supplementary information, acknowledgements, peer review information; details of author contributions and competing interests; and statements of data and code availability are available at 10.1038/s41589-025-01972-7.

## Supplementary information

Supplementary Information Supplementary Figs. 1–18, Tables 1–5 and uncropped gels.

Reporting Summary

Supplementary Code 1 Python scripts for clash prediction analyses.

## Source data

Source Data Fig. 5 Statistical source data.

Source Data Fig. 6 Statistical source data.

Source Data Extended Data Fig. 9 Statistical source data.

## Data Availability

Cryo-EM density maps were deposited to the EMDB under accession codes EMD-19331, EMD-19332, EMD-19333, EMD-19334, EMD-19335, EMD-19336, EMD-19337, EMD-19338, EMD-19339, EMD-19340, EMD-50430, EMD-50432, EMD-50433 and EMD-50525 and corresponding coordinate files were deposited to the PDB under accession codes 8RL5, 8RL6, 8RL7, 8RL8, 8RL9, 8RLA, 8RLB, 8RLC, 8RLD, 8RLE, 9FGV, 9FGX, 9FGY and 9FQK. The map and model identifiers are detailed in Extended Data Table 1. All other data are available upon request. Source data are provided with this paper.

## Code availability

Python scripts for clash prediction analyses are provided in Supplementary Code 1.
