# Supplementary material for: Covalently constrained ‘Di-Gembodies’ enable parallel structure solutions by cryo-EM
Source: Nat Chem Biol. 2025 Aug 15;22(1):69–76. doi: 10.1038/s41589-025-01972-7 (PMC12435805; doi:10.1038/s41589-025-01972-7)
Supplement: Supplementary file 1 — Supplementary Figs. 1–18, Tables 1–5 and uncropped gels. [file 41589_2025_1972_MOESM1_ESM.pdf]

nature chemical biology

Supplementary information https://doi.org/10.1038/s41589-025-01972-7

Covalently constrained 'Di-Gembodies' enable parallel structure solutions by cryo-EM

In the format provided by the authors and unedited

Table of Contents

Supplementary Figures 1-18
Supplementary Tables 1-5
Supplementary Note: Source Data of Mass Spectrometry

Supplementary Fig. 1

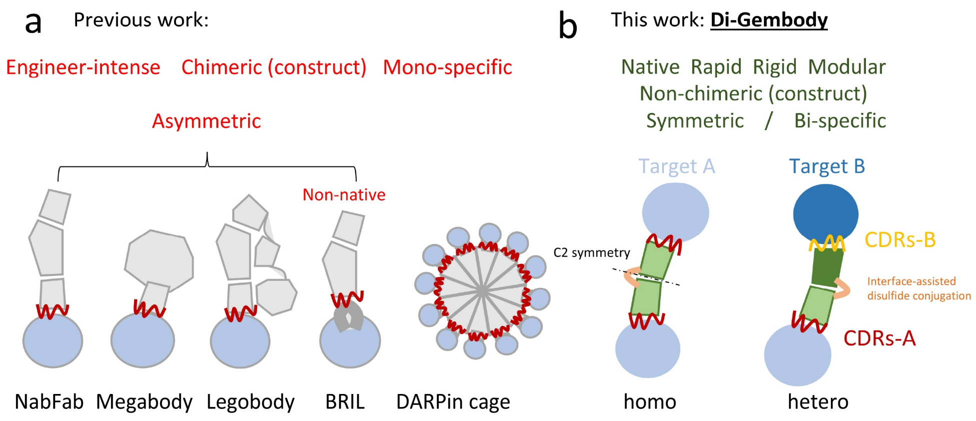

Supplementary Fig. 1 Schematic Comparisons of Di-Gembody and existing tools to make small proteins into larger complexes. (a) Previous work. Scaffold proteins are coloured grey, target proteins are in blue and CDRs are in red. To overcome the size limit of the cryo-EM single particle method, various forms of fiducial proteins have been created, which include NabFab, Megabody, Legobody, synthetic antibodies against BRIL fusions, and DARPin cages, all of which can be complemented by the Gembody method. (b) Di-Gembody (this work). Monomeric Gembodies are coloured deep and light green, target proteins are in deep and light blue, disulfides are in orange, and CDRs are in red and yellow. The Di-Gembody method aims to overcome those shortcomings and integrate the functions of fiducials created so far. It avoids complicated chimera constructs, maintains the fraction of the protein of interest (**Supplementary Table 3)** and is highly modular. The latter also empowers the bi-specific with a modular protein assembly function. Notably that can extend even to mutimeric use (see **Extended Data Figure 10**) without intricate redesign and optimization simply derived from a natural multimeric complex.

Supplementary Fig. 2

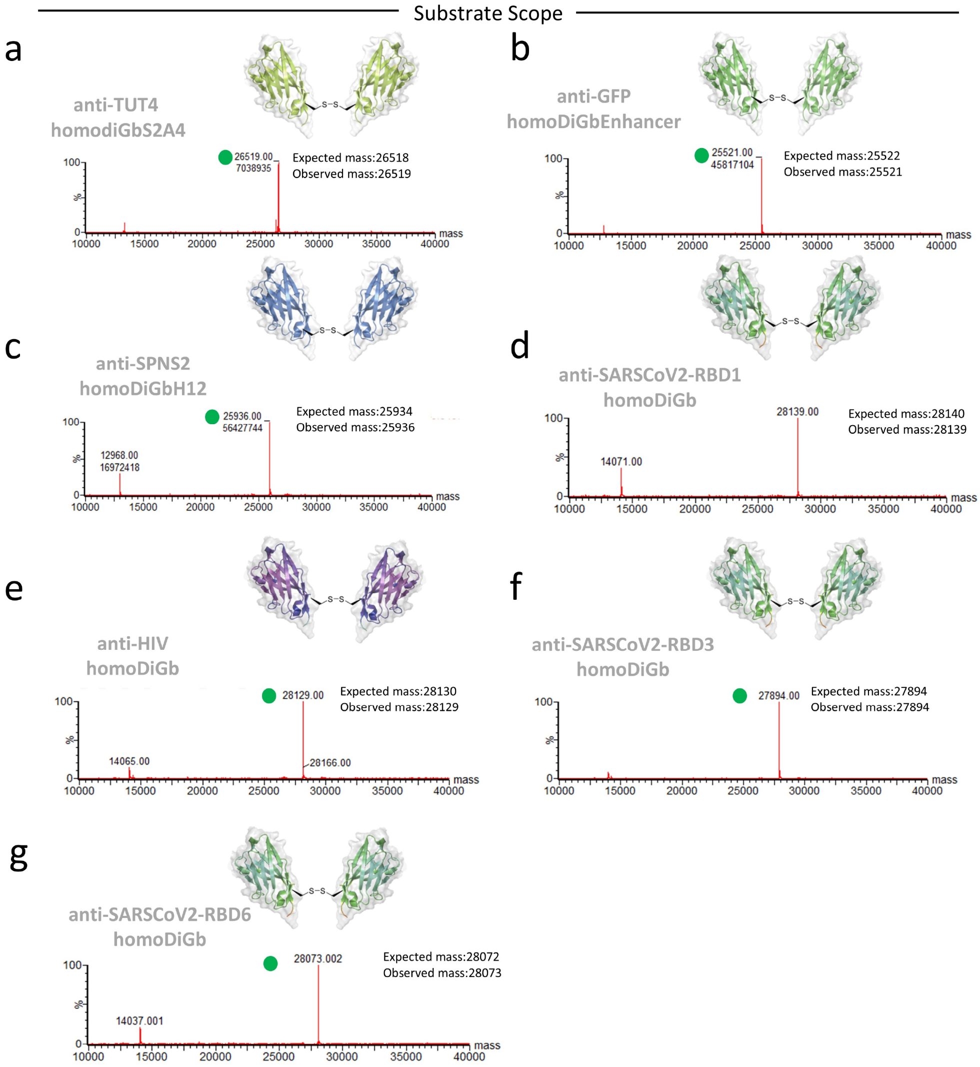

Supplementary Fig. 2 Modular generation of additional pairs of homo Di-Gembodies (homoDiGbs). (a) GbS2A4 (b) GbEnhancer (c) GbH12 (d) GbRBD1 (e) GbHIV (f) GbRBD3 (g) GbRBD6. Mass Spec peaks for homoDiGb are indicated by blobs in green, respectively.

Supplementary Fig. 3

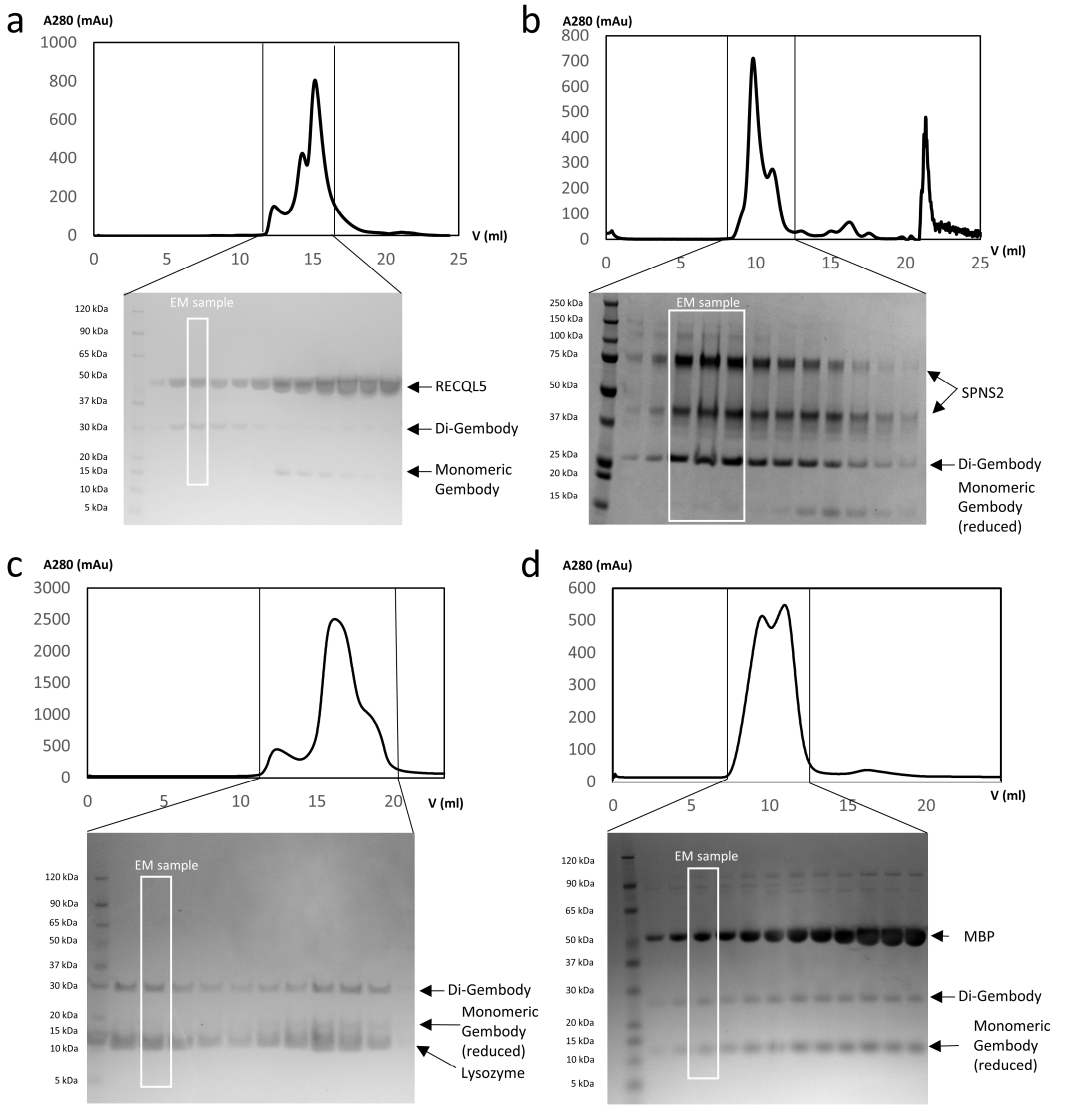

Supplementary Fig. 3 Homo dimer complex reconstitutions with size exclusion chromatography (SEC). (a) RECQL5 in complex with the homoDiGb5-006, EM sample obtained with ~55-80% homogeneity. (b) SPNS2 in complex with the homoDiGbD12, ~50-65%. (c) Lysozyme in complex with the homoDiGbLysozyme, >95%. (d) MBP in complex with the homoDiGb MBP, ~80->95%. The trials above performed twice with similar results. See Supplementary Fig. 18 for uncropped gels.

Supplementary Fig. 4

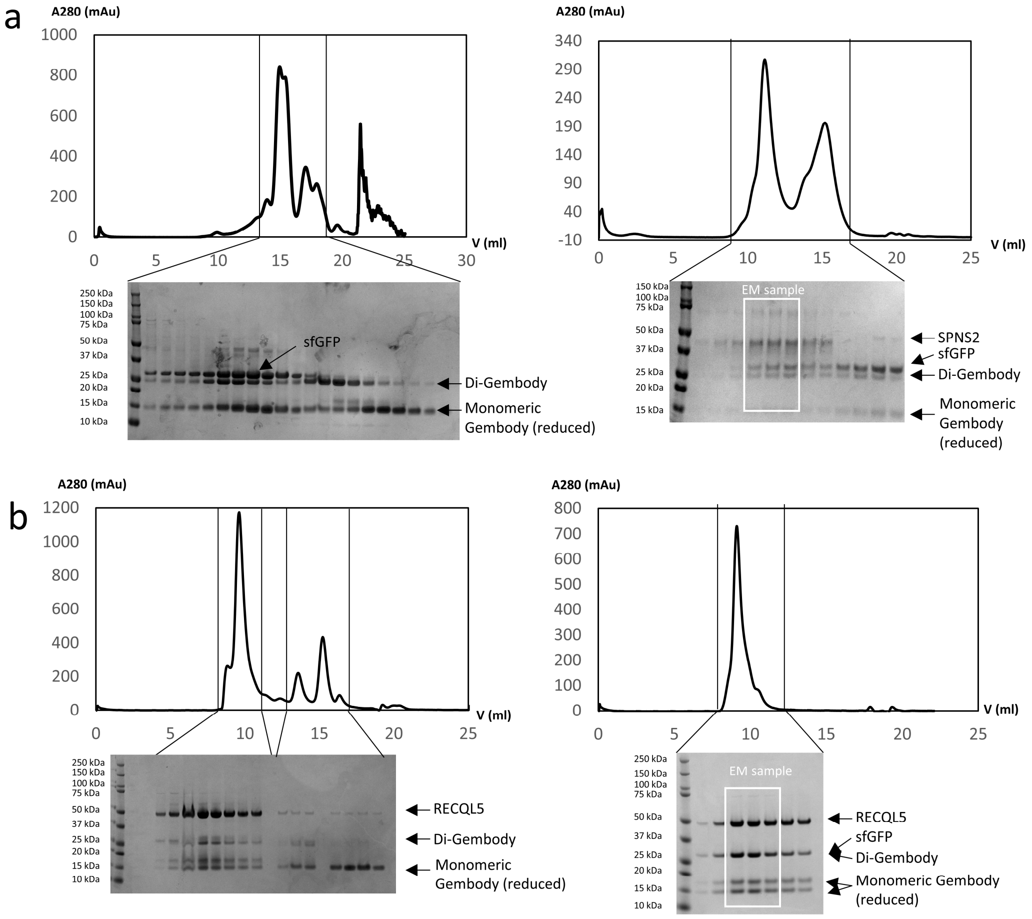

Supplementary Fig. 4 Hetero dimer complex reconstitutions with size exclusion chromatography (SEC). (a) Two-step purification of SPNS2:heteroDiGb:sfGFP complex with >95% homogeneity. (b) Two-step purification of RECQL5:heteroDiGb:sfGFP complex with >95% purity. The trials above performed twice with similar results. See Supplementary Fig. 18 for uncropped gels.

Supplementary Fig. 5

a

Gembody	No. of Amino	Theoretical Molecular	N-terminal head	IMGT numbering scheme																																						
				1	2	3	4	5	6	7	8	9	10	11	12	13	14	15	16	17	18	19	20	21	22	23	24	25	26	27	28	29	30	31	32	33	34	35	36	37	38	39
Gb5-006	127	13765.23	SMA	Q	V	Q	L	V	E	N	G	G	-	G	C	V	K	A	G	G	S	L	R	L	S	C	A	A	S	G	S	I	F	-	-	-	-	S	I	N	R	M
GbD12	130	14044.94	SMA	Q	V	Q	L	V	E	N	G	G	-	G	C	V	K	A	G	G	S	L	R	L	S	C	A	A	S	G	R	L	L	-	-	-	-	S	W	Y	D	M
GbS2A4	120	13262.11	SMA	Q	V	Q	L	V	E	N	G	G	-	G	C	V	K	A	G	G	S	L	R	L	S	C	A	A	S	G	T	I	F	-	-	-	-	T	Y	F	V	M
GbEnhancer	115	12763.23	S	Q	V	Q	L	V	E	N	G	G	-	A	C	V	K	P	G	G	S	L	R	L	S	C	A	A	S	G	F	P	V	-	-	-	-	N	R	Y	S	M
GbH12	118	12970.71	SMA	Q	V	Q	L	V	E	N	G	G	-	G	C	V	K	P	G	G	S	L	R	L	S	C	A	A	S	G	S	R	F	-	-	-	-	S	D	N	T	M
GbLysozyme	131	14155.76	S	D	V	Q	L	V	E	N	G	G	-	G	C	V	K	A	G	G	S	L	R	L	S	C	A	A	S	G	S	T	D	-	-	-	-	S	I	E	Y	M
GbHIV	133	14067.81	S	D	V	Q	L	Q	E	N	G	G	-	G	C	V	K	A	G	G	S	L	R	L	S	C	A	A	S	G	S	I	S	-	-	-	-	R	F	N	A	M
GbRBD2	128	14139.79	S	E	V	Q	L	V	E	N	G	G	-	G	C	V	K	T	G	G	S	L	R	L	S	C	A	L	S	G	Y	T	F	-	-	-	-	S	I	F	P	T
GbRBD3	126	14037.5	S	H	V	Q	L	V	E	N	G	G	-	G	C	V	K	A	G	G	S	L	R	L	S	C	A	T	S	G	R	T	F	-	-	-	-	S	T	Y	R	M
GbMBP	125	13776.48	S	Q	V	Q	L	V	E	N	G	G	-	G	C	V	K	A	G	G	S	L	R	L	S	C	V	A	S	G	D	I	K	-	-	-	-	Y	I	S	Y	L

Gembody	IMGT numbering scheme																																															
	40	41	42	43	44	45	46	47	48	49	50	51	52	53	54	55	56	57	58	59	60	61A	61	62	63	64	65	66	67	68	69	70	71	72	73	74	75	76	77	78	79	80	81	82	83	84	85	86	87
Gb5-006	T	W	Y	R	Q	A	P	G	K	E	R	E	W	V	A	A	I	T	S	G	-	-	-	-	G	S	T	N	Y	A	D	S	V	K	-	G	R	F	T	I	S	R	D	N	A	E	N	T	V
GbD12	A	W	F	R	Q	A	P	G	K	E	R	E	F	V	A	A	V	T	S	T	-	-	-	G	A	G	T	H	Y	V	D	S	V	K	-	G	R	F	T	I	S	R	V	N	A	E	N	T	M
GbS2A4	G	W	Y	R	R	A	P	G	K	E	R	E	L	V	A	G	I	T	L	G	-	-	-	-	G	T	T	Y	Y	A	D	S	V	K	-	G	R	F	T	I	S	R	D	N	A	K	N	T	V
GbEnhancer	R	W	Y	R	Q	A	P	G	K	E	R	E	W	V	A	G	M	S	S	A	-	-	-	G	D	R	S	S	Y	E	D	S	V	K	-	G	R	F	T	I	S	R	D	D	A	R	N	T	V
GbH12	A	W	Y	R	Q	A	P	R	K	Q	R	E	L	V	A	R	I	P	M	G	-	-	-	-	G	R	P	M	Y	A	D	S	V	K	-	G	R	F	T	I	S	R	D	N	A	E	N	T	V
GbLysozyme	T	W	F	R	Q	A	P	G	K	A	R	E	G	V	A	A	L	Y	T	H	-	-	-	T	G	N	T	Y	Y	T	D	S	V	K	-	G	R	F	T	I	S	Q	D	K	A	K	N	M	A
GbHIV	G	W	W	R	Q	A	P	G	K	E	R	E	F	V	A	R	I	V	K	G	-	-	-	-	F	D	P	V	L	A	D	S	V	K	-	G	R	F	T	I	S	I	D	S	A	E	N	T	L
GbRBD2	A	W	F	R	Q	A	P	G	K	E	R	E	F	V	A	G	I	R	W	N	G	S	T	R	D	Y	T	E	Y	A	D	F	V	K	-	G	R	F	T	I	S	R	D	N	A	E	N	M	V
GbRBD3	S	W	F	R	Q	A	P	G	K	E	R	E	F	V	A	T	I	I	W	S	-	-	-	V	G	S	T	H	Y	A	D	S	V	K	-	G	R	F	T	I	S	R	D	N	A	E	N	M	V
GbMBP	G	W	F	R	Q	A	P	G	K	E	R	E	G	V	A	A	L	Y	T	S	-	-	-	T	G	R	T	Y	Y	A	D	S	V	K	-	G	R	F	T	V	S	L	D	N	A	K	N	T	V

Gembody	IMGT numbering scheme																																																	
	88	89	90	91	92	93	94	95	96	97	98	99	100	101	102	103	104	105	106	107	108	109	110	111	111A	111B	111C	111D	112D	112C	112B	112A	112	113	114	115	116	117	118	119	120	121	122	123	124	125	126	127	128
Gb5-006	Y	L	Q	M	N	S	L	K	P	E	D	T	A	V	Y	Y	C	E	A	Y	G	T	Y	T	L	A	P	-	-	T	G	E	G	E	Y	D	D	Y	W	G	Q	G	T	Q	V	M	V	S	*
GbD12	Y	L	Q	M	N	S	L	K	P	E	D	T	A	V	Y	Y	C	A	A	A	N	T	R	L	T	A	L	S	L	R	T	T	T	G	S	W	A	Y	W	G	K	G	T	Q	V	M	V	S	*
GbS2A4	Y	L	Q	M	N	S	L	K	P	E	D	T	A	V	Y	Y	C	A	A	W	V	E	Y	-	-	-	-	-	-	-	-	-	P	R	R	Y	V	Y	W	G	Q	G	T	Q	V	M	V	S	*
GbEnhancer	Y	L	Q	M	N	S	L	K	P	E	D	T	A	V	Y	Y	C	N	V	N	V	-	-	-	-	-	-	-	-	-	-	-	-	-	G	F	E	Y	W	G	Q	G	T	Q	V	M	V	*	-
GbH12	Y	L	Q	M	N	S	L	K	P	D	D	T	A	V	Y	Y	C	N	A	V	T	Y	-	-	-	-	-	-	-	-	-	-	-	G	L	E	S	Y	W	G	K	G	T	Q	V	M	V	S	*
GbLysozyme	Y	L	R	M	D	S	V	K	S	E	D	T	A	I	Y	T	C	G	A	T	R	K	Y	V	P	V	R	-	F	A	L	D	Q	S	S	Y	D	Y	W	G	Q	G	T	Q	V	M	V	S	SSSAG
GbHIV	A	L	Q	M	N	R	L	K	P	E	D	T	A	V	Y	Y	C	F	A	A	L	-	-	-	-	-	-	-	-	-	-	-	-	-	D	T	A	Y	W	G	Q	G	T	Q	V	M	V	S	SSA...
GbRBD2	Y	L	Q	M	I	S	L	K	P	E	D	T	A	L	Y	Y	C	A	A	S	D	G	V	I	D	G	-	-	-	-	T	N	A	N	A	Y	R	Y	W	G	Q	G	T	Q	V	M	V	S	S
GbRBD3	Y	L	Q	M	N	S	L	K	P	E	D	T	A	V	Y	Y	C	A	A	Q	R	S	D	S	S	S	-	-	-	W	G	Y	E	D	D	Y	D	Y	W	G	Q	G	T	Q	V	M	V	S	S
GbMBP	Y	L	Q	M	N	S	L	K	P	E	D	T	A	L	Y	Y	C	A	A	A	E	W	G	S	Q	S	-	-	-	P	L	T	Q	W	F	Y	R	Y	W	G	Q	G	T	Q	V	M	V	S	-

b

Gembody	No. of Amino	Theoretical Molecular	N-terminal head	IMGT numbering scheme																																						
				1	2	3	4	5	6	7	8	9	10	11	12	13	14	15	16	17	18	19	20	21	22	23	24	25	26	27	28	29	30	31	32	33	34	35	36	37	38	39
GbC4	122	13268.9	S	Q	G	Q	L	V	E	N	G	G	-	G	C	V	K	A	G	G	S	L	R	L	S	C	A	A	S	Q	G	T	L	-	-	-	-	S	N	L	V	T
Gb113	118	12676.14	S	Q	V	Q	L	Q	E	N	G	G	-	G	C	V	K	P	G	G	S	L	R	L	S	C	A	A	S	G	F	T	F	-	-	-	-	S	S	Y	Y	M
GbRBD1	127	14072.81	S	Q	V	Q	L	V	E	N	G	G	-	G	C	M	K	A	G	G	S	L	R	L	S	C	A	V	S	G	R	T	F	-	-	-	-	S	T	A	A	M
GbRBD6	125	14038.69	S	Q	V	Q	L	V	E	N	G	G	-	G	C	V	K	A	G	G	S	L	R	L	A	C	I	A	S	G	R	T	F	-	-	-	-	H	S	Y	V	M

Gembody	IMGT numbering scheme																																														
	40	41	42	43	44	45	46	47	48	49	50	51	52	53	54	55	56	57	58	59	60	61	62	63	64	65	66	67	68	69	70	71	72	73	74	75	76	77	78	79	80	81	82	83	84	85	86	87
GbC4	G	W	F	R	R	A	P	G	K	E	R	E	F	V	A	N	I	G	R	D	-	-	-	G	L	T	V	Y	S	N	S	V	K	-	G	R	F	T	I	S	R	D	R	A	K	N	T	V
Gb113	S	W	V	R	Q	A	P	G	K	G	P	E	W	V	S	G	I	N	T	G	-	-	G	V	G	T	R	Y	A	D	S	V	K	-	G	R	F	T	I	S	R	D	N	A	K	N	T	L
GbRBD1	G	W	F	R	Q	A	P	G	K	E	R	E	F	V	A	A	I	R	W	S	-	-	G	G	S	A	Y	Y	A	D	S	V	K	-	G	R	F	T	I	S	R	D	K	A	E	N	T	V
GbRBD6	A	W	F	R	Q	A	P	G	K	E	R	E	F	V	A	A	I	S	W	S	-	-	S	T	P	T	Y	Y	G	E	S	V	K	-	G	R	F	T	I	S	R	D	N	A	E	N	T	V

Gembody	IMGT numbering scheme																																															
	88	89	90	91	92	93	94	95	96	97	98	99	100	101	102	103	104	105	106	107	108	109	110	111	111A	111B	111C	112D	112C	112B	112A	112	113	114	115	116	117	118	119	120	121	122	123	124	125	126	127	128
GbC4	Y	L	Q	M	D	S	L	K	P	E	D	T	A	V	Y	Y	C	A	G	R	L	S	R	F	-	-	-	-	-	-	-	P	G	E	Y	D	Y	W	S	K	G	T	P	V	M	V	S	SSQ
Gb113	Y	L	Q	M	N	S	L	K	P	E	D	T	A	L	Y	Y	C	A	I	G	E	G	-	-	-	-	-	-	-	-	-	-	G	N	R	N	Y	W	G	Q	G	T	Q	V	M	V	S	S
GbRBD1	Y	L	Q	M	N	S	L	K	Y	E	D	T	A	V	Y	Y	C	A	Q	T	R	V	T	R	S	L	L	S	D	Y	A	T	W	P	Y	D	Y	W	G	Q	G	T	Q	V	M	V	S	*
GbRBD6	Y	L	Q	M	N	R	L	K	P	E	D	T	A	V	Y	F	C	A	A	D	R	G	E	S	Y	Y	-	-	Y	T	R	P	T	E	Y	E	F	W	G	Q	G	T	Q	V	M	V	S	*

Supplementary Fig. 5 Gembody sequences aligned in the IMGT scheme. (a) Sequence information of 10 Gembodies used in homoDiGbs. (b) Sequence information of 3 additional Gembodies used in heteroDiGbs. CDRs of the Gembodies are highlighted in cyan. The origins are as follows: alpaca immunization: GbD12, GbH12, GbC4, Gb11; llama immunization: RBD1, RBD3, RBD6, Gb5-006, GbHIV; camelid: GFPenhancer; dromedary: GbLysozyme; synthetic nanobody: GbMBP (Zimmermann library), GbS2A4 (McMahon library).

Supplementary Fig. 6

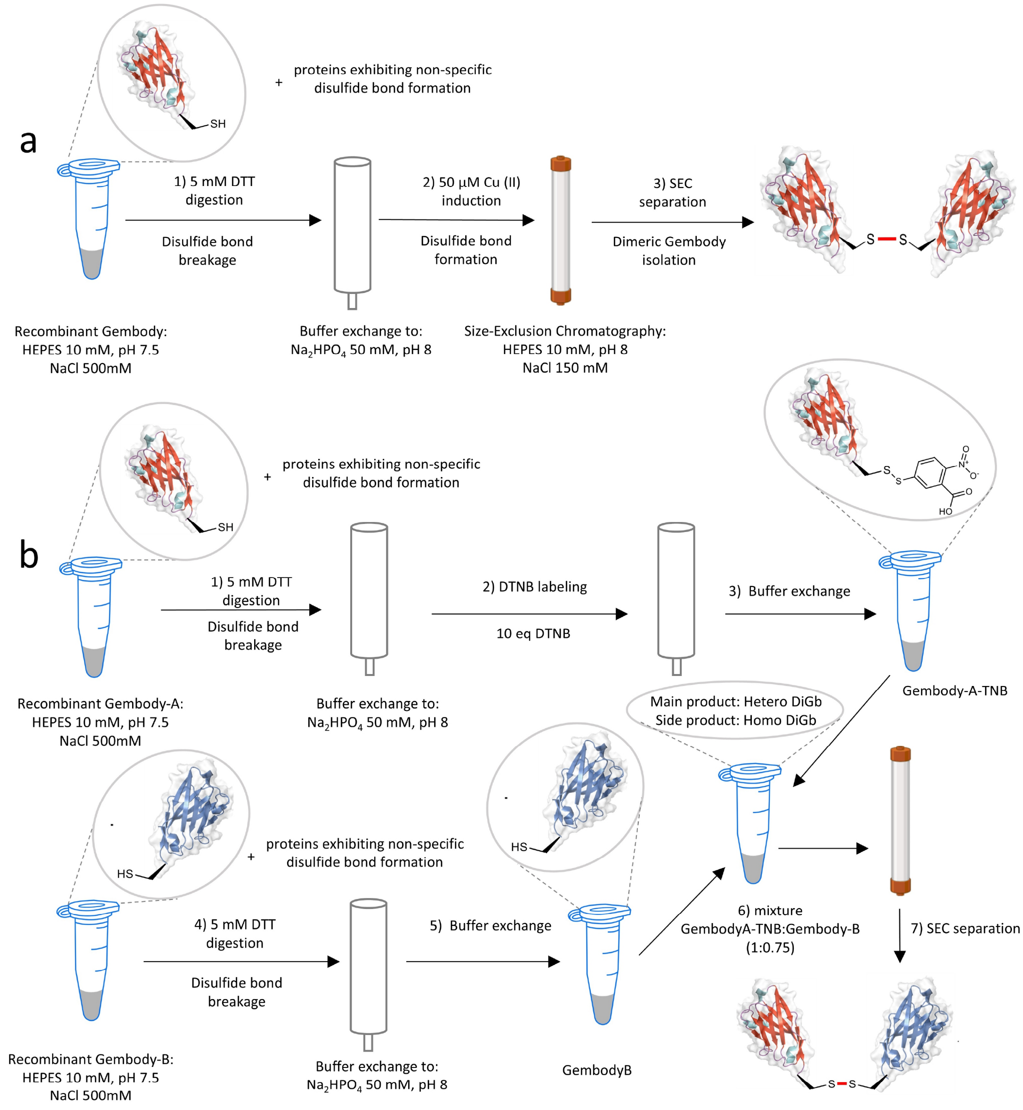

Supplementary Fig. 6 Synthesis notes of homo Di-Gembodies (a) and hetero Di-Gembodies (b) Biorender licence for the SEC column item is XM289VST7B.

Supplementary Fig. 7

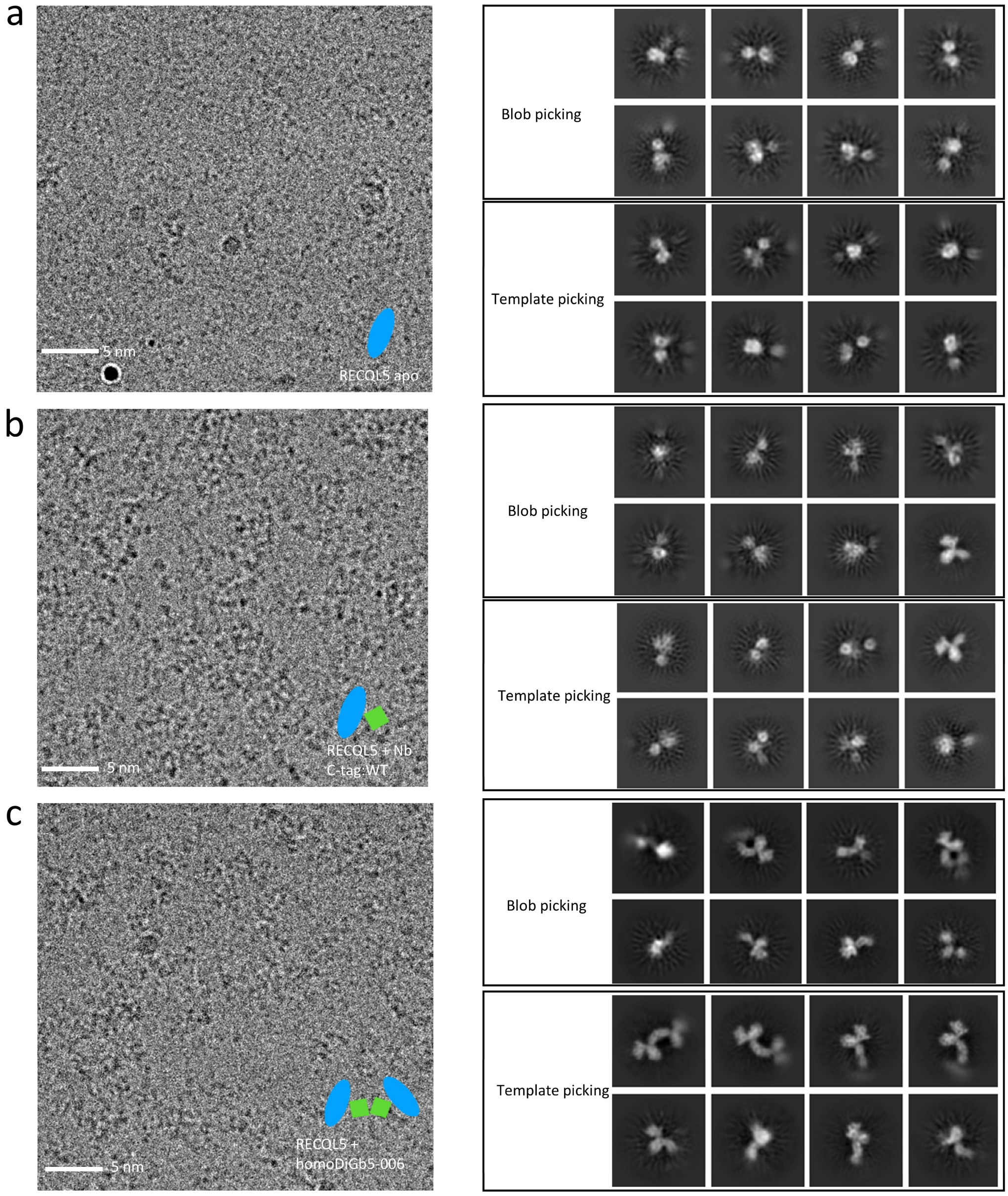

Supplementary Fig. 7 The homo Di-Gembody improves the alignment of RECQL5. Cryo-EM data and 2D classification results for (a) RECQL5 alone, (b) RECQL5 in complex with nanobody, and (c) RECQL5 complexed with homo Di-Gembody Gb5-006 with Data collected from 200 kV Glacios microscope. Exemplar raw images, schematics, and 2D classification of particles picked using blob and template-based picking are shown for each sample. Similar images were captured on three separate occasions for all above samples.

Supplementary Fig. 8

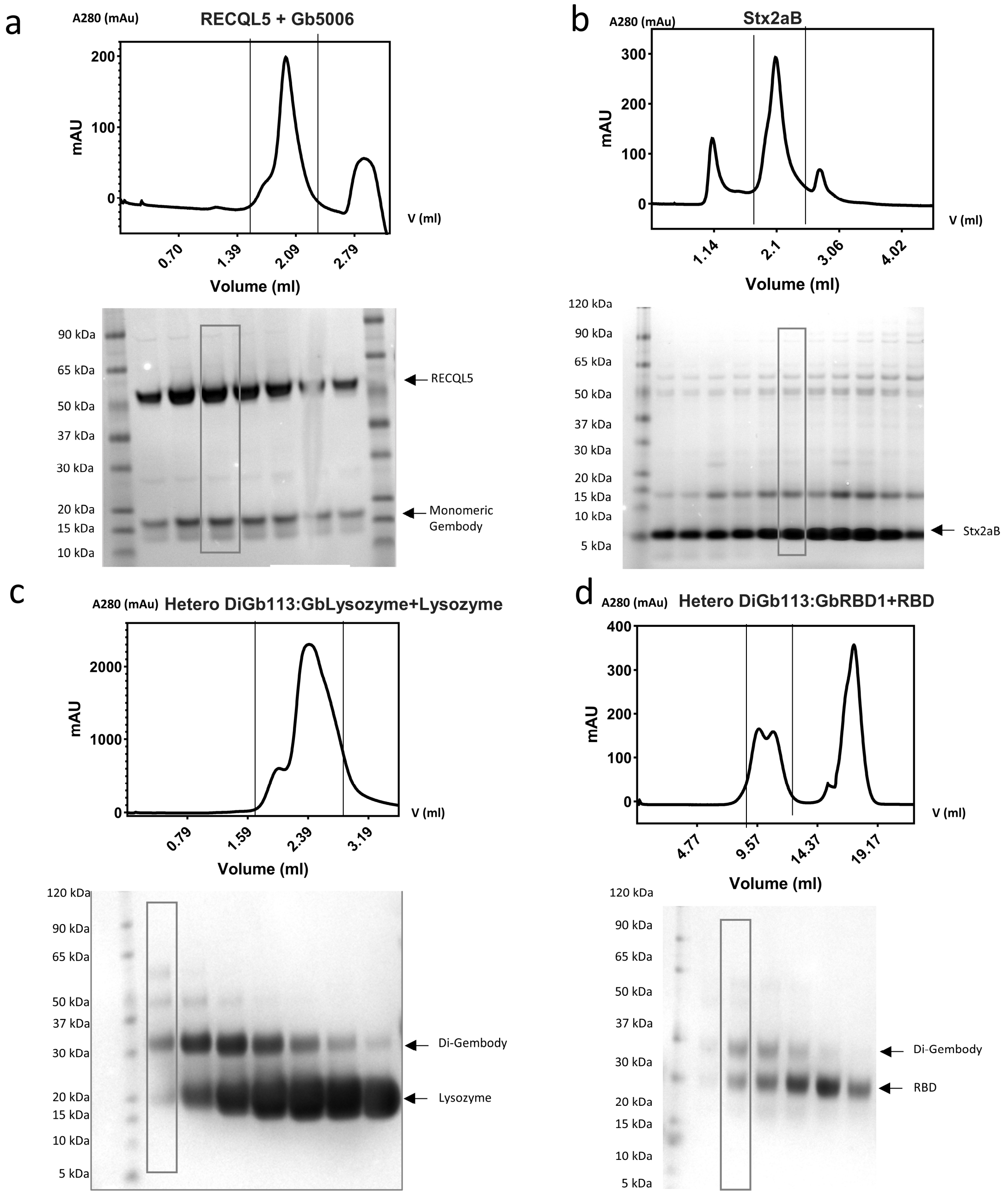

Supplementary Fig. 8 Pentameric complex reconstitutions with size exclusion chromatography (SEC). (a) Purification of RECQL5 in complex with the Nb5-006. (b) Purification of Stx2aB. (c) Purification of Lysozyme in complex with hetero DiGb113:GbLysozyme. (d) Purification of RBD in complex with hetero DiGb113:GbRBD1. The trials above performed twice with similar results. See Supplementary Fig. 18 for uncropped gels.

Supplementary Fig. 9

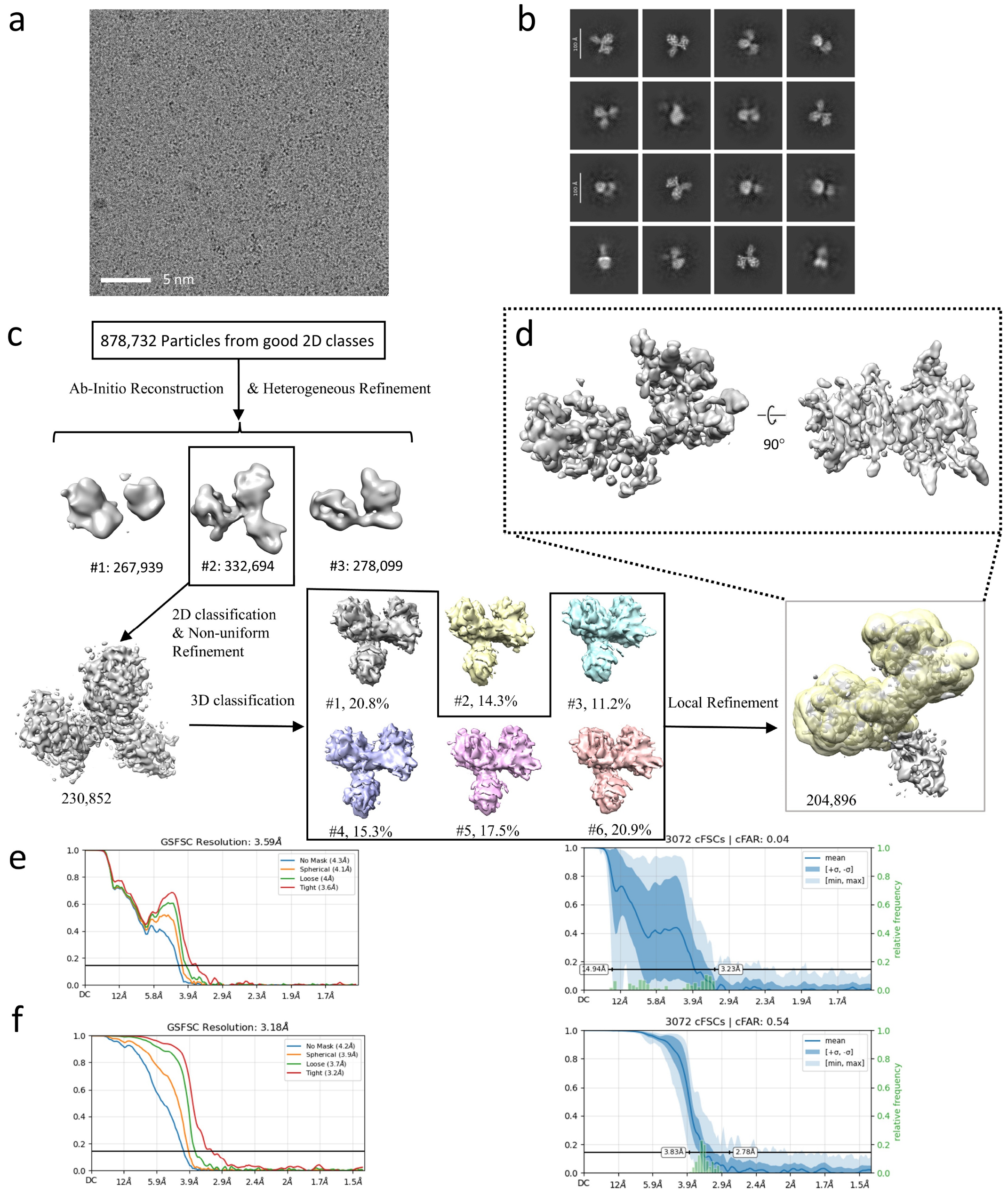

Supplementary Fig. 9 Comparative Cryo-EM structural determination of RECQL5 in complex with Gb5-006. (a) Exemplar raw micrographs. Similar images were captured on three separate occasions. (b) 2D classification results of initial particles after Topaz picking. (c) Cryo-EM 3D reconstruction pipeline. (d) Overall structure of RecQL5 in views related to a 90 degree orientation. (e) The Fourier Shell Correlation curves and orientation distribution of the RECQL5 (generated from RECQL5:Nb5-006) after local refinement (left) and local resolution distribution (right). (f) The Fourier Shell Correlation curves and orientation distribution of the RECQL5 (generated from RECQL5:homoDiGb5006) after local refinement (left) and local resolution distribution (right).

Supplementary Fig. 10

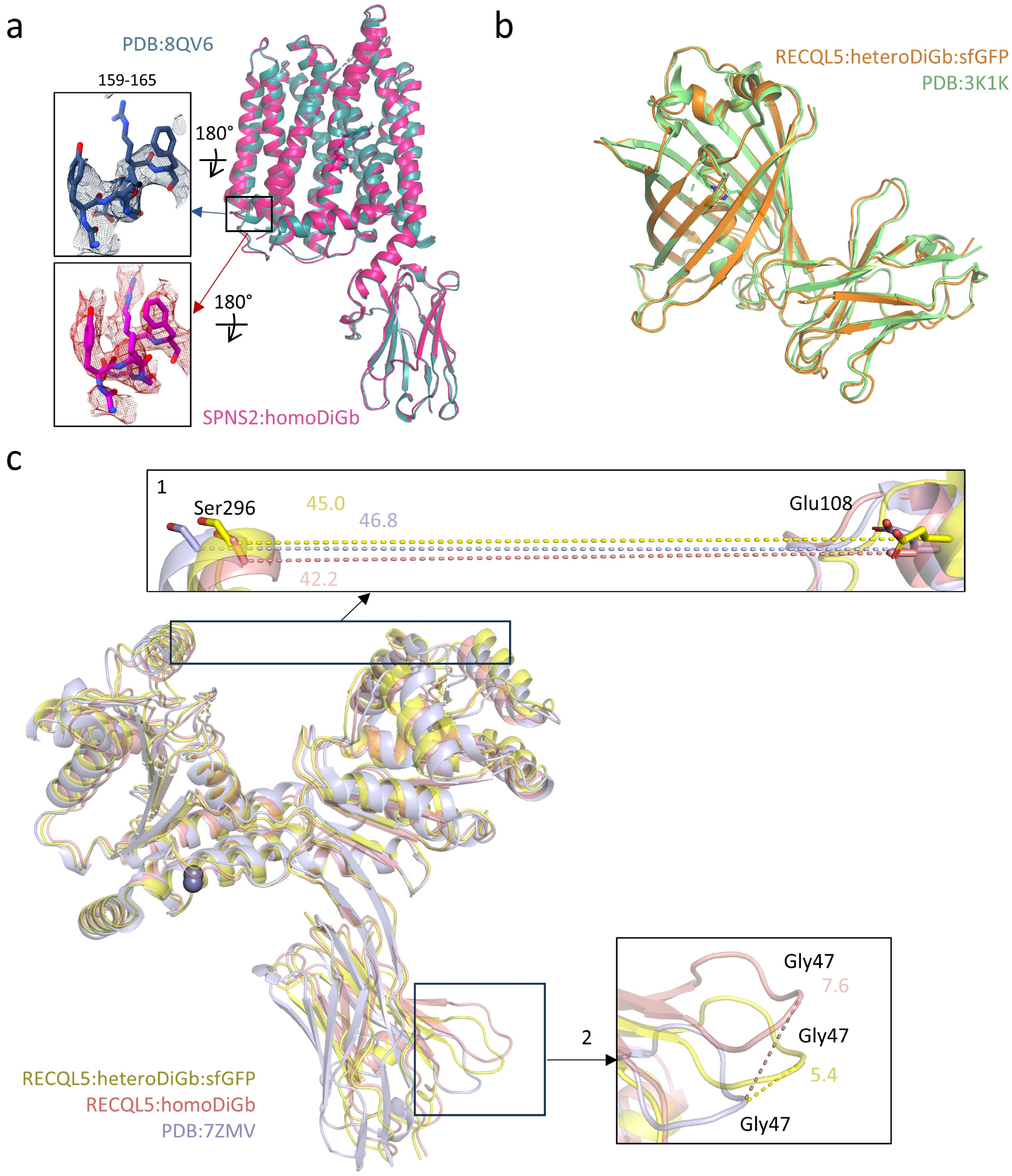

Supplementary Fig. 10 Comparison of the solved structures using Di-Gembodies and previously determined structures. (a) Structural 3D alignment of the sfGFP:GbEnhancer complex in the RECQL5:sfGFP:hetDiGb(Gb5-006:GbEnhancer) complex structure and the published non-Gembody structure (3K1K). (b) Structural 3D alignment of the SPNS2:GbD12 complex in the SPNS2:homoDiGb structure and the published SPSNS2:NbD12 structure (8QV6) with inset showing sidechain density improvements. (c) Structural 3D alignment of the RECQL5:Gb5-006 complex in the RECQL5:homoDiGb complex and RECQL5:heteroDiGb:sfGFP complex structures and the published RECQL5:Gb5-006 structure (7ZMV). Inset 1 distance between Cα atoms of RECQL5's Glu108 in D1 domain and Ser296 in D2 domain. Inset 2 shows the distance comparison between Cα atoms of Gly47 of the Gembodies. Distances are shown in Å.

Supplementary Fig. 11

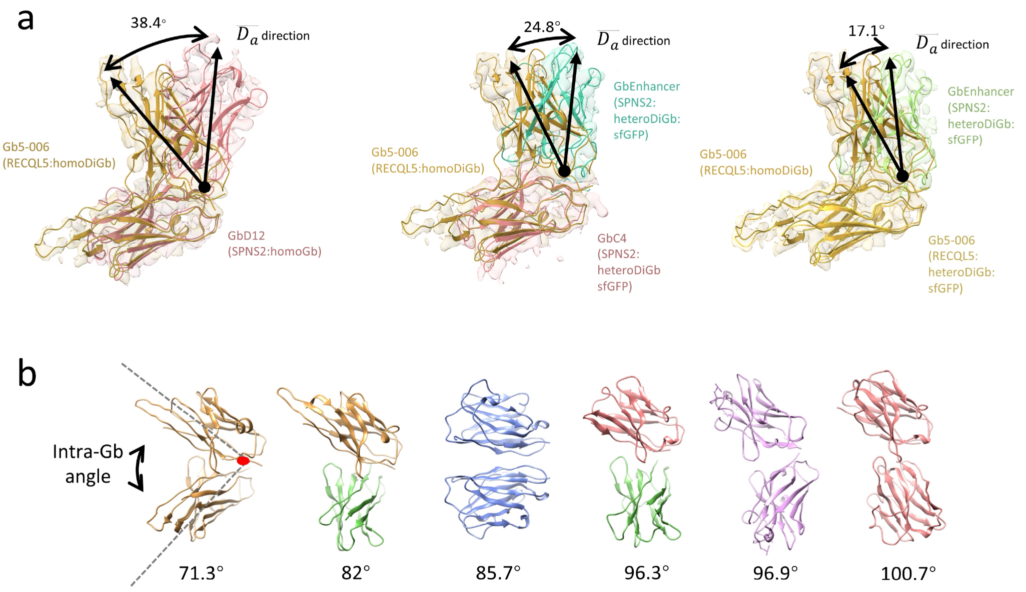

Supplementary Fig. 11 Structural comparisons of homo or hetero DiGb. (a) Structural comparisons of homoDiGbD12, heteroDiGbs GbC4:GbEnhancer and Gb5-006:GbEnhancer with homoDiGb5-006. Intra-DiGb angles are indicated and $\overrightarrow{D_a}$ direction is defined in the methods section (b) Different DiGb pairs have unique intra-DiGb angles. The intra-DiGb angle is defined by the angle of the vectors from the Cα atom of Cys12 to the mass centre of respective Gembodies. The represented structures from left to right are: RECQL5:homoDiGb, RECQL5:heteroDiGb:sfGFP, MBP:homoDiGb, SPNS2:heteroDiGb:sfGFP, Lysozyme:homoDiGb, SPNS2:homoDiGb.

Supplementary Fig. 12

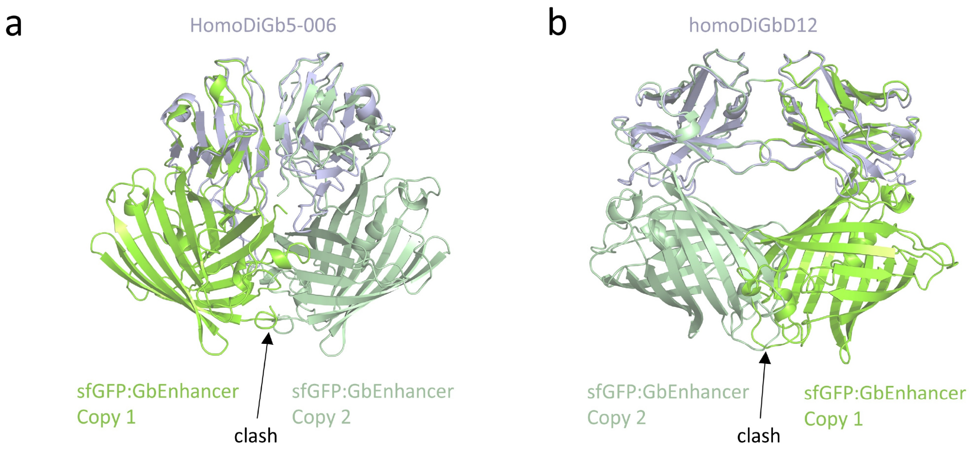

Supplementary Fig. 12 The GbEnhancer homo Di-Gembody is expected to introduce clash when the target GFP is attached. (a) The GFP:NbEnhancer structure (PDB: 3K1K) modeled on the homo Di-Gembody Gb5-006. (b) The 3K1K structure modeled on the homo Di-Gembody GbD12. Clash is indicated by the arrow.

Supplementary Fig. 13

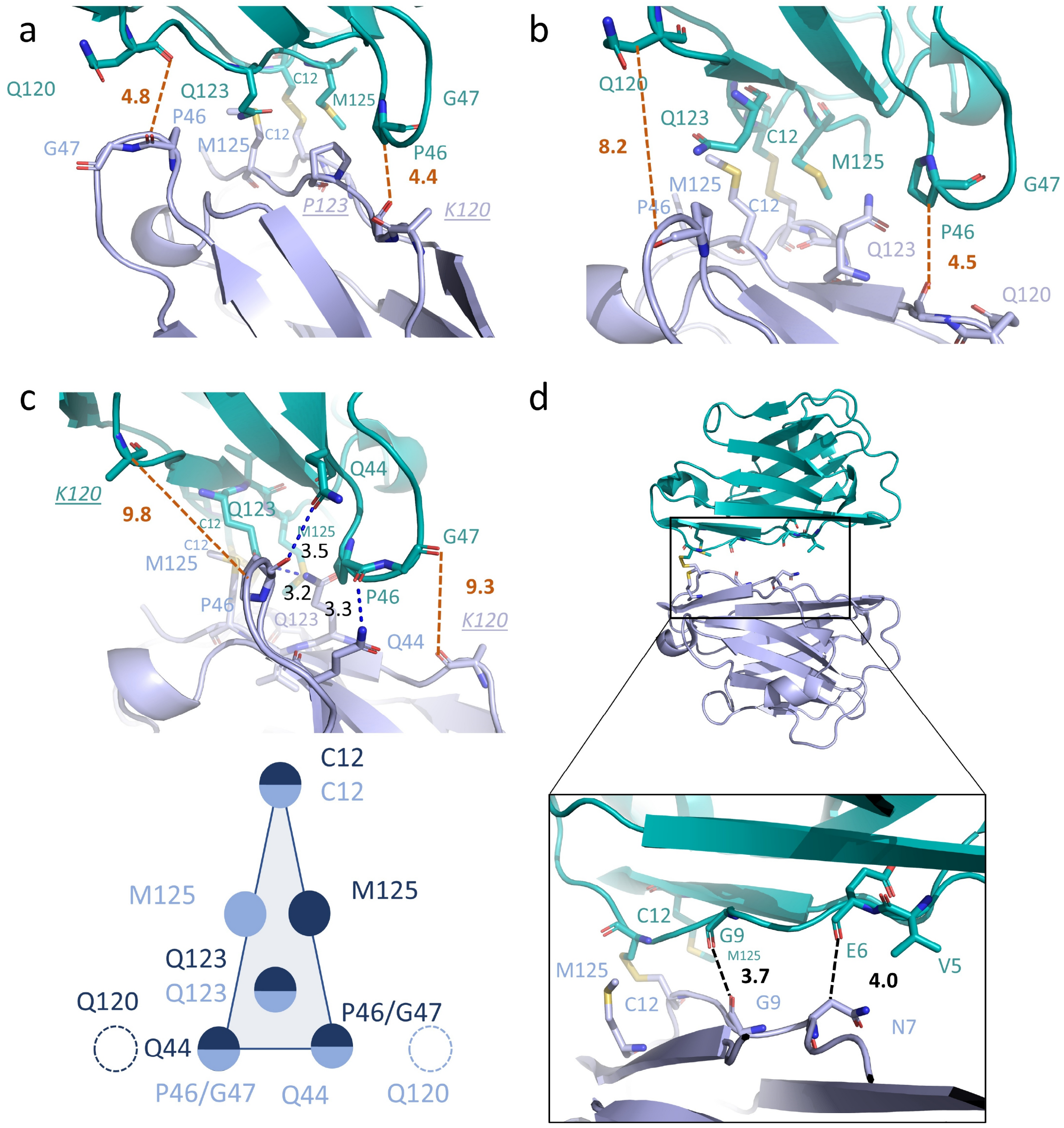

Supplementary Fig. 13 The DiGb interfaces are influenced by the interacting residue side chains and driven by the disulfide. Two Gembodies are shown in lightblue and teal. Typical Cys-Cys interfaces were present in structures of (a) SPNS2:heteroDiGb:sfGFP and (b) Lysozyme:homoDiGb. Narrower Cys-Cys interface was present in the structure of (c) SPNS2:homoDiGb with P46/G47 interacting with Q44 instead of residue 120, which has a Lysine instead of Glutamine at this position. Interface cartoon is drawn below. The MBP:homoDiGb presents an even more unique DiGb interface that is chemically driven by the disulfide and assisted by the first beta-sheets of the Gembodies. Blue dashes show hydrogen bonds, and dashes show the minimum distances (not counting hydrogen atoms) between the P46/G47 and Q/K120 pairs, and black ones show distances between two atoms. Atypical residue types are shown in italics underlined.

Supplementary Fig. 14

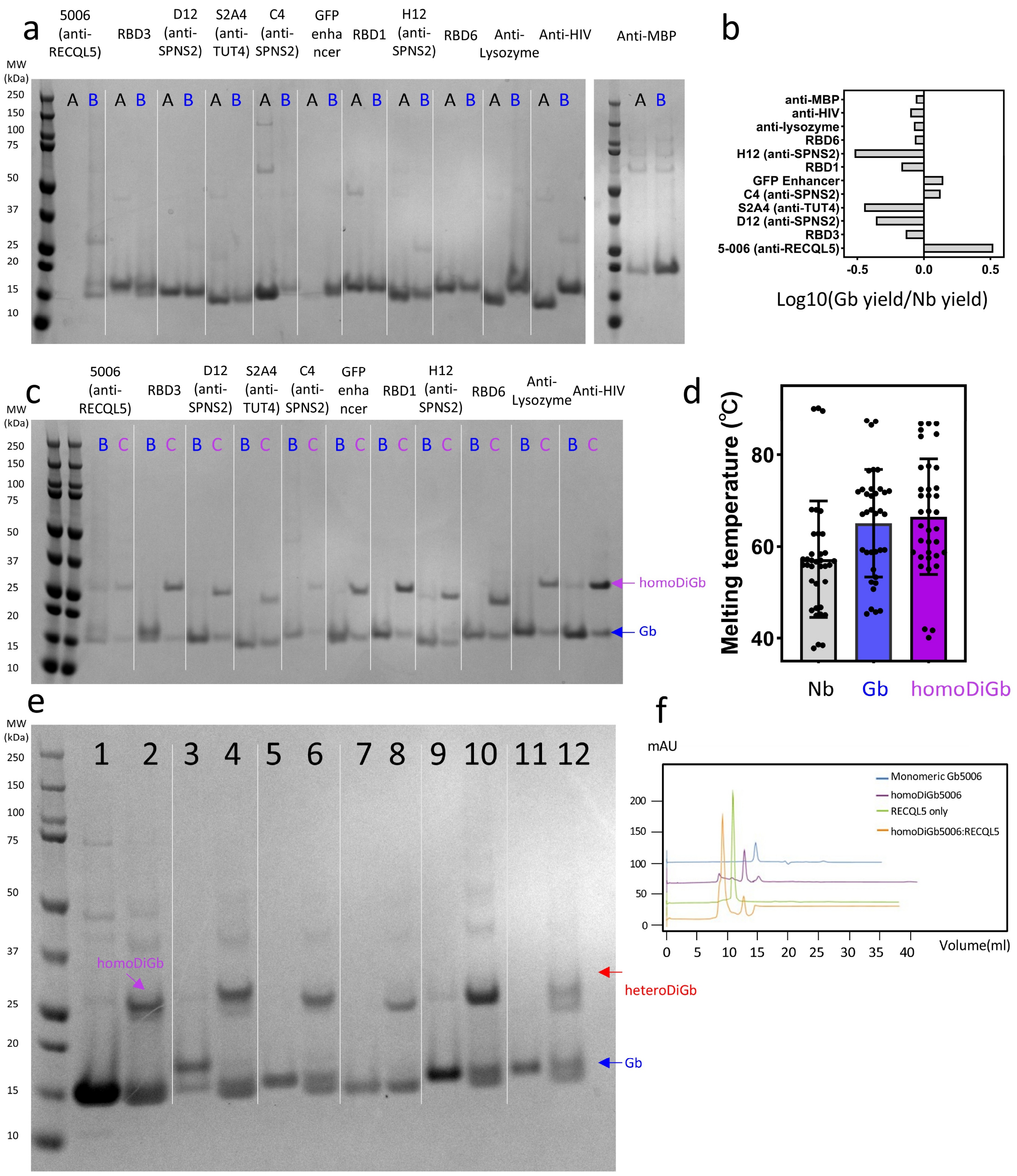

Supplementary Fig. 14 Biochemical characterization of Gembodies including SDS-PAGE, yield, thermal stability and SEC profiles. a) SDS-PAGE analysis of wildtype nanobody (black 'A's on the gel) and monomeric Gembody (blue 'B's on the gel). Different CDR groups are separated by white lines on the gel with names annotated above. The trials above were conducted twice with similar results. b) Yield comparison between Gembodies and nanobodies for each CDR group. Yields for both Gembodies and nanobodies were initially obtained by dividing the protein quantity (in mg) obtained after reverse IMAC with the wet cell pellet weight of the bacteria (in grams), and the bar shows the value of log10(Gb yield/Nb yield). c) SDS-PAGE analysis of monomeric Gembody (blue 'B's on the gel) and homo Di-Gembodies (purple 'C's on the gel). Different CDR groups are separated by white lines on the

gel with names annotated above. The trials above were conducted twice with similar results. d) thermal stability comparison between nanobodies, monomeric Gembodies and homo-DiGembodies. Two-tailed t-tests were performed between groups. '**':$p<0.01$; 'n.s.': not significant. P value Gb vs Nb: 0.0078, homoDiGb vs Nb is 0.0026, homoDiGb vs Gb is 0.6237 (n.s.) e) SDS-PAGE analysis of hetero-DiGembodies. 1, monomeric GbEnhancer; 2, Gbenhancer-TNB; 3, monomeric G5006; 4, heteroDiGb5006:GbEnhancer; 5, monomeric GbC4; 6, heteroDiGbGbC4:GbEnhancer; 7,monomeric GbH12; 8, heteroDiGbGbH12:GbEnhancer; 9, monomeric GbRBD1; 10, heteroDiGbGbRBD1:GbEnhancer; 11, monomeric GbRBD6; 12, heteroDiGbGbRBD6:GbEnhancer. The trials obovewere conducted twice with similar results f) Size exclusion chomatography using Superdex 75 increase 10/300GL to separate monomeric Nb5006, homoDiGb5006, RECQL5:Nb5006 and RECQL5:homoDiGb.

Supplementary Fig. 15

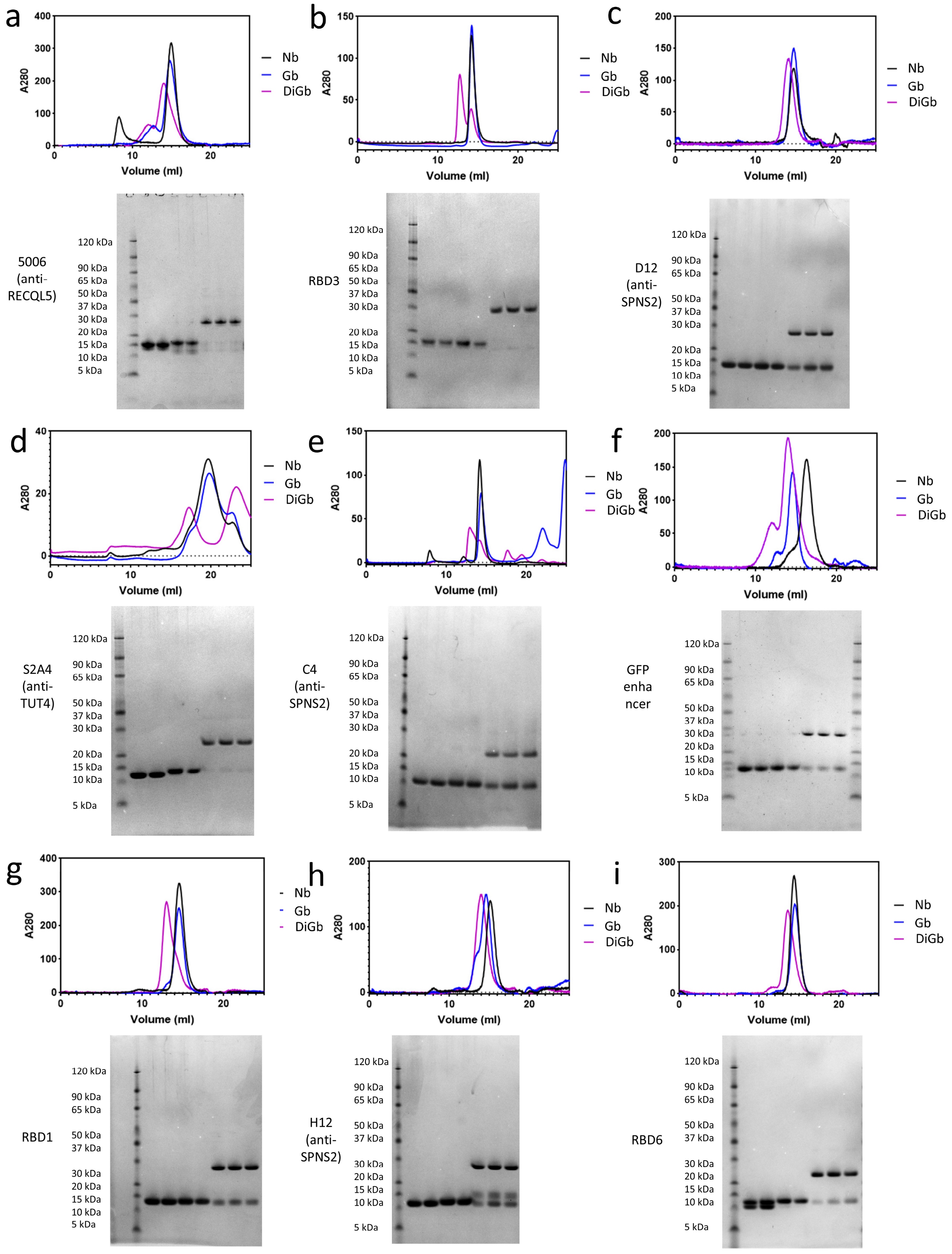

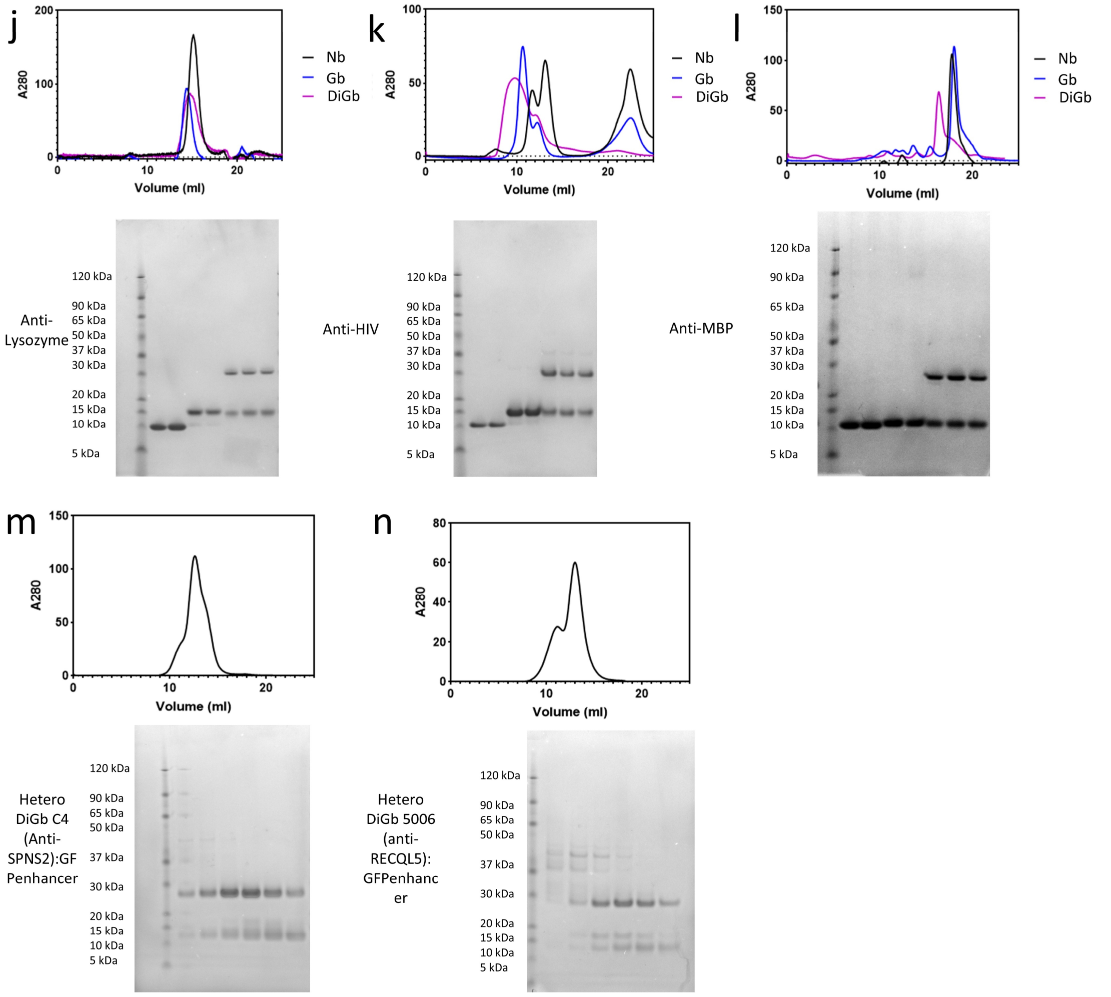

Supplementary Fig. 15 Comparative chromatographic and electrophoretic characterisation of wild-type nanobody (Nb), Gembody (Gb), and DiGembody (DiGb) samples. SEC profiles and SDS-PAGE analysis of Nb (lanes 2-3), Gb (lanes 4-5), and DiGb (lanes 6-8),for a) 5006 (anti-RECQL5),b)RBD3, c) D12 (anti-SPNS2), d) S2A4(anti-TUT4), e) C4 (anti-SPNS2), f) GFP enhancer, g) RBD1, h) H12 (anti-SPNS2), i) RBD6, j) Anti-Lysozyme, k) Anti-HIV, l) Anti-MBP, m) SEC profiles and SDS-PAGE analysis of Hetero DiGb C4 (Anti-SPNS2):GFPenhancer, n) SEC profiles and SDS-PAGE analysis of Hetero DiGb 5006 (anti-RecQL5):GFPenhancer. The trials above performed twice with similar results. See Supplementary Fig. 18 for uncropped gels.

Supplementary Fig. 16

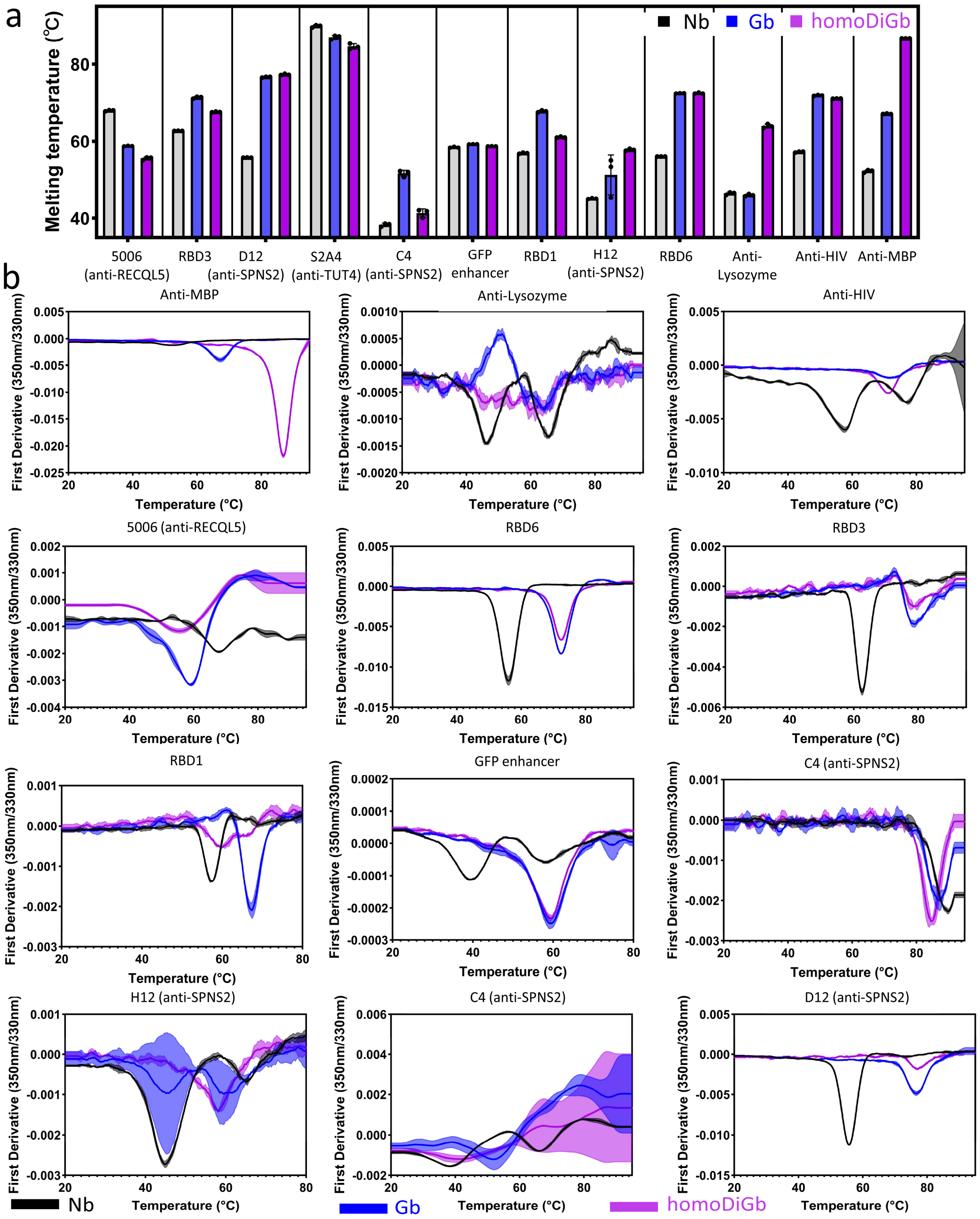

Supplementary Fig. 16 Differential scanning fluorimetry analysis of thermal stability of individual Nanobodies, Gembodies and homo-DiGembodies. a) barplots of melting temperatures of nanobodies, monomeric Gembodies and homo-DiGembodies, grouped by different CDR sets. Each nanoDSF measurement was conducted in technical triplicate. The bar heights indicate the mean values of the melting temperatures and the error bars indicate the standard deviation among triplicates. b) First derivative traces of 350nm/330nm ration from nanoDSF of nanobodies, monomeric Gembodies and homo-DiGembodies. The light shading represents the standard deviation among the technical triplicates. The light shading represents the standard deviation and the middle solid line within the shade indicates the

mean value The light shading represents the standard deviation among the technical triplicates.

Supplementary Fig. 17

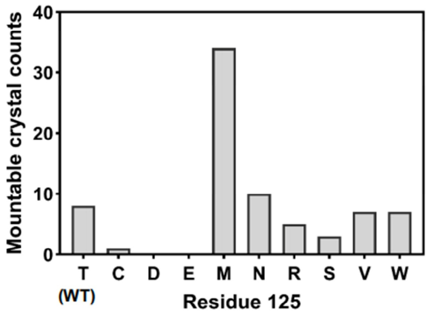

Supplementary Fig. 17 Mutational analysis at the Gb interface. Mountable crystal counts out of the Hampton Index 3 commercial screen for variants with mutations on residue 125**.**

Supplementary Fig. 18

a

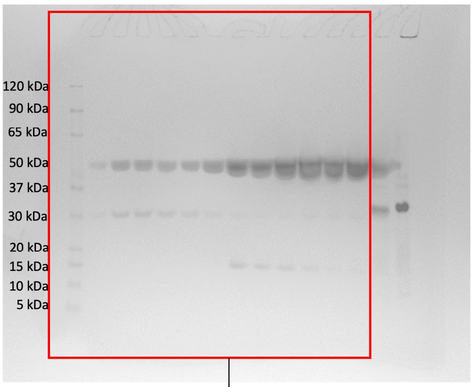

RECQL5 in complex with the homoDiGb5-006, marker in lane 1 (Red frame)

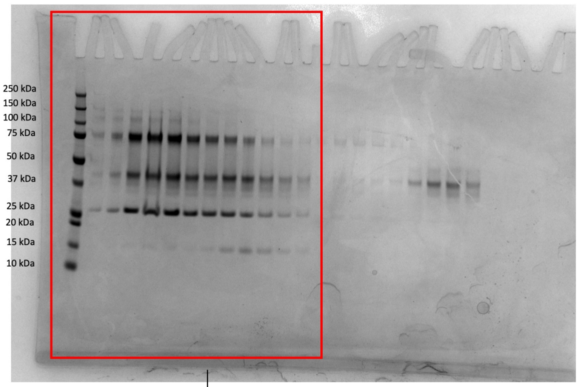

SPNS2 in complex with the homoDiGbD12, marker in lane 1 (Red frame)

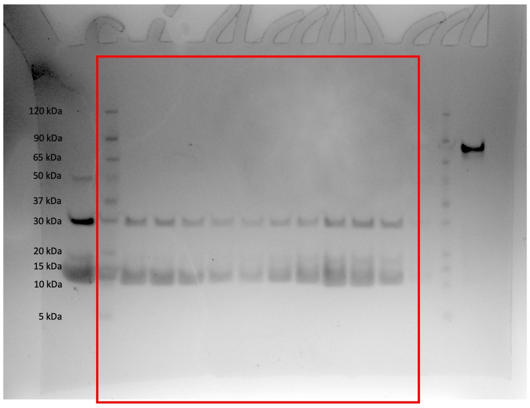

Rlysozyme in complex with the homoDiGbLysozyme, marker in lane 1 (Red frame)

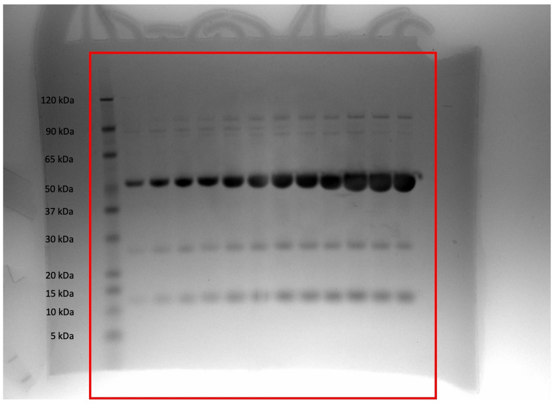

MBP in complex with the homoDiGb MBP, marker in lane 1 (Red frame)

b

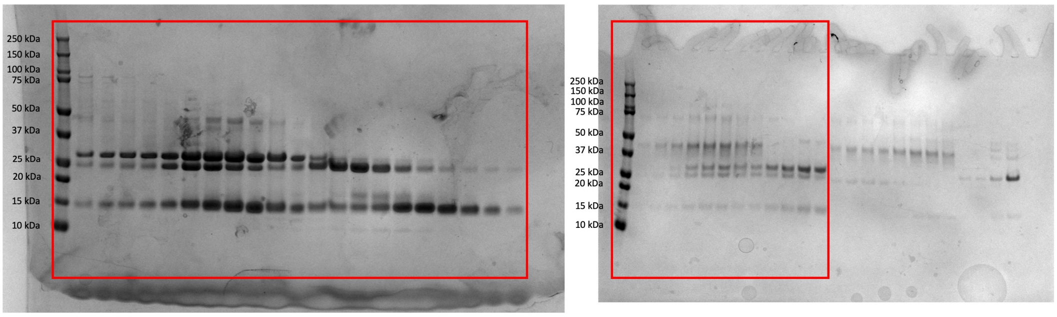

SPNS2:heteroDiGb:sfGFP complex,
marker in lane 1 (Red frame)

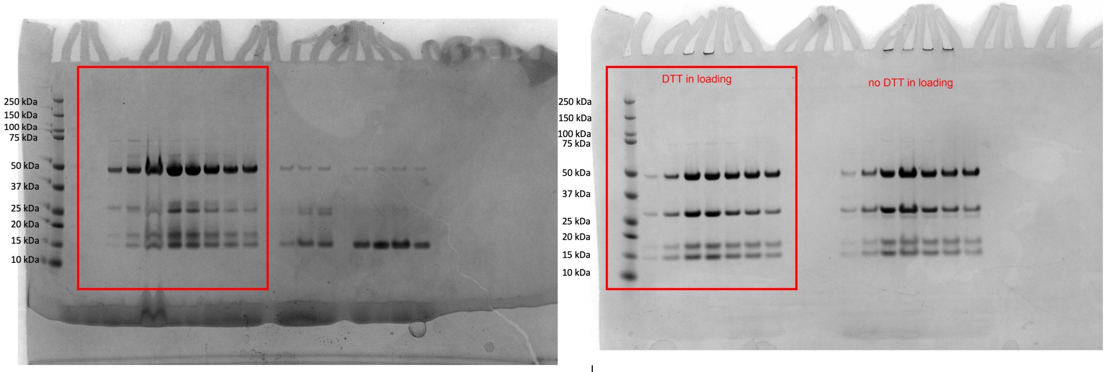

RECQL5:heteroDiGb:sfGFP complex,
marker in lane 1 (Red frame)

C

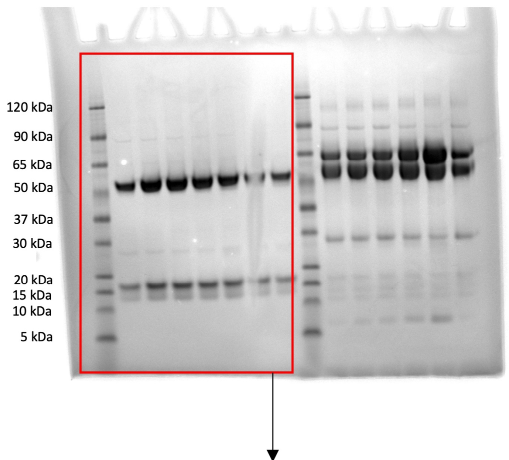

Purification of RECQL5 in complex with the Nb5-006, marker in lane 1 (Red frame)

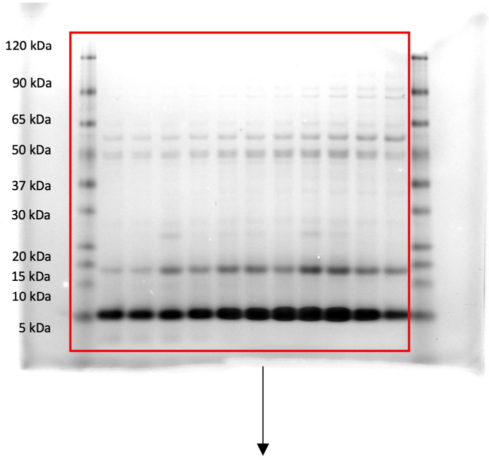

Stx2aB, marker in lane 1 (Red frame)

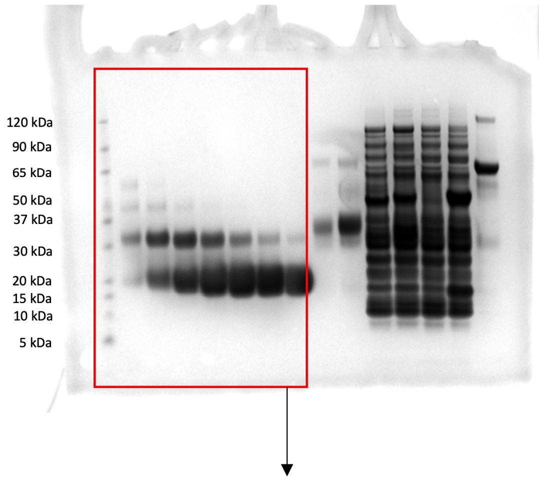

Lysozyme in complex with hetero DiGb113:GbLysozyme, marker in lane 1 (Red frame)

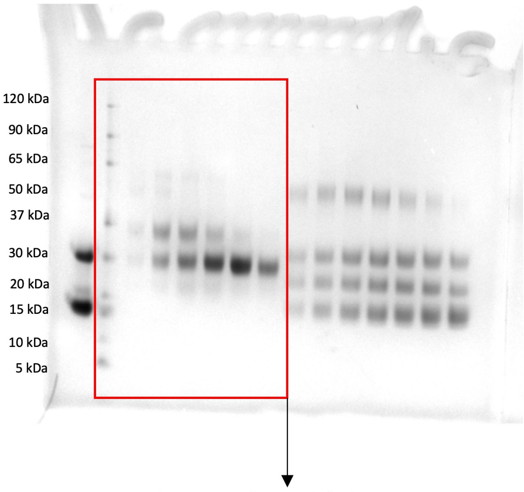

RBD in complex with hetero DiGb113:GbRBD1, marker in lane 1 (Red frame)

d

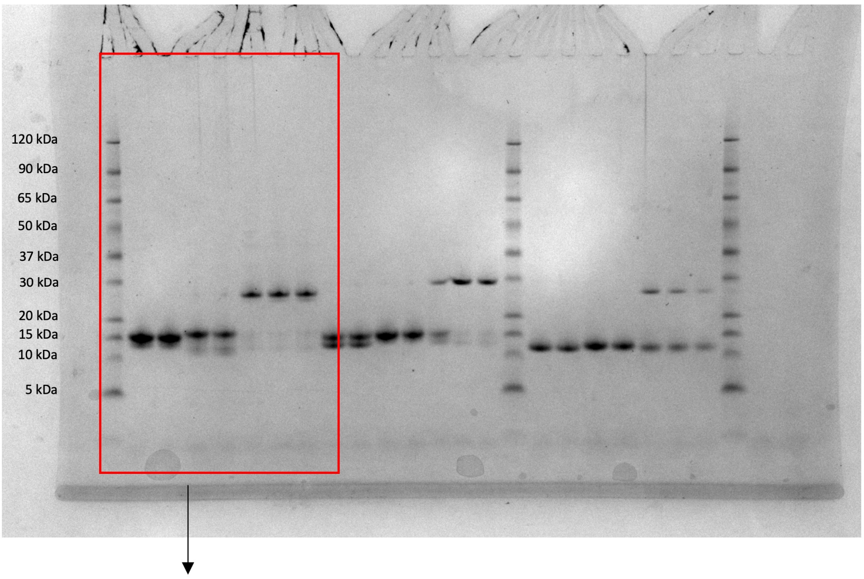

anti-RECQL5 Nb (lanes 2-3), Gb (lanes 4-5), and DiGb (lanes 6-8), marker in lane 1 (Red frame)

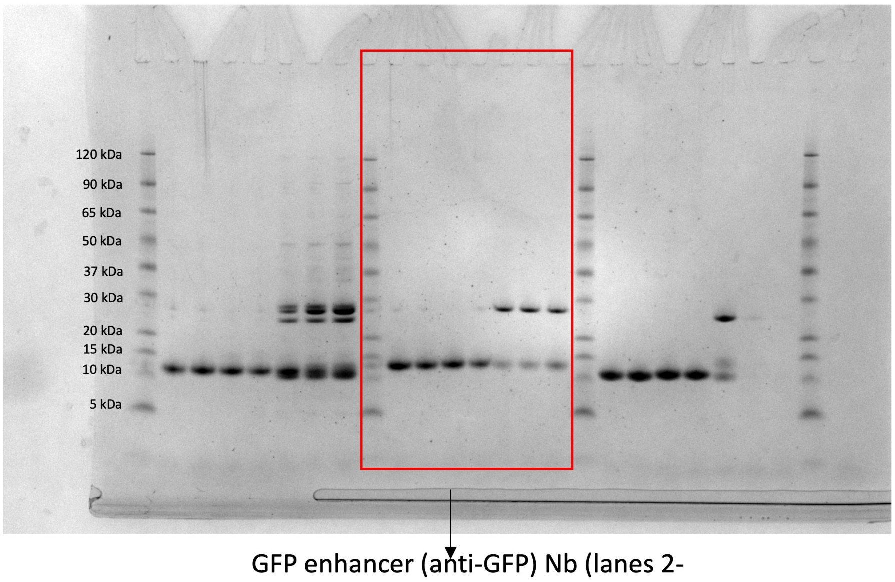

GFP enhancer (anti-GFP) Nb (lanes 2-3), Gb (lanes 4-5), and DiGb (lanes 6-8), marker in lane 1 (Red frame)

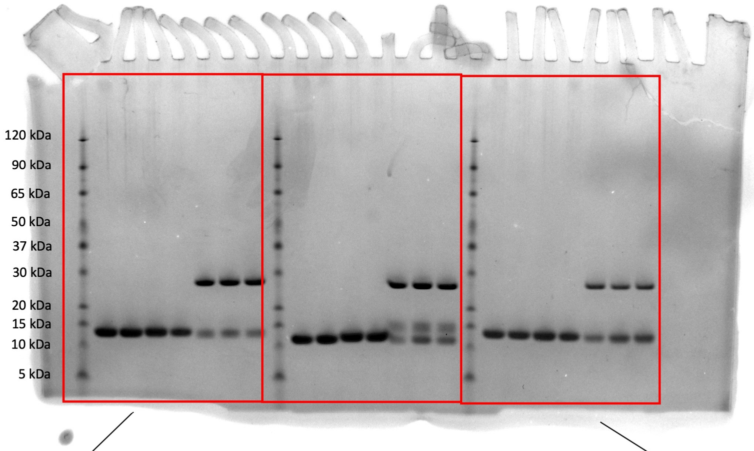

RBD1 (anti-RBD) Nb (lanes 2-3), Gb (lanes 4-5), and DiGb (lanes 6-8), marker in lane 1 (Red frame)

D12 (anti-SPNS2) Nb (lanes 2-3), Gb (lanes 4-5), and DiGb (lanes 6-8), marker in lane 1 (Red frame)

H12 (anti-SPNS2) Nb (lanes 2-3), Gb (lanes 4-5), and DiGb (lanes 6-8), marker in lane 1 (Red frame)

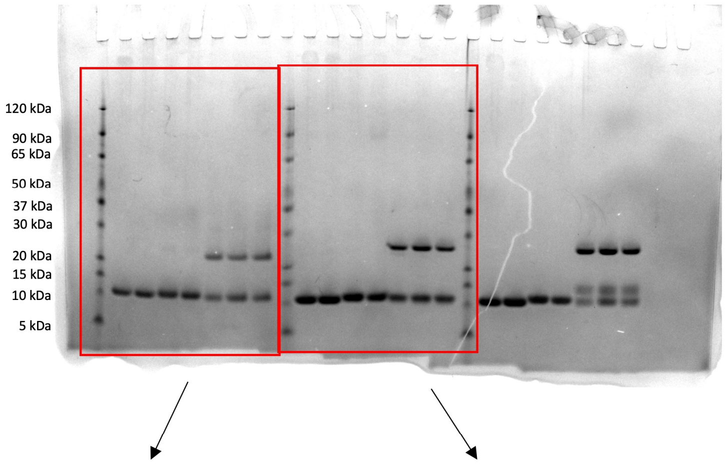

C4 (anti-SPNS2) Nb (lanes 2-3), Gb (lanes 4-5), and DiGb (lanes 6-8), marker in lane 1 (Red frame)

Anti-MBP Nb (lanes 2-3), Gb (lanes 4-5), and DiGb (lanes 6-8), marker in lane 1 (Red frame)

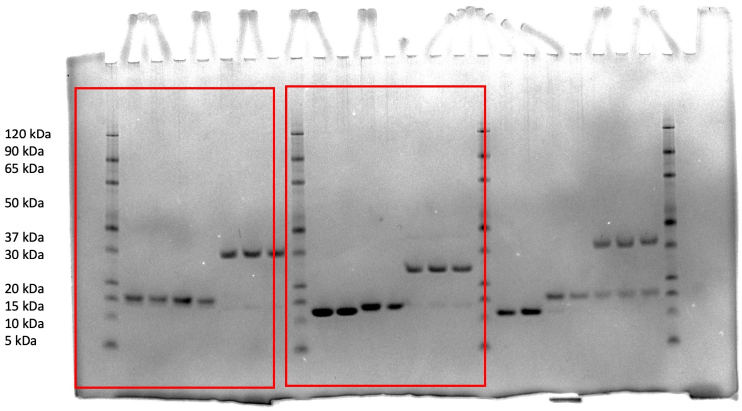

RBD3 (anti-RBD) Nb (lanes 2-3), Gb (lanes 4-5), and DiGb (lanes 6-8), marker in lane 1 (Red frame)

S2A4 (anti-TUT4) Nb (lanes 2-3), Gb (lanes 4-5), and DiGb (lanes 6-8), marker in lane 1 (Red frame)

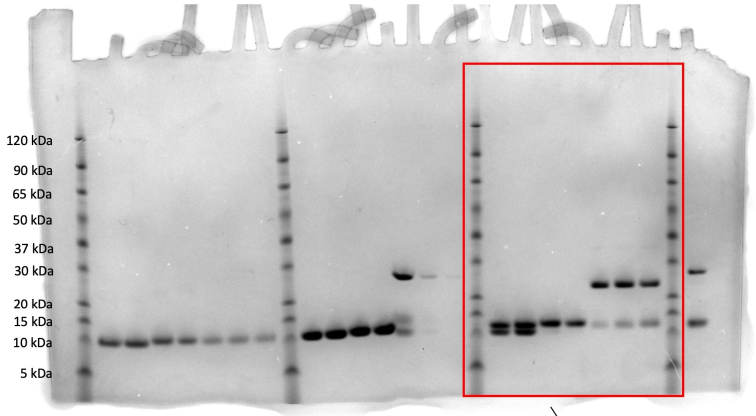

RBD6 (anti-RBD) Nb (lanes 2-3), Gb (lanes 4-5), and DiGb (lanes 6-8), marker in lane 1 (Red frame)

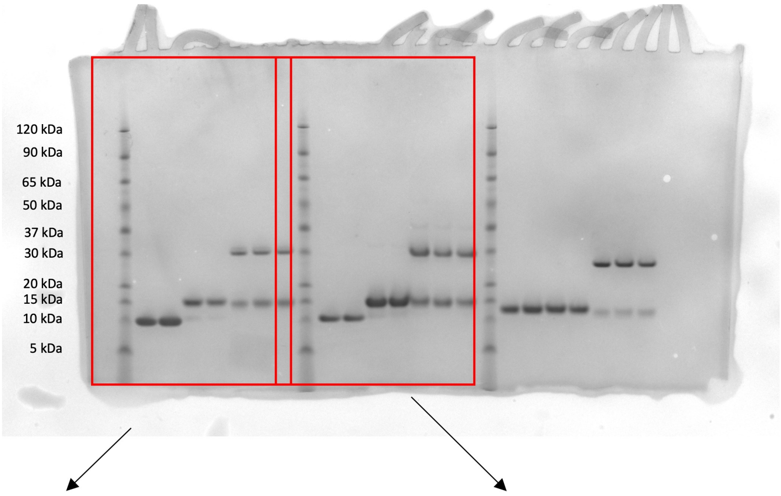

Anti-Lysozyme Nb (lanes 2-3), Gb (lanes 4-5), and DiGb (lanes 6-8), marker in lane 1 (Red frame)

Anti-HIV Nb (lanes 2-3), Gb (lanes 4-5), and DiGb (lanes 6-8), marker in lane 1 (Red frame)

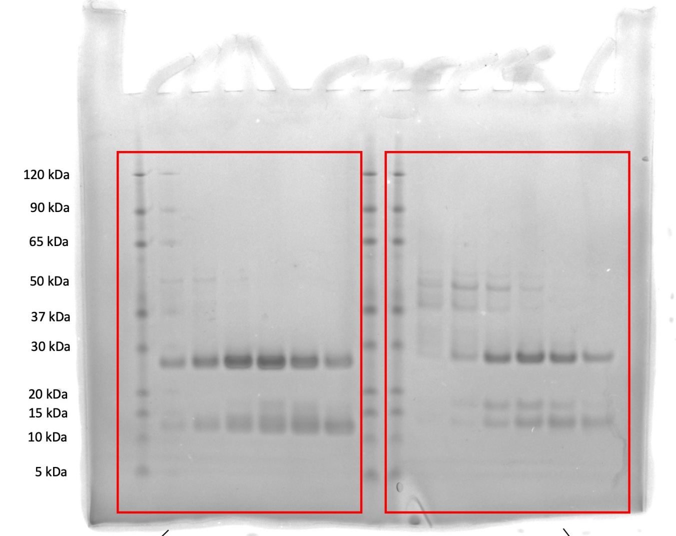

Hetero DiGb C4 (Anti-SPNS2):GFPenhancer, marker in lane 1 (Red frame)

Hetero DiGb 5006 (anti-RecQL5):GFPenhancer , marker in lane 1 (Red frame)

Supplementary Fig. 18 Source data of uncropped gels. Uncropped gels corresponding to a) Supplementary Figure 3, b) Supplementary Figure 4, c) Supplementary Figure 8 and d) Supplementary 15. Detailed annotations indicating the excised regions and individual lanes are provided at the bottom of the gels.

Supplementary Table 1

**Sample**	**RECQL5:homoDiGb**		**SPNS2:heteroDiGb:sfGFP**			**RECQL5:heteroDiGb:sfGFP**			**SPNS2:homoDiGb**	
Description	Dimer complex	RecQL5 (Symmetry Expansion)	Dimer complex	SPNS2 (Local Refine)	sfGFP (Local Refine)	Dimer complex	RecQL5 (Local Refine)	sfGFP (Local Refine)	Dimer complex	SPNS2 (Symmetry expansion)
**PDB ID**	8RL5	8RL6	8RLC	8RLD	8RLE	8RL9	8RLA	8RLB	8RL7	8RL8
**EMDB ID (EMD-)**	19331	19332	19338	19339	19340	19335	19336	19337	19333	19334
**Data collection**										
Microscope	OPIC Titan Krios							eBIC Titan Krios		
Detector	Falcon 4 with SelectrisX							Gatan K3 with GIF Quantum		
Cs (mm)	2.7	2.7	2.7	2.7	2.7	2.7	2.7	2.7	2.7	2.7
Magnification	165k	165k	130k	130k	130k	105k	105k	105k	105k	105k
Pixel size (Å)	0.73	0.73	0.93	0.93	0.93	0.93	0.93	0.93	0.83	0.83
Electron dose (e-/Å^{-2})	60	60	50	50	50	42	42	42	50	50
Defocus range (µm)	1.5-3	1.5-3	1.5-2.5	1.5-2.5	1.5-2.5	1.5-2.5	1.5-2.5	1.5-2.5	1.5-2.5	1.5-2.5
Micrograph Number	9,247	9,247	8,674	8,674	8,674	10,102	10,102	10,102	12,013	12,013
**Reconstruction**										
Particles refinement	146,540	194,417	321,178	321,178	321,178	359,811	359,811	359,811	440,552	570,074
Symmetry	C2	C1	C1	C1	C1	C1	C1	C1	C2	C1
Resolution (Å)	3.79	3.18	3.9	2.84	3.75	3.22	3.03	2.99	3.76	2.79
Gunier Map B-factor (Å^2)	182.6	157.2	131.4	84.6	154.9	128.5	111.1	103.6	148.8	100.8
**Model building and validation**										
**Model composition**										
**Number of atoms (Number of Hydrogen atoms)**	14338 (6678)	6493 (3217)	12945 (6364)	6448 (3241)	6618 (3190)	13188 (6427)	8306 (4093)	6896 (3339)	16330 (8110)	6503 (3286)
Protein residues	1112	436	866	422	450	898	560	463	1100	421
Ligand	ZN:2	ZN:1	DDM:1	DDM:1	-	ZN:1	ZN:1	-	DDM:1	DDM:1
**Map-to-Model resolution**										
FSC (Å)(0.5)	6.5	4.1	4.1	3.6	4.3	3.8	3.7	3.8	3.9	3.4
**Bonds RMSD**										
Bond Lengths (Å)	0.002	0.002	0.002	0.002	0.003	0.002	0.002	0.002	0.004	0.002
Bond Angles (°)	0.436	0.494	0.444	0.508	0.557	0.492	0.511	0.484	0.909	0.511
**Validation**										
MolProbity score	1.28	1.48	1.35	1.38	1.43	1.45	1.54	1.49	1.56	1.41
Clash score	5.29	9.09	6.32	6.81	7.85	8.40	10.36	9.27	7.65	7.53
Rotamer outliers	0.00	0.00	0.00	0.00	0.00	0.00	0.00	0.00	0.00	0.00
C-beta outliers	0.00	0.00	0.00	0.00	0.00	0.00	0.00	0.00	0.00	0.00
**Ramachandran plot**										
Favoured (%)	98.08	98.61	98.48	98.80	98.42	98.76	98.01	98.03	97.23	98.80
Allowed (%)	1.92	1.39	1.52	1.20	1.58	1.24	1.99	1.97	2.77	1.20
Outlier (%)	0.00	0.00	0.00	0.00	0.00	0.00	0.00	0.00	0.00	0.00

Sample	MBP:homoDiGb		Lysozyme:homoDiGb	
Description	Dimer complex	MBP (Symmetry Expansion)	Dimer complex	Lysozyme (Local Refine)
**PDB**	9FGV	9FKQ	9FGX	9FGY
**EMDB (EMD-)**	50430	50525	50432	50433
**Data collection**				
Microscope	Titan Krios			
Detector	Falcon 4 with SelectrisX			
Cs (mm)	2.7	2.7	2.7	2.7
Magnification	165k	165k	165k	165k
Pixel size (Å)	0.73	0.73	0.73	0.73
**Electron dose (e-/Å^2)**	50	50	50	50
Defocus range (µm)	1.2-2.6	1.2-2.6	1.6-2.6	1.6-2.6
**Micrograph Number**	14,291	14,291	10,063	10,063
**Reconstruction**				
software	cryoSPARC-4.0		cryoSPARC-4.0	
**Particles refinement**	1,536,548	1,536,548	1,266,854	1,266,854
symmetry	C2	C1	C1	C1
resolution (Å)	3.39	2.45	3.53	3.16
**Gunier Map B-factor (Å^2)**	119.5	74.7	143.3	109.1
**Model building and validation**				
**<u>Model composition</u>**				
**number of atoms (number of hydrogen atoms)**	7685 (0)	5602 (0)	7546 (3652)	3773 (1826)
protein residues	982	362	508	254
Ligand	-	-	GOL:2	GOL:1
**<u>Model resolution</u>**				
FSC (Å) (0.5)	3.7	3.2	7.2	3.9
**<u>Bonds RMSD</u>**				
Bonds lengths (Å)	0.002	0.002	0.002	0.001
Bonds angles (°)	0.453	0.486	0.405	0.398
**<u>Validation</u>**				
MolProbity score	1.33	1.11	1.40	1.46
Clash score	6.03	3.21	7.24	8.42
**Rotamer outliers (%)**	0.00	0.00	0.00	0.00
C-beta outliers (%)	0.00	0.00	0.00	0.00
**<u>Ramachandran plot</u>**				
Favoured (%)	98.46	99.44	98.20	98.80
Allowed (%)	1.54	0.56	1.80	1.20
Outlier (%)	0.00	0.00	0.00	0.00

Supplementary Table 1. Cryo-EM Data Collection, Processing, Refinement and Model Building and Validation

Supplementary Table 2

a

Structure	No. of interacting residues (<4Å) in the Gembody interface	No. of hydrogen bonds in the Gembody interface	Moving Gembody chain ID	Static Gembody chain ID	Intra-Glebody angle (°)
RECQL5:homoDiGb	28	5	L (Gb5-006)	K (Gb5-006)	71.32
SPNS2:homoDiGb	21	3	D (GbD12)	B (GbD12)	100.70
SPNS2:heteroDiGb:sfGFP	8	0	D (GbEnhancer)	C (GbC4)	96.34
RECQL5:heteroDiGb:sfGFP	26	4	D (GbEnhancer)	K (G5-006)	82.00

b

Structure	Max $\overrightarrow{D_a}$ wobble angle β (°)	75 quartile $\overrightarrow{D_a}$ wobble angle β (°)	Median $\overrightarrow{D_a}$ wobble angle β (°)
RECQL5:homoDiGb	4.04	1.22	0.78
SPNS2:homoDiGb	2.85	1.21	0.47
SPNS2:heteroDiGb:sfGFP	3.66	0.61	0.40
RECQL5:heteroDiGb:sfGFP	2.01	1.24	0.84

Supplementary Table 2. 3D Variability Analyses Summary

Supplementary Table 3

Model	Targets	Target Volume Proportion (%)	Complex Max Diameter (Å)	Target Max Diameter (Å)
**RECQL5:homoDiGb**	RECQL5	77.8	104.7	72.3
**MBP:homoDiGb**	MBP	75.4	108.3	64.5
**Lysozyme:homoDiGb**	Lysozyme	51.9	125.6	44.1
**SPNS2:homoDiGb**	SPNS2	80.8	178.4	65.8
**SPNS2:heteroDiGb:sfGFP**	SPNS2	52.6	116.5	65.8
	sfGFP	24		49.9
**RECQL5:heteroDiGb:sfGFP**	RECQL5	47.6	110.5	72.3
	sfGFP	26.2		49.9

Supplementary Table 3. DiGbs increase maximum particle diameters while maintaining the target volume proportions within the complex.

Supplementary Table 4 Sequences of Proteins

Wide type nanobody	Protein Sequences
**Nb5-006**	SMAQVQLVENSGGGLVQAGGS LRLSCAASGS IFSINRMTWY RQAPGKEREW VAAITSGGSTNYADSVKGRF TISRDNAENT VYLQMNSLKP EDTAVYYCEA YGTYTLAPTG EGEYDDYWGQGTQVTVS
**NbD12**	MAQVQLVESGGGLVQAGGSLRLSCAASGRLLSWYDMAWFRQAPGKEREFVAAVTSTGAGTHYVDSVKGRFTISRVNAKNTMYLQMNSLKPEDTAVYYCAAANTRLTALSLRTTTGSWAYWGKGTPVTVS
**NbS2A4**	MAQVQLQESGGGLVQAGGSLRLSCAASGTIFTYFVMGWYRQAPGKERELVAGITLGGTTYYADSVKGRFTISRDNAKNTVYLQMNSLKPEDTAVYYCAAWVEYPRRYVYWGQGTQVTVS
**NbEnhancer**	QVQLVESGGALVQPGGSLRLSCAASGFPVNRYSMRWYRQAPGKEREWVAGMSSAGDRSSYEDSVKGRFTISRDDARNTVYLQMNSLKPEDTAVYYCNVNVGFEYWGQGTQVTV
**NbH12**	MAQLQLVESGGGLVQPGGSLRLSCAASGSRFSDNTMAWYRQAPRKQRELVARIPMGGRPMYADSVKGRFTISRDNAKNTVYLQMNSLKPDDTAVYYCNAVTYGLESYWGKGTPVTVS
**NbLysozyme**	DVQLVESGGGLVQAGGSLRLSCAASGSTDSIEYMTWFRQAPGKAREGVAALYTHTGNTYYTDSVKGRFTISQDKAKNMAYLRMDSVKSEDTAIYTCGATRKYVPVR-FALDQSSYDYWGQGTQVTV
**NbHIV**	DVQLQESGGGLVQAGGSLRLSCAASGSISRFNAMGWWRQAPGKEREFVARIVKGFDPVLADSVKGRFTISIDSAENTLALQMNRLKPEDTAVYYCFAALDTAYWGQGTQVTV
**NbRBD2**	EVQLVESGGGLVQTGGSLRLSCALSGYTFSIFPTAWFRQAPGKEREFVAGIRWNGSTRDYTEYADFVKGRFTISRDNAENMVYLQMISLKPEDTALYYCAASDGVIDGTNANAYRYWGQGTQVTVSS
**NbRBD3**	HVQLVESGGGLVQAGGSLRLSCATSGRTFSTYRMSWFRQAPGKEREFVATIIWSVGSTHYADSVKGRFTISRDNAKNMVYLQMNSLKPEDTAVYYCAAQRSDSSSWGYEDDYDYWGQGTQVTVSS

**NbMBP**	QVQLVESGGGLVQAGGSLRLSCVASGDIKYISYLGWFRQAPGKEREGVAALYT<br>STGRTYYADSVKGRFTVSLDNAKNTVYLQMNSLKPEDTALYYCAAAEWGSQS<br>PLTQWFYRYWGQGTQVTV
**NbC4**	QLQLVESGGGLVQAGGSLRLSCAASQGTLSNLVTGWFRRAPGKEREFVANIG<br>RDGLTVYSNSVKGRFTISRDRAKNTVYLQMDSLKPEDTAVYYCAGRLSRFPGEY<br>DYWSKGTPVTVSSSQ
**NbRBD1**	QVQLVESGGGLMQAGGSLRLSCAVSGRTFSTAAMGWFRQAPGKEREFVAAI<br>RWSGGSAYYADSVKGRFTISRDKAKNTVYLQMNSLKYEDTAVYYCAQTRVTRS<br>LLSDYATWPYDYWGQGTQVTVS
**NbRBD6**	QVQLVESGGGLVQAGGSLRLACIASGRTFHSYVMAWFRQAPGKEREFVAAIS<br>WSSTPTYYGESVKGRFTISRDNAENTVYLQMNRLKPEDTAVYFCAADRGESYY<br>YTRPTEYEFWGQGTQVTVS

**Mutant Gembody**	**Protein Sequences**
**Gb5-006**	SMAQVQLVEN GGGCVKAGGS LRLSCAASGS IFSINRMTWY<br>RQAPGKEREW VAAITSGGSTNYADSVKGRF TISRDNAENT VYLQMNSLKP<br>EDTAVYYCEA YGTYTLAPTG EGEYDDYWGQGTQVMVS
**GbD12**	MAQVQLVENGGGCVKAGGSLRLSCAASGRLLSWYDMAWFRQAPGKEREFV<br>AAVTSTGAGTHYVDSVKGRFTISRVNAKNTMYLQMNSLKPEDTAVYYCAAAN<br>TRLTALSLRTTTGSWAYWGKGTPVMVS
**GbS2A4**	MAQVQLQENGGGCVKAGGSLRLSCAASGTIFTYFVMGWYRQAPGKERELVA<br>GITLGGTTYYADSVKGRFTISRDNAKNTVYLQMNSLKPEDTAVYYCAAWVEYP<br>RRYVYWGQGTQVMVS
**GbEnhancer**	QVQLVENGGGCVKPGGSLRLSCAASGFPVNRYSMRWYRQAPGKEREWVAG<br>MSSAGDRSSYEDSVKGRFTISRDDARNTVYLQMNSLKPEDTAVYYCNVNVGF<br>EYWGQGTQVMV
**GbH12**	MAQLQLVENGGGCVKPGGSLRLSCAASGSRFSDNTMAWYRQAPRKQRELV<br>ARIPMGGRPMYADSVKGRFTISRDNAKNTVYLQMNSLKPDDTAVYYCNAVTY<br>GLESYWGKGTPVMVS

**GbLysozyme**	MADVQLVENGGGCVKAGGSLRLSCAASGSTDSIEYMTWFRQAPGKAREGVA ALYTHTGNTYYTDSVKGRFTISQDKAKNMAYLRMDSVKSEDTAIYTCGATRKA VPVRFALDQSSYDYWGQGTQVMVSSSAG
**GbHIV**	MADVQLQENGGGCVKAGGSLRLSCAASGSISRFNAMGWWRQAPGKEREFV ARIVKGFDPVLADSVKGRFTISIDSAENTLALQMNRLKPEDTAVYYCFAALDTXX AYWGQGTQVMVSSAAADYKPGGGKPGGEPEA
**GbRBD2**	EVQLVENGGGCVKTGGSLRLSCALSGYTFSIFPTAWFRQAPGKEREFVAGIRW NGSTRDYTEYADFVKGRFTISRDNAENMVYLQMISLKPEDTALYYCAASDGVI DGTNANAYRYWGQGTQVMVSS
**GbRBD3**	HVQLVENGGGCVKAGGSLRLSCATSGRTFSTYRMSWFRQAPGKEREFVATII WSVGSTHYADSVKGRFTISRDNAKNMVYLQMNSLKPEDTAVYYCAAQRSDSS SWGYEDDYDYWGQGTQVMVSS
**GbMBP**	QVQLVENGGGCVKAGGSLRLSCVASGDIKYISYLGWFRQAPGKEREGVAALYT STGRTYYADSVKGRFTVSLDNAKNTVYLQMNSLKPEDTALYYCAAAEWGSQS PLTQWFYRYWGQGTQVMV
**GbC4**	QLQLVENGGGCVKAGGSLRLSCAASQGTLSNLVTGWFRRAPGKEREFVANIG RDGLTVYSNSVKGRFTISRDRAKNTVYLQMDSLKPEDTAVYYCAGRLSRFPGEY DYWSKGTPVMVSSSQ
**GbRBD1**	QVQLVENGGGCVKAGGSLRLSCAVSGRTFSTAAMGWFRQAPGKEREFVAAI RWSGGSAYYADSVKGRFTISRDKAKNTVYLQMNSLKYEDTAVYYCAQTRVTRS LLSDYATWPYDYWGQGTQVMVS
**GbRBD6**	QVQLVENGGGCVKAGGSLRLACIASGRTFHSYVMAWFRQAPGKEREFVAAIS WSSTPTYYGESVKGRFTISRDNAENTVYLQMNRLKPEDTAVYFCAADRGESYY YTRPTEYEFWGQGTQVMVS
**Gb_anti Stx2aB**	QVQLQENGGGCVKPGGSLRLSCAASGFTFSSYYMSWVRQAPGKGPEWVSGI NTGGVGTRYADSVKGRFTISRDNAKNTLYLQMNSLKPEDTALYYCAIGEGGNR NYWGQGTQVMVSS

**Target protein**	**Protein Sequences**
**RecQL5A**	SMDPERRVRSTLKKVFGFDSFKTPLQESATMAVVKGNKDVFVCMPTGAGKSL<br>CYQLPALLAKGITIVVSPLIALIQDQVDHLLTLKVRVSSLNSKLSAQERKELLADLE<br>REKPQTKILYITPEMAASSSFQPTLNSLVSRHLLSYLVVDEAHCVSQWGHDFRP<br>DYLRLGALRSRLGHAPCVALTATATPQVQEDVFAALHLKKPVAIFKTPCFRANL<br>FYDVQFKELISDPYGNLKDFCLKALGQEADKGLSGCGIVYCRTREACEQLAIELS<br>CRGVNAKAYHAGLKASERTLVQNDWMEEKVPVIVATISFGMGVDKANVRFV<br>AHWNIAKSMAGYYQESGRAGRDGKPSWCRLYYSRNDRDQVSFLIRKEVAKLQ<br>EKRGNKASDKATIMAFDALVTFCEELGCRHAAIAKYFGDALPACAKGCDHCQN<br>PTAVRRRLEALERSSSW
**SPNS2**	MMCLECASAAAGGAEEEEADAERRRRRRGAQRGAGGSGCCGARGAGGAG<br>VSAAGDEVQTLSGSVRRAPTGPPGTPGTPGCAATAKGPGAQQPKPASLGRGR<br>GAAAAILSLGNVLNYLDRYTVAGVLLDIQQHFGVKDRGAGLLQSVFICSFMVA<br>APIFGYLGDRFNRKVILSCGIFFWSAVTFSSSFIPQQYFWLLVLSRGLVGIGEASY<br>STIAPTIIGDLFTKNTRTLMLSVFYFAIPLGSGLGYITGSSVKQAAGDWHWALRV<br>SPVLGMITGTLILILVPATKRGHADQLGDQLKARTSWLRDMKALIRNRSYVFSS<br>LATSAVSFATGALGMWIPLYLHRAQVVQKTAETCNSPPCGAKDSLIFGAITCFT<br>GFLGVVTGAGATRWCRLKTQRADPLVCAVGMLGSAIFICLIFVAAKSSIVGAYI<br>CIFVGETLLFSNWAITADILMYVVIPTRRATAVALQSFTSHLLGDAGSPYLIGFIS<br>DLIRQSTKDSPLWEFLSLGYALMLCPFVVVLGGMFFLATALFFVSDRARAEQQ<br>VNQLAMPPASVKVAENLYFQ
**MBP**	MKIEEGKLVIWINGDKGYNGLAEVGKKFEKDTGIKVTVEHPDKLEEKFPQVAA<br>TGDGPDIIFWAHDRFGGYAQSGLLAEITPDKAFQDKLYPFTWDAVRYNGKLIA<br>YPIAVEALSLIYNKDLLPNPPKTWEEIPALDKELKAKGKSALMFNLQEPYFTWPLI<br>AADGGYAFKYENGKYDIKDVGVDNAGAKAGLTFLVDLIKNKHMNADTDYSIA<br>EAAFNKGETAMTINGPWAWSNIDTSKVNYGVTVLPTFKGQPSKPFVGVLSAG<br>INAASPNKELAKEFLENYLLTDEGLEAVNKDKPLGAVALKSYEEELAKDPRIAAT<br>MENAQKGEIMPNIPQMSAFWYAVRTAVINAASGRQTVDEALKDAQTNSGG<br>SHHHHHHSSGVDLGTENLYFQ

**sfGFP**	MSKGEELFTGVVPILVELDGDVNGHKFSVRGEGEGDATNGKLTLKFICTTGKLP VPWPTLVTTLTYGVQCFSRYPDHMKRHDFFKSAMPEGYVQERTISFKDDGTY KTRAEVKFEGDTLVNRIELKGIDFKEDGNILGHKLEYNFNSHNVYITADKQKNGI KANFKIRHNVEDGSVQLADHYQQNTPIGDGPVLLPDNHYLSTQSVLSKDPNEK RDHMVLLEFVTAAGITHGMDELYK
**Lysozyme**	KVFGRCELAAAMKRHGLDNYRGYSLGNWVCAAKFESNFNTQATNRNTDGST DYGILQINSRWWCNDGRTPGSRNLCNIPCSALLSSDITASVNCAKKIVSDGNG MNAWVAWRNRCKGTDVQAWIRGCRL
**Stx2aB**	ADCAKGKIEFSKYNEDDTFTVKVDGKEYWTSRWNLQPLLQSAQLTGMTVTIKS STCESGSGFAEVQFNNDLE

Supplementary Table 5 Sequences of Primers

Forward Primer names	Forward primers
**Nb5-006-F**	TACTTCCAATCCATGGCTCAAGTACAGCTAGTTGA
**NbD12-F**	TACTTCCAATCCATGGCTCAAGTTCAGCTAGTA
**NbS2A4-F**	TACTTCCAATCCATGGCTCAAGTACAGCTACAAGAA
**NbEnhancer-F**	TACTTCCAATCCCAAGTACAGCTAGTTGAATCAGGG
**NbH12-F**	TACTTCCAATCCATGGCTCAATTACAGCTAGTAGAAT
**NbLysozyme-F**	TACTTCCAATCCATGGCTCAATTACAGCTAGTAGAATCA
**NbHIV-F**	TACTTCCAATCCGATGTACAGCTACAAGAATCAGGTG
**NbRBD2-F**	TACTTCCAATCCGAGGTTCAACTAGTAGAATCAGGTGG
**NbRBD3-F**	TACTTCCAATCCCACGTTCAACTAGTAGAATCAGGTGG
**NbC4-F**	TACTTCCAATCCCAATTACAGCTAGTAGAATCAGGTGGA
**NbRBD1-F**	TACTTCCAATCCCAAGTACAGCTAGTTGAATCAGGTGG
**NbRBD6-F**	TACTTCCAATCCCAAGTTCAGCTAGTAGAATCAGGTGG
**Nb_MBP**	TACTTCCAATCC CAAGTTCAGCTAGTAGA
**Gb5-006-F**	TACTTCCAATCCATGGCGCAAGTGCAACTGGTT
**GbD12-F**	TACTTCCAATCCATGGCGCAAGTGCAACTGGTT
**GbS2A4-F**	TACTTCCAATCCATGCAAGTGCAACTGGTTGAGAA
**GbEnhancer-F**	TACTTCCAATCCCAGGTTCAGCTGGTTGAAaacGGTGGTGCAtgcGTTaaaCCT GGTGGTAG
**GbH12-F**	TACTTCCAATCCATGGCGCAAGTGCAACTGGTT

**GbLysozyme-F**	TACTTCCAATCC GATGTTCAACTAGTAGAA
**GbHIV-F**	TACTTCCAATCC GATGTACAGCTACAAGAA
**GbRBD2-F**	TACTTCCAATCC GAGGTTCAACTAGTAGAA
**GbRBD3-F**	TACTTCCAATCC CACGTTCAACTAGTAGAA
**GbC4-F**	TACTTCCAATCC TCCCAGGGGCAGCTCGTGGAGAATGGGGGAGGCTGTGTGAAGGCTGGGGG CTCTCTGAGA
**GbRBD1-F**	TACTTCCAATCC CAAGTTCAGCTAGTAGAA
**GbRBD6-F**	TACTTCCAATCCCAAGTTCAGCTAGTAGAA
**Gb_MBP-F**	TACTTCCAATCCCAGGTTCAGCTGGTTGAAaacGGTGGTGGTtgcGTTaaaGCA GGCGGTAG
**Stx2aB_Gb-F**	TACTTCCAATCC CAAGTACAGCTACAAGA
**Stx2Ab-F**	TATCCACCTTTACTG TTA GCTGATTGCGCCAAAGG
**Reverse Primer names**	Reverse Primers
**Nb5-006-R**	TATCCACCTTTACTGTTAGCTCACCGTAACCTGTGTAC
**NbD12-R**	TATCCACCTTTACTGTTAGCTCACGGTAACCGGGGT
**NbS2A4-R**	TATCCACCTTTACTGTTACGTACCCTGGCCCCAATACA
**NbEnhancer-R**	TATCCACCTTTACTGTTACACGGTAACTTGCGTACCC
**NbH12-R**	TATCCACCTTTACTGTTAGCTCACGGTAACCGGAGTA
**NbLysozyme-R**	TATCCACCTTTACTGTTAGGACACGGTAACCGGAGT
**NbHIV-R**	TATCCACCTTTACTGTTATGCCTCCGGTTCGCCGCC
**NbRBD2-R**	TATCCACCTTTACTGTTAGCTGCTGACTGTCACTTGCG
**NbRBD3-R**	TATCCACCTTTACTGTTAGGAGCTAACCGTCACTTGCG

**NbC4-R**	TATCCACCTTTACTGTTATTGCGAGGAGCTCACCGT
**NbRBD1-R**	TATCCACCTTTACTGTTAGCTTACCGTAACCTGTGTACCTT
**NbRBD6-R**	TATCCACCTTTACTG TTA GCTGACGGTAACCTGGGT
**Nb_MBP-R**	TATCCACCTTTACTG TTA CACAGTAACCTGGGTGCC
**Gb5-006-R**	TATCCACCTTTACTGTTAGCTCACGGTCACCTGCGTACCCTGG
**GbD12-R**	TATCCACCTTTACTGTCAGCTCACCATCACCTGCGTACC
**GbS2A4-R**	TATCCACCTTTACTGTCAGCTCACCATCACCTGCGTACC
**GbEnhancer-R**	TATCCACCTTTACTGTCAgctAACcatAACCTGGGTGCCCTGACCCCAA
**GbH12-R**	TATCCACCTTTACTGTCAGCTCACCATCACCTGCGTACC
**GbLysozyme-R**	TATCCACCTTTACTG TTA GCCTGCGCTGGAGCTCAC
**GbHIV-F**	TATCCACCTTTACTG TTA AGCCTCCGGTTCACCGCC
**GbRBD2-R**	TATCCACCTTTACTG TTA GCTGCTGACCATAACCTG
**GbRBD3-R**	TATCCACCTTTACTG TTA CGAGCTGACCATAACCTG
**GbC4-R**	TATCCACCTTTACTG TTA CTGTGAACTGGAGACCATGACCGGGGTCCC
**GbRBD1-R**	TATCCACCTTTACTG TTA GCTGGAAACCATAACCTG
**GbRBD6-R**	TATCCACCTTTACTG TTA CGAGCTCACCATAACCTG
**Gb_MBP-R**	TATCCACCTTTACTGTCAgctAACcatAACCTGGGTGCCCTGACCCCA
**Stx2aB_Gb-R**	TACTTCCAATCC ACTGCTGACCATAACCT
**Stx2aB -R**	TATCCACCTTTACTG TTA TTCCAGATCGTTGTTAAA

Source data of Mass Spectrometry

Anti-SPNS2 Gembody GbH12

```
        10         20         30         40         50         60
SMAQVQLVEN GGGCVKPGGS LRLSCAASGS RFSDNTMAWY RQAPRKQREL VARIPMGGRP

        70         80         90        100        110
MYADSVKGRF TISRDNAENT VYLQMNSLKP DDTAVYYCNA VTYGLESYWG KGTQVMVS
```

Molecular weight: 12970.71 g/mol

Ext. coefficient: 21430 M^{-1} cm^{-1}

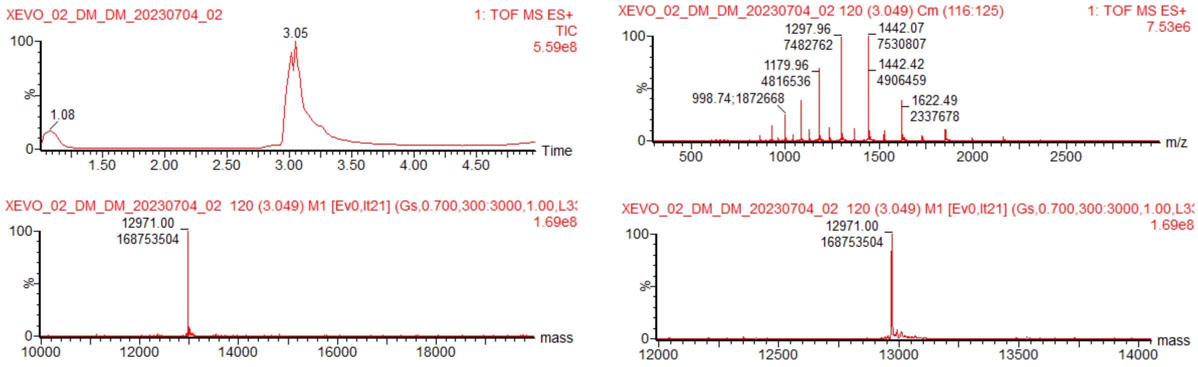

Calculated mass of Gembody GbH12: 12969, Observed mass: 12971

Anti-GFP Gembody

```
         10         20         30         40         50         60
SQVQLVENGG ACVKPGGSLR LSCAASGFPV NRYSMRWYRQ APGKEREWVA GMSSAGDRSS

         70         80         90        100        110
YEDSVKGRFT ISRDDARNTV YLQMNSLKPE DTAVYYCNVN VGFEYWGQGT QVMVS
```

Molecular weight: 12763.23 g/mol

Ext. coefficient: 26930 M^{-1} cm^{-1}

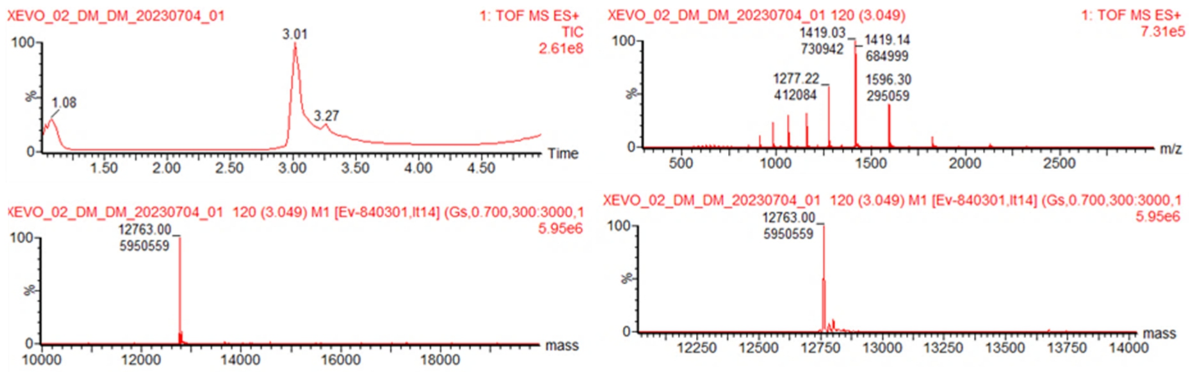

Calculated mass of Gembody GbEnhancer: 12761, Observed mass: 12763

Anti-RECQL5 Gembody Gb5-006

```
         10         20         30         40         50         60
SMAQVQLVEN GGGCVKAGGS LRLSCAASGS IFSINRMTWY RQAPGKEREW VAAITSGGST

         70         80         90        100        110        120
NYADSVKGRF TISRDNAENT VYLQMNSLKP EDTAVYYCEA YGTYTLAPTG EGEYDDYWGQ

GTQVMVS
```

Molecular weight: 13765.23 g/mol

Ext. coefficient: 29910 M^{-1} cm^{-1}

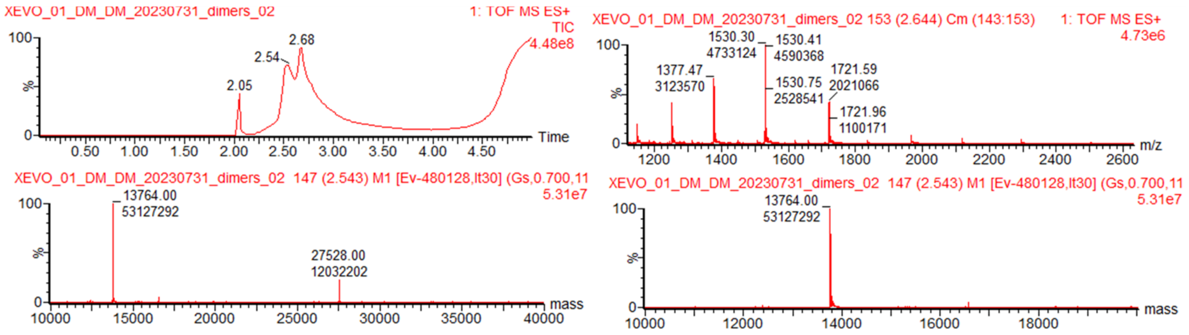

Calculated mass of Gembody Gb5-006: 13763, Observed mass: 13764

Anti-TUT4 Gembody GbS2A4

```
        10         20         30         40         50         60
SMAQVQLVEN GGGCVKAGGS LRLSCAASGT IFTYFVMGWY RRAPGKEREL VAGITLGGTT

        70         80         90        100        110        120
YYADSVKGRF TISRDNAKNT VYLQMNSLKP EDTAVYYCAA WVEYPRRYVY WGQGTQVMVS
```

Molecular weight: 13262.11 g/mol

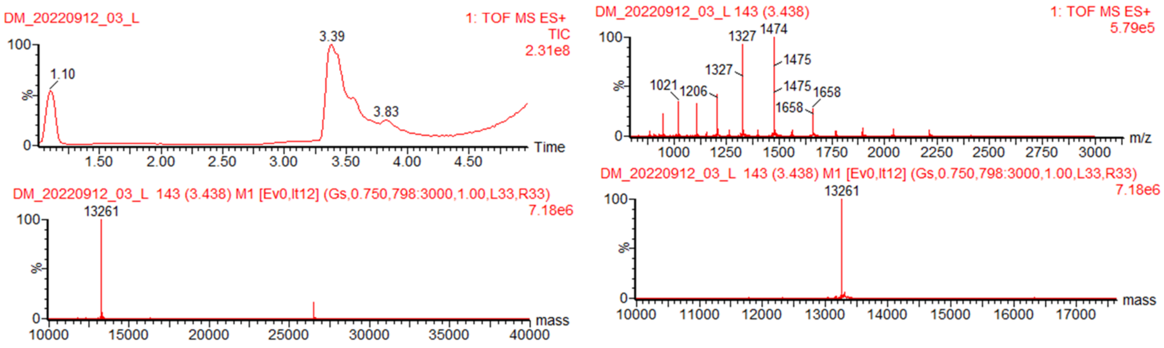

Ext. coefficient: 31400 M^{-1} cm^{-1}

Calculated mass of Gembody GbS2A4: 13260, Observed mass: 13261

Anti-MBP Gembody GbMBP

```
         10         20         30         40         50         60
SQVQLVENGG GCVKAGGSLR LSCVASGDIK YISYLGWFRQ APGKEREGVA ALYTSTGRTY

         70         80         90        100        110        120
YADSVKGRFT VSLDNAKNTV YLQMNSLKPE DTALYYCAAA EWGSQSPLTQ WFYRYWGQGT


QVMVS
```

Molecular weight: 13776.48 g/mol

Ext. coefficient: 36900 M^{-1} cm^{-1}

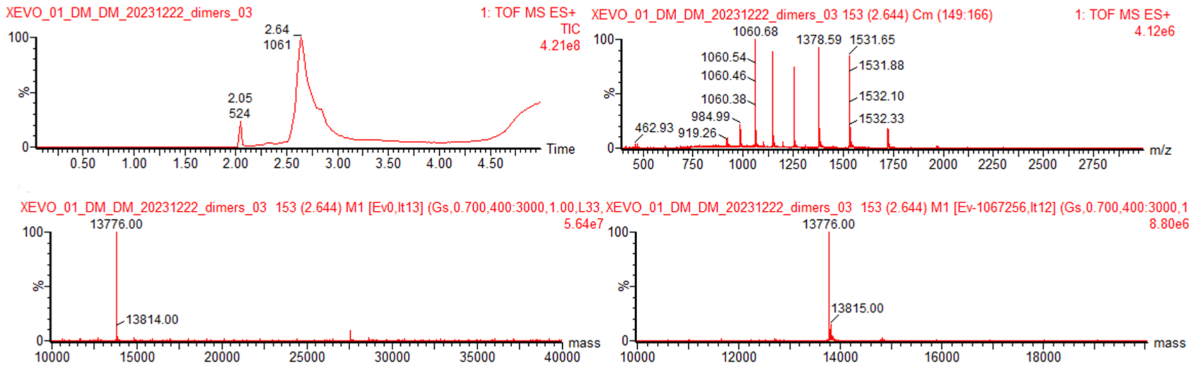

Calculated mass of Gembody GbMBP: 13774, Observed mass: 13776

Anti-SPNS2 Gembody GbD12

```
10         20         30         40         50         60
SMAQVQLVEN GGGCVKAGGS LRLSCAASGR LLSWYDMAWF RQAPGKEREF VAAVTSTGAG

        70         80         90        100        110        120
THYVDSVKGR FTISRVNAEN TMYLQMNSLK PEDTAVYYCA AANTRLTALS LRTTTGSWAY

       130
WGKGTQVMVS
```

Molecular weight: 14044.94 g/mol

Ext. coefficient: 30940 M^{-1} cm^{-1}

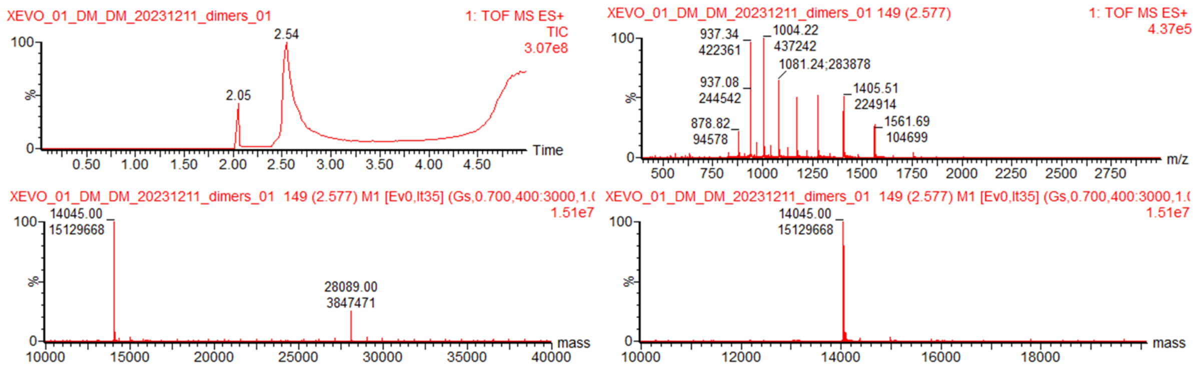

Calculated mass of Gembody GbD12: 14043, Observed mass: 14045

Anti-SPNS2 Gembody GbC4

```
         10         20         30         40         50         60
SQGQLVENGG GCVKAGGSLR LSCAASQGTL SNLVTGWFRR APGKEREFVA NIGRDGLTVY

         70         80         90        100        110        120
SNSVKGRFTI SRDRAKNTVY LQMDSLKPED TAVYYCAGRL SRFPGEYDYW SKGTPVMVSS

SQ
```

Molecular weight: 13268.90 g/mol

Ext. coefficient: 19940 M^{-1} cm^{-1}

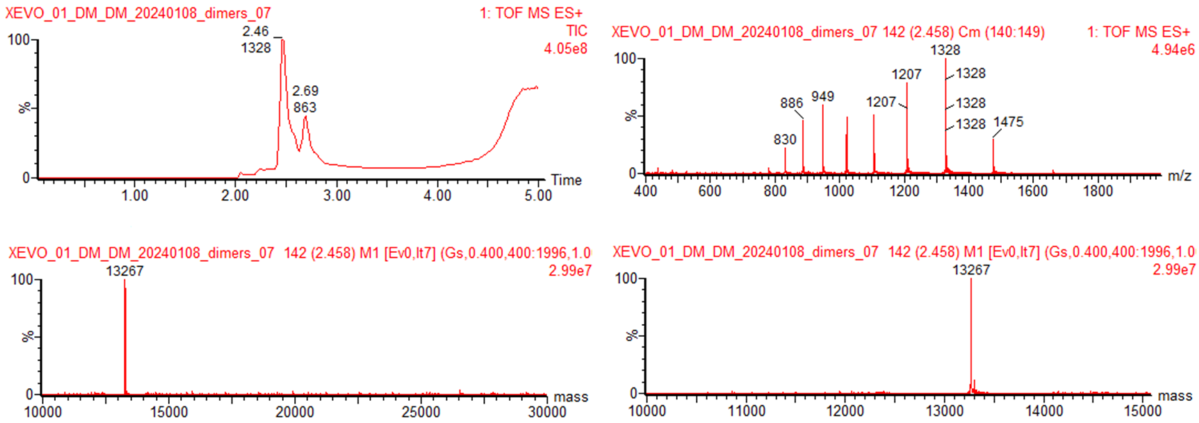

Calculated mass of Gembody F9 12Cys: 13267, Observed mass: 13267

Anti-SARS-COV2-SpikeRBD Gembody GbRBD1

```
        10         20         30         40         50         60
SQVQLVENGG GCMKAGGSLR LSCAVSGRTF STAAMGWFRQ APGKEREFVA AIRWSGGSAY

        70         80         90        100        110        120
YADSVKGRFT ISRDKAENTV YLQMNSLKYE DTAVYYCAQT RVTRSLLSDY ATWPYDYWGQ

GTQVMVS
```

Molecular weight: 14072.81 g/mol

Ext. coefficient: 35410 M^{-1} cm^{-1}

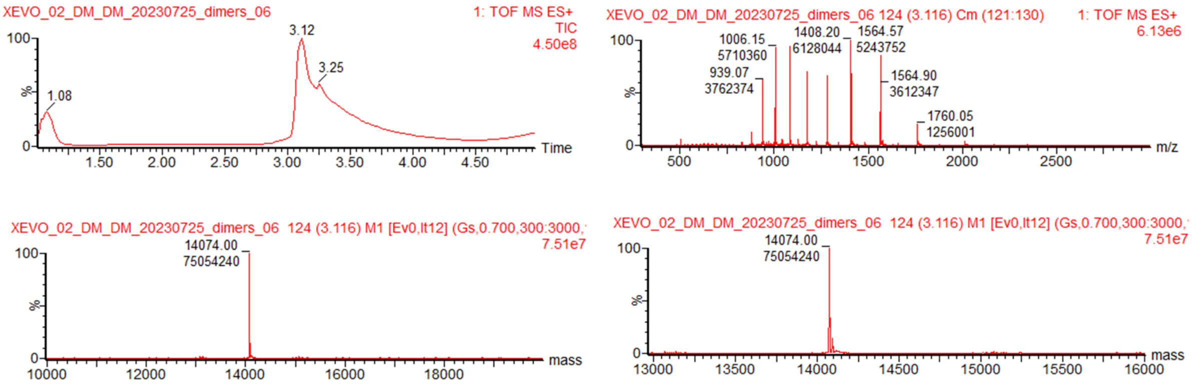

Calculated mass of Gembody GbRBD1: 14071, Observed mass: 14074

Anti-SARS-COV2-SpikeRBD Gembody GbRBD3

```
        10         20         30         40         50         60
HVQLVENGGG CVKAGGSLRL SCATSGRTFS TYRMSWFRQA PGKEREFVAT IIWSVGSTHY

        70         80         90        100        110        120
ADSVKGRFTI SRDNAENMVY LQMNSLKPED TAVYYCAAQR SDSSSWGYED DYDYWGQGTQ
```

VMVSS

Molecular weight: 13950.42

Ext. coefficient: 33920 M^{-1} cm^{-1}

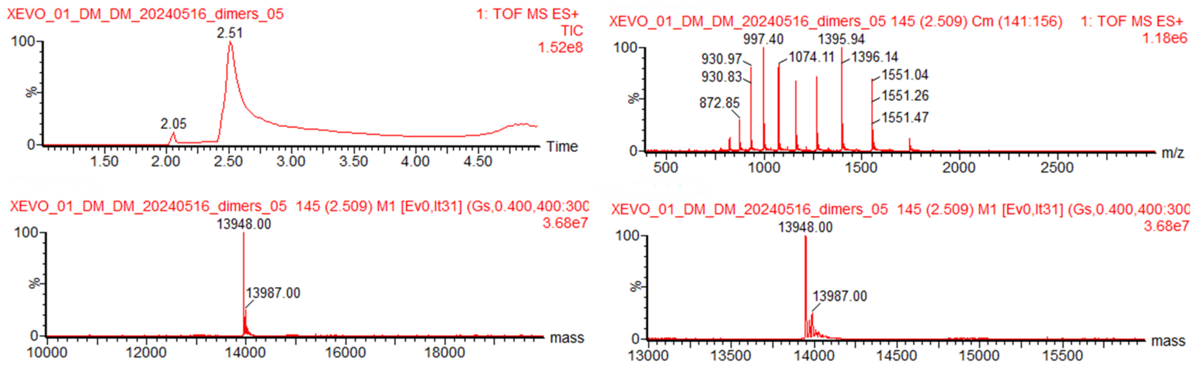

Calculated mass of Gembody GbRBD3: 13948, Observed mass: 13948

Anti-SARS-COV2-SpikeRBD Gembody GbRBD6

```
         10         20         30         40         50         60
SQVQLVENGG GCVKAGGSLR LACIASGRTF HSYVMAWFRQ APGKEREFVA AISWSSTPTY

         70         80         90        100        110        120
YGESVKGRFT ISRDNAENTV YLQMNRLKPE DTAVYFCAAD RGESYYYTRP TEYEFWGQGT

QVMVS
```

Molecular weight: 14038.69 g/mol

Ext. coefficient: 29910 M^{-1} cm^{-1}

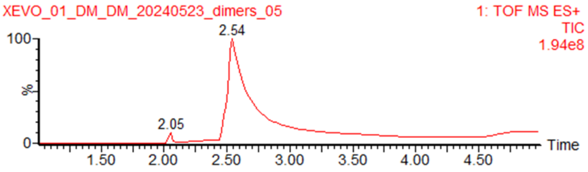

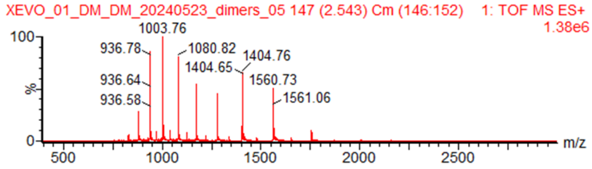

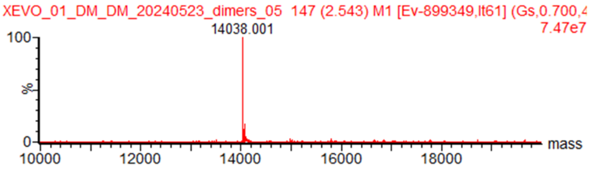

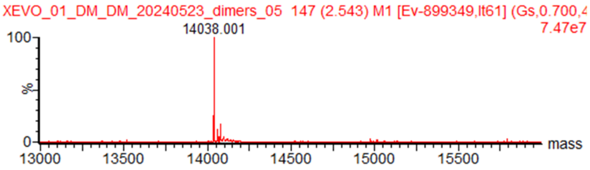

Calculated mass of Gembody GbRBD6: 14037, Observed mass: 14038

Anti-Lysozyme Gembody GbLys

```
        10         20         30         40         50         60
SDVQLVENGG GCVKAGGSLR LSCAASGSTD SIEYMTWFRQ APGKAREGVA ALYTHTGNTY

        70         80         90        100        110        120
YTDSVKGRFT ISQDKAKNMA YLRMDSVKSE DTAIYTCGAT RKYVPVRFAL DQSSYDYWGQ

       130
GTQVMVSSSA G
```

Molecular weight: 14155.76 g/mol

Ext. coefficient: 24410 M^{-1} cm^{-1}

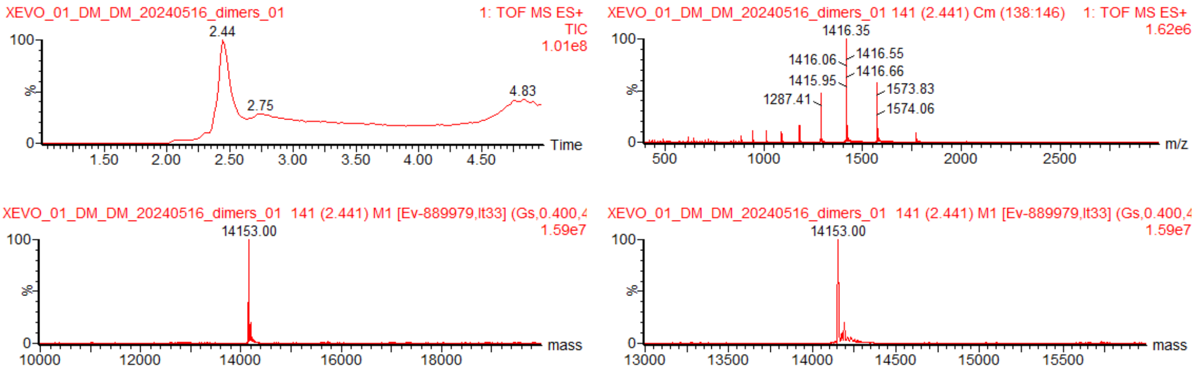

Calculated mass of Gembody GbLys: 14154, Observed mass: 1415

Anti-HIV Gembody GbHIV

```
        10         20         30         40         50         60
SDVQLQENGG GCVKAGGSLR LSCAASGSIS RFNAMGWWRQ APGKEREFVA RIVKGFDPVL

        70         80         90        100        110        120
ADSVKGRFTI SIDSAENTLA LQMNRLKPED TAVYYCFAAL DTAYWGQGTQ VMVSSAAADY

       130
KPGGGKPGGE PEA
```

Molecular weight: 14067.81 g/mol

Ext. coefficient: 22460 M^{-1} cm^{-1}

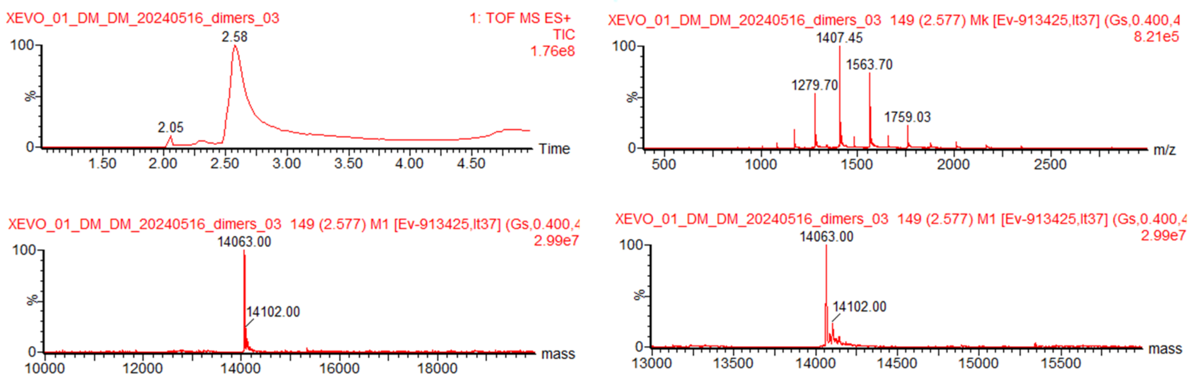

Calculated mass of Gembody GbHIV: 14066, Observed mass: 14063

General Protocol for the Chemical Dimerization of homo Di-Gembody

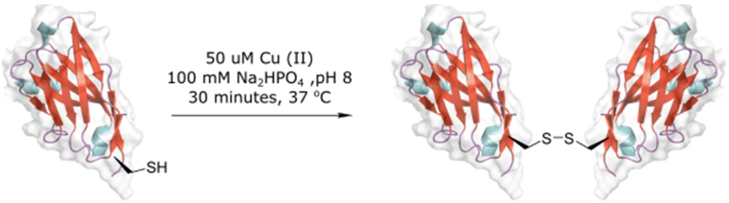

GbS2A4 homoDiGb

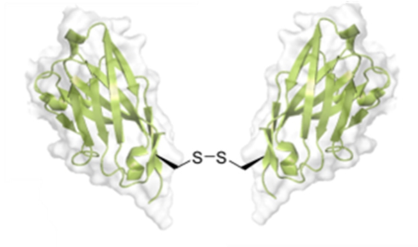

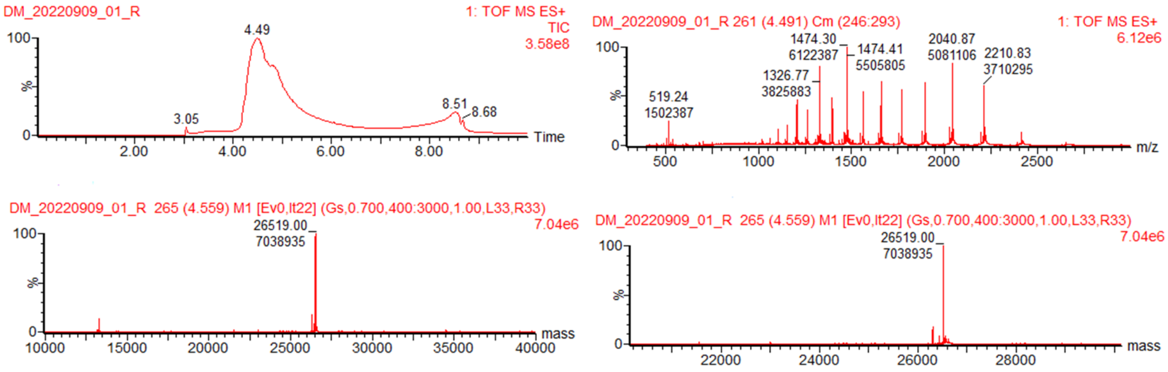

Calculated mass of GbS2A4 homoDiGb: 26518, Observed mass: 26519

Gb5-006 homoDiGb

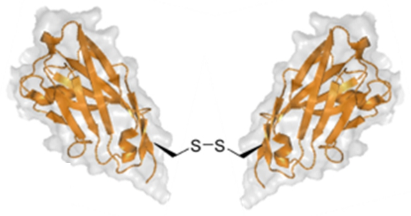

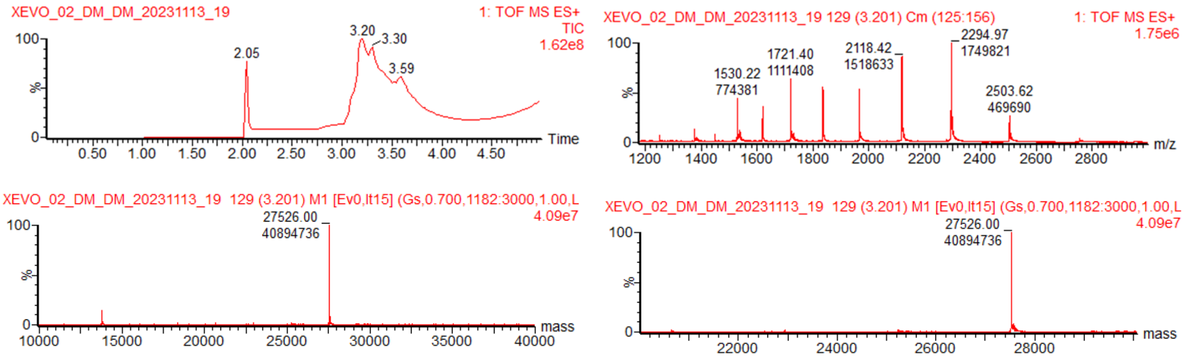

Calculated mass of Gb5-006 homoDiGb: 27524, Observed mass: 27526

GbH12 homoDiGb

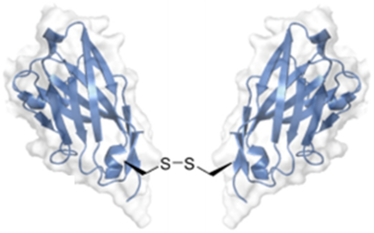

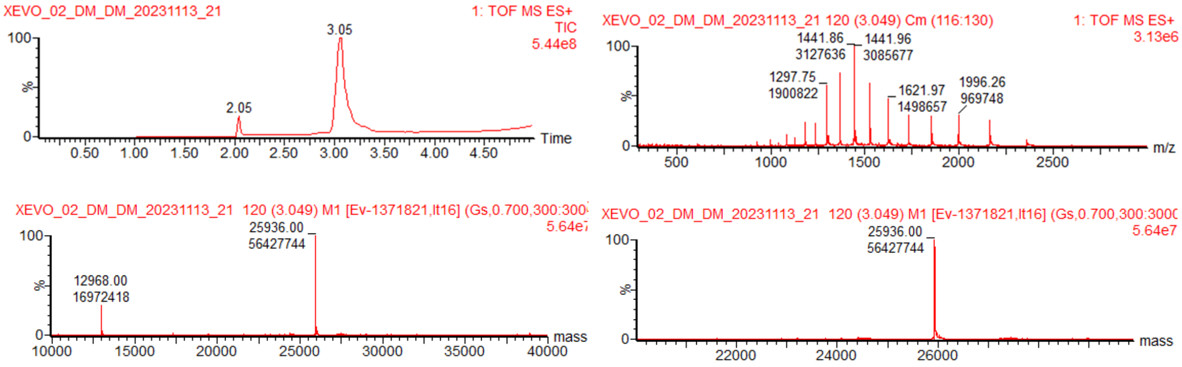

Calculated mass of GbH12 homoDiGb: 25936, Observed mass: 25936

GbEnhancer homoDiGb

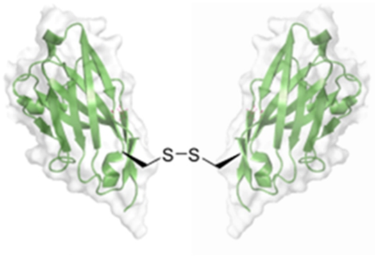

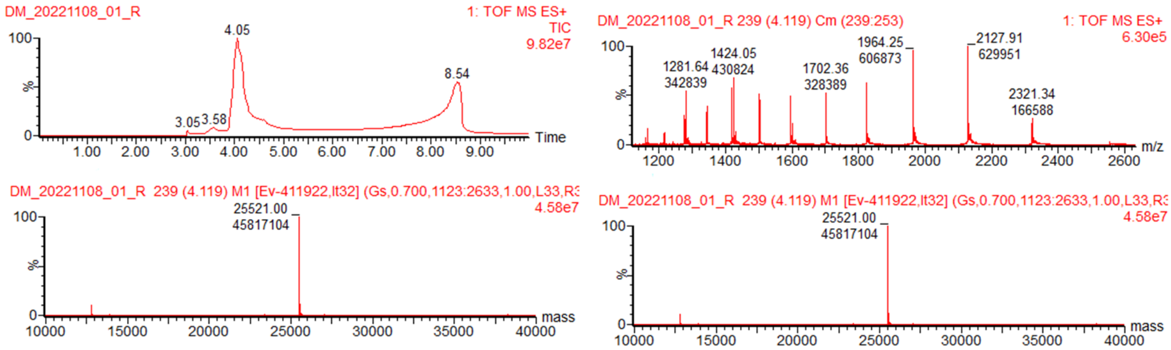

Calculated mass of GbEnhancer homoDiGb: 25520, Observed mass: 25521

GbD12 homoDiGb

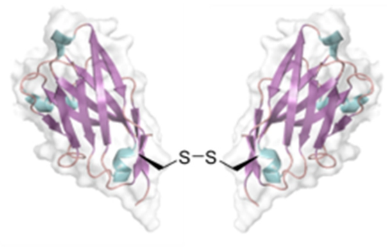

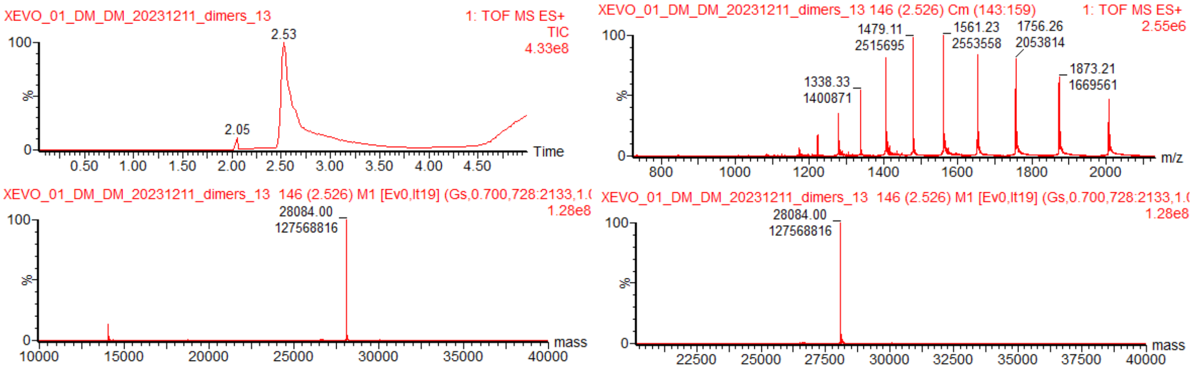

Calculated mass of GbD12 homoDiGb: 28084, Observed mass: 28084

GbMBP homoDiGb

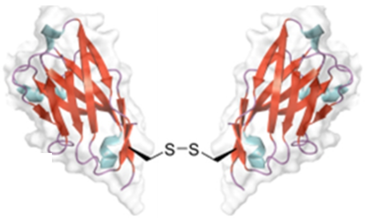

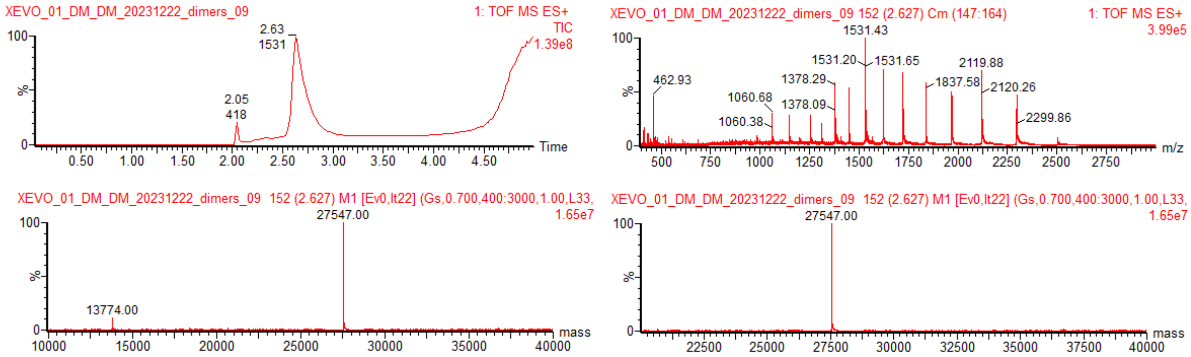

Calculated mass of GbMBP homoDiGb: 27546, Observed mass: 27547

GbRBD-1 homoDiGb

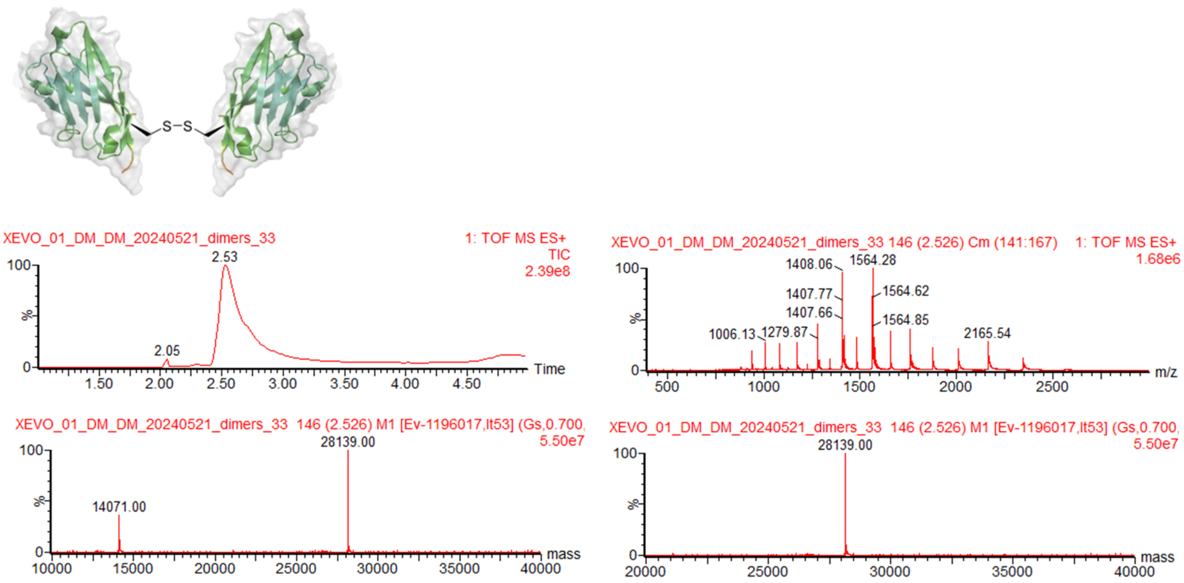

Calculated mass of GbRBD-1 homoDiGb: 28140, Observed mass: 28139

GbRBD3 homoDiGb

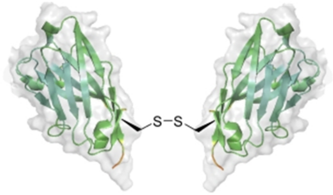

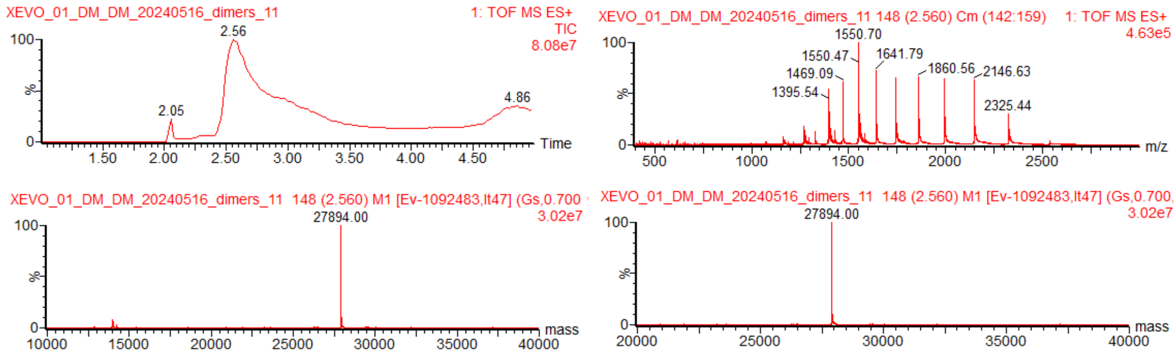

Calculated mass of GbRBD-3 homoDiGb: 27894, Observed mass: 27894

GbRBD-6 homoDiGb

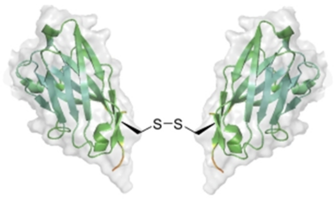

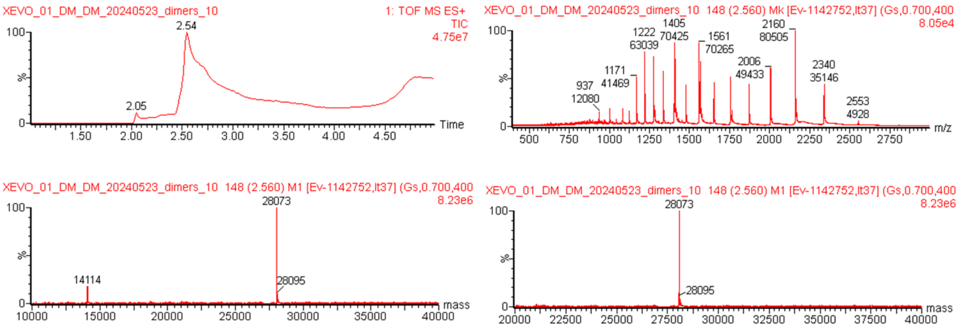

Calculated mass of GbRBD-6 homoDiGb: 28072, Observed mass: 28073

GbLys homoDiGb

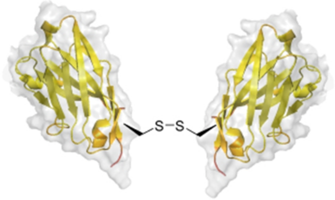

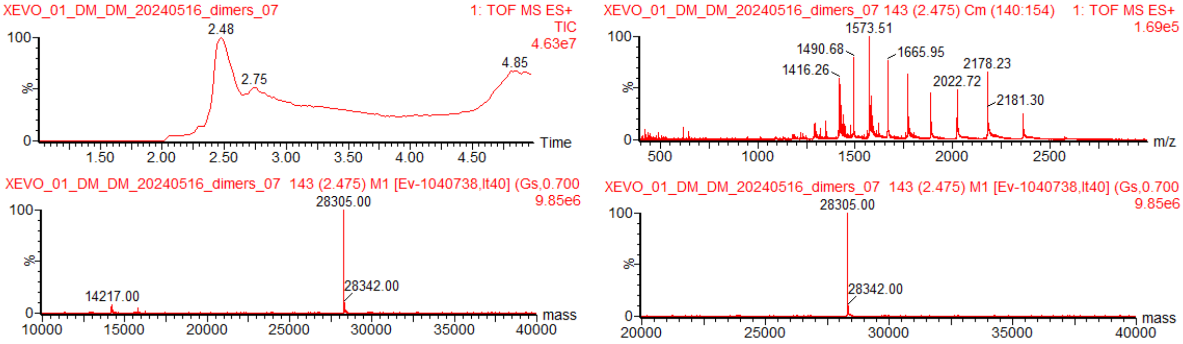

Calculated mass of GbLys homoDiGb: 28306, Observed mass: 28305

GbHIV homoDiGb

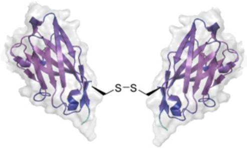

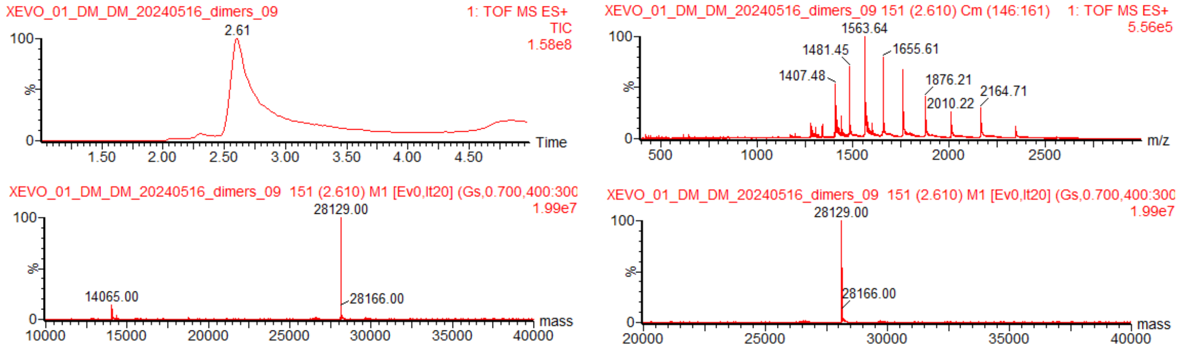

Calculated mass of GbHIV homoDiGb: 28130, Observed mass: 28129

General protocol for oxidative relay functionalization to generate heteroDiGbs
Gb5-006-TNB

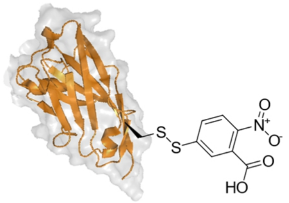

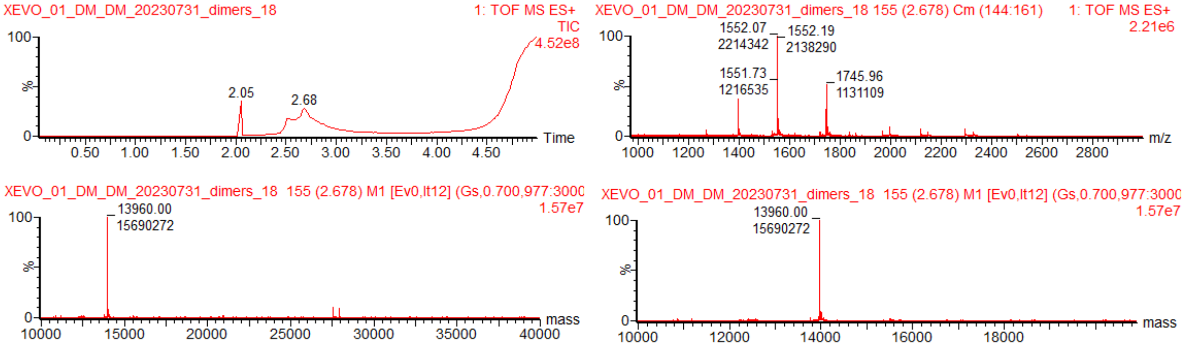

Calculated mass of Gb5-006-TNB: 13958, Observed mass: 13960

GbEnhancer-TNB

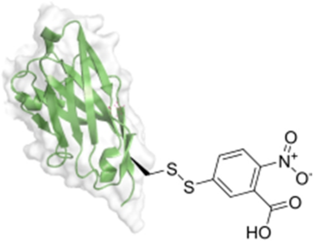

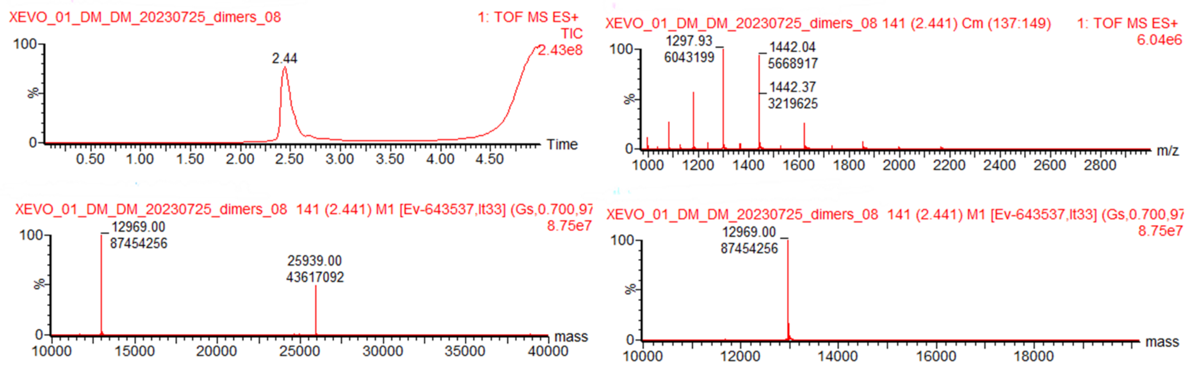

Calculated mass of GbEnhancer-TNB: 12958, Observed mass: 12969

GbH12-TNB

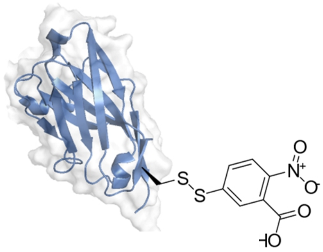

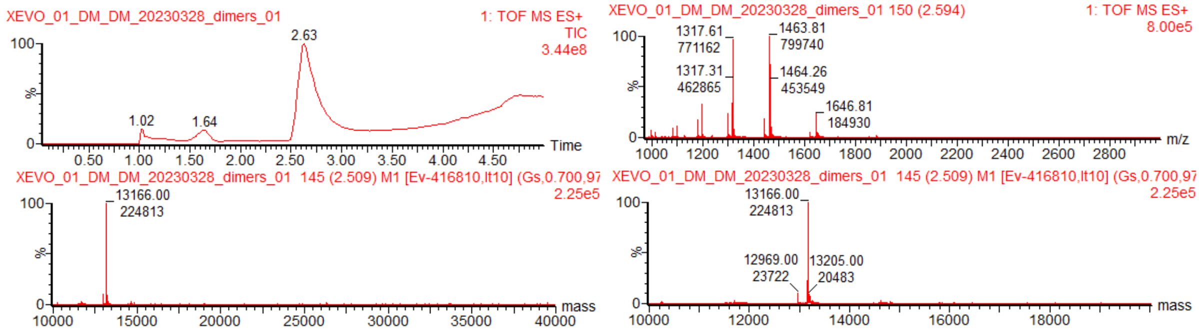

Calculated mass of GbH12-TNB: 13166, Observed mass: 13166

HeteroDiGb Gb5-006:GbEnhancer

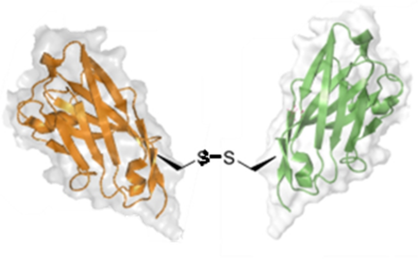

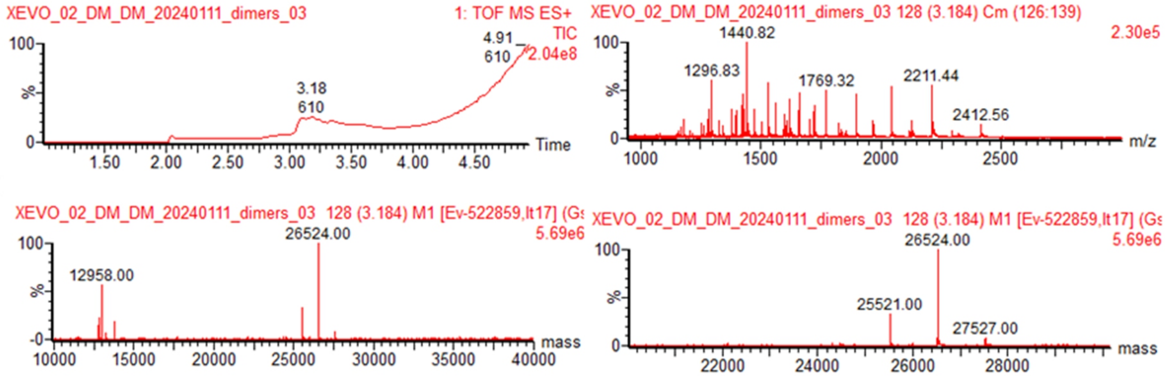

Calculated mass of HeteroDiGb Gb5-006:GbEnhancer: 26522, Observed mass: 26524. Peak with mass 25521 corresponds to GbEnhancer homoDiGb

HeteroDiGb (anti-SPNS2) GbC4:GbEnhancer

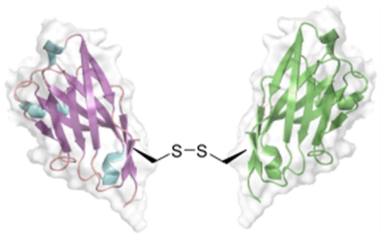

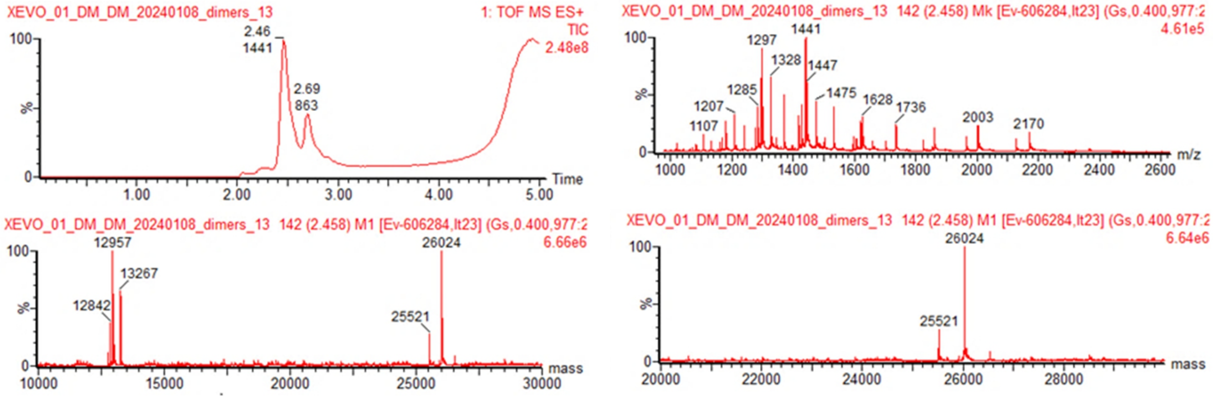

Calculated mass of HeteroDiGb GbEnhancer(anti-SPNS2) GbC4: 26026, Observed mass: 26024. Peak with mass 25521 corresponds to GbEnhancer homoDiGb

HeteroDiGb GbEnhancer:GbH12

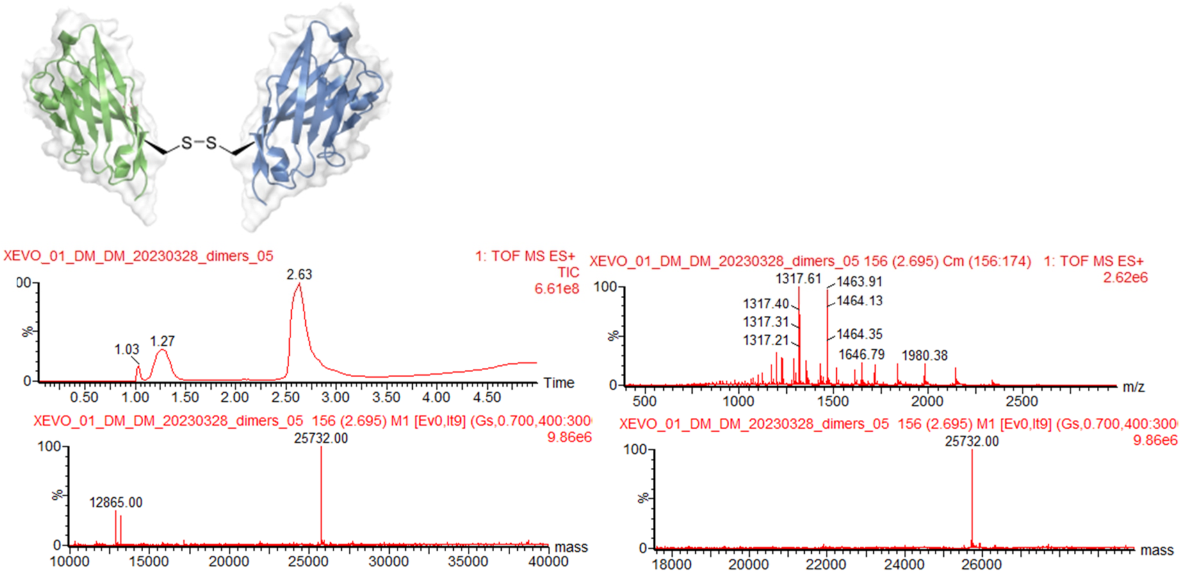

Calculated mass of HeteroDiGb GbEnhancer:GbH12: 25728, Observed mass: 25732

HeteroDiGb GbEnhancer:GbRBD6

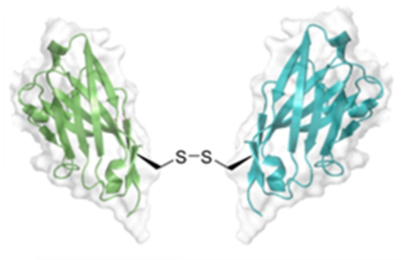

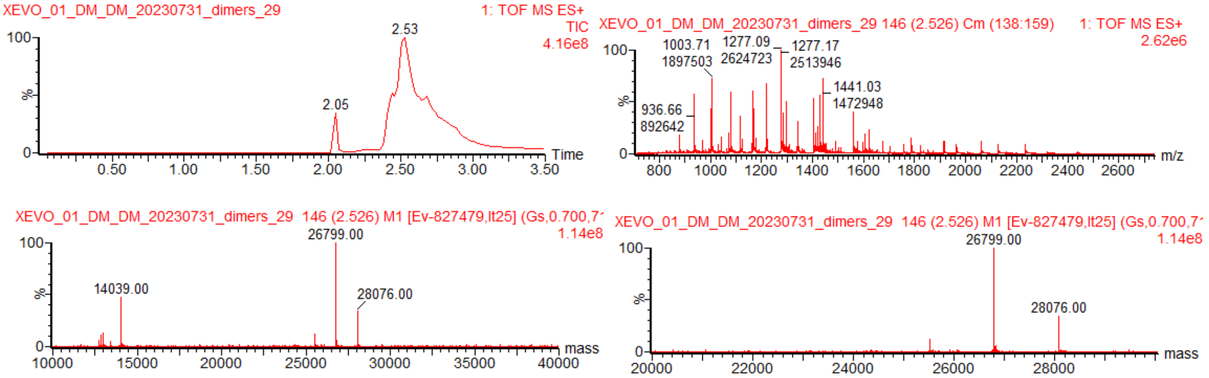

Calculated mass of HeteroDiGb GbEnhancer:GbRBD6: 26796, Observed mass: 26799. Peak with mass 28076 corresponds to anti-RBD6 homodimer

HeteroDiGb GbEnhancer:GbRBD1

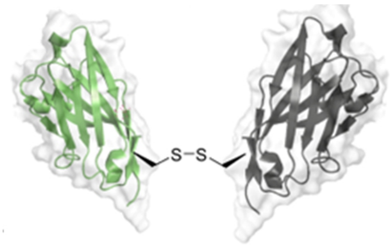

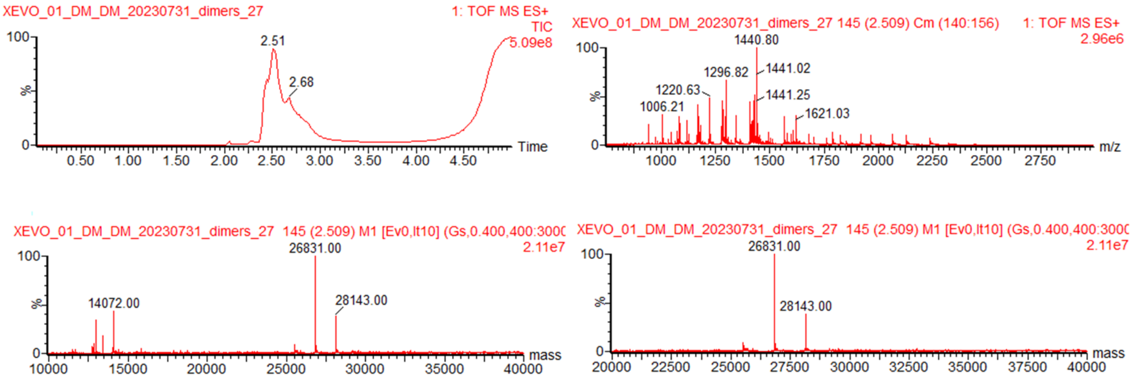

Calculated mass of HeteroDiGb GbEnhancer:GbRBD1: 26830, Observed mass: 26831. Peak with mass 25521 corresponds to GbEnhancer homoDiGb
