# Supplementary material for: Covalently constrained ‘Di-Gembodies’ enable parallel structure solutions by cryo-EM
Source: Nat Chem Biol. 2025 Aug 15;22(1):69–76. doi: 10.1038/s41589-025-01972-7 (PMC12435805; doi:10.1038/s41589-025-01972-7)
Supplement: Supplementary file 2 — Reporting Summary [file 41589_2025_1972_MOESM2_ESM.pdf]

nature portfolio

Corresponding author(s): Mingda Ye, Frank von Delft, David B. Sauer, Benjamin G. Davis, Robert J.C. Gilbert

Last updated by author(s): Jun 4, 2025

Reporting Summary

Nature Portfolio wishes to improve the reproducibility of the work that we publish. This form provides structure for consistency and transparency in reporting. For further information on Nature Portfolio policies, see our Editorial Policies and the Editorial Policy Checklist.

Statistics

For all statistical analyses, confirm that the following items are present in the figure legend, table legend, main text, or Methods section.

n/a	Confirmed	
☐	☒	The exact sample size (n) for each experimental group/condition, given as a discrete number and unit of measurement
☐	☒	A statement on whether measurements were taken from distinct samples or whether the same sample was measured repeatedly
☒	☐	The statistical test(s) used AND whether they are one- or two-sided<br>*Only common tests should be described solely by name; describe more complex techniques in the Methods section.*
☒	☐	A description of all covariates tested
☒	☐	A description of any assumptions or corrections, such as tests of normality and adjustment for multiple comparisons
☒	☐	A full description of the statistical parameters including central tendency (e.g. means) or other basic estimates (e.g. regression coefficient) AND variation (e.g. standard deviation) or associated estimates of uncertainty (e.g. confidence intervals)
☒	☐	For null hypothesis testing, the test statistic (e.g. F, t, r) with confidence intervals, effect sizes, degrees of freedom and P value noted<br>*Give P values as exact values whenever suitable.*
☒	☐	For Bayesian analysis, information on the choice of priors and Markov chain Monte Carlo settings
☒	☐	For hierarchical and complex designs, identification of the appropriate level for tests and full reporting of outcomes
☒	☐	Estimates of effect sizes (e.g. Cohen's d, Pearson's r), indicating how they were calculated

Our web collection on statistics for biologists contains articles on many of the points above.

Software and code

Policy information about availability of computer code

Data collection	Cryo-EM data were collected with EPU (version 2.13; Thermo Fisher Scientific).
Data analysis	Cryo-EM data were processed and analyzed with cryoSPARC (version 3.3.1; Structura Biotechnology), including the DeepEnhancer package (Version: 0.14). Additional software from the Collaborative Computational Projects No. 4 (CCP4; v7.0) and Electron cryo-Microscopy (CCPEM; v1.6.0) software suites were also used. Structural models were built using Coot (v0.8.9.5) with model refinement performed by PHENIX (v1.20).<br><br>Molecular visualisation, analysis and figure generation were performed using both PyMOL v_2.2.0 and v_3 (Schrodiner), and ChimeraX 1.16 (UCSF Resource for Biocomputing, Visualization, and Informatics).

For manuscripts utilizing custom algorithms or software that are central to the research but not yet described in published literature, software must be made available to editors and reviewers. We strongly encourage code deposition in a community repository (e.g. GitHub). See the Nature Portfolio guidelines for submitting code & software for further information.

Data

Policy information about availability of data

All manuscripts must include a data availability statement. This statement should provide the following information, where applicable:

- Accession codes, unique identifiers, or web links for publicly available datasets
- A description of any restrictions on data availability
- For clinical datasets or third party data, please ensure that the statement adheres to our policy

Cryo-EM density maps have been deposited in the EMDB under accession codes EMD-19331, EMD-19332, EMD-19333, EMD-19334, EMD-19335, EMD-19336, EMD-19337, EMD-19338, EMD-19339, EMD19340,EMD50430,EMD50432,EMD50433,EMD50525 and corresponding coordinate files have been deposited in PDB under accession codes 8RL5, 8RL6, 8RL7, 8RL8, 8RL9, 8RLA, 8RLB, 8RLC, 8RLD, 8RLE, 9FGV, 9FGY, 9FGZ, 9FKQ. The map and model IDs are detailed in Extended Data Table 1. Other datasets used other PDB:7ZMV 3K1K 6JB2 5M13 and 8QV6. All data will be made available on publicly accessible dataset.

Human research participants

Policy information about studies involving human research participants and Sex and Gender in Research.

Reporting on sex and gender	*Use the terms sex (biological attribute) and gender (shaped by social and cultural circumstances) carefully in order to avoid confusing both terms. Indicate if findings apply to only one sex or gender; describe whether sex and gender were considered in study design whether sex and/or gender was determined based on self-reporting or assigned and methods used. Provide in the source data disaggregated sex and gender data where this information has been collected, and consent has been obtained for sharing of individual-level data; provide overall numbers in this Reporting Summary. Please state if this information has not been collected. Report sex- and gender-based analyses where performed, justify reasons for lack of sex- and gender-based analysis.*
Population characteristics	*Describe the covariate-relevant population characteristics of the human research participants (e.g. age, genotypic information, past and current diagnosis and treatment categories). If you filled out the behavioural & social sciences study design questions and have nothing to add here, write "See above."*
Recruitment	*Describe how participants were recruited. Outline any potential self-selection bias or other biases that may be present and how these are likely to impact results.*
Ethics oversight	*Identify the organization(s) that approved the study protocol.*

Note that full information on the approval of the study protocol must also be provided in the manuscript.

Field-specific reporting

Please select the one below that is the best fit for your research. If you are not sure, read the appropriate sections before making your selection.

☒ Life sciences ☐ Behavioural & social sciences ☐ Ecological, evolutionary & environmental sciences

For a reference copy of the document with all sections, see nature.com/documents/nr-reporting-summary-flat.pdf

Life sciences study design

All studies must disclose on these points even when the disclosure is negative.

Sample size	For example, in the context of EM data collection, we acquired approximately 10,000 images across all samples for data processing. We consider this number sufficient for our analysis.
Data exclusions	None
Replication	Cryo-EM data collection was only carried out on a single sample in each case.<br>In vitro binding assays were carried out with 3 biological replicates.
Randomization	Randomization is considered in our studies. For example. Even-odd separation to two half datasets. This is standard and reckoned to be the best way to separate image data randomly.
Blinding	Blinding was not used. It is not relevant as outcomes of the experiments are not affected by knowledge of the sample.

Reporting for specific materials, systems and methods

We require information from authors about some types of materials, experimental systems and methods used in many studies. Here, indicate whether each material, system or method listed is relevant to your study. If you are not sure if a list item applies to your research, read the appropriate section before selecting a response.

Materials & experimental systems

n/a	Involved in the study
☐	☒ Antibodies
☐	☒ Eukaryotic cell lines
☒	☐ Palaeontology and archaeology
☒	☐ Animals and other organisms
☒	☐ Clinical data
☒	☐ Dual use research of concern

Methods

n/a	Involved in the study
☒	☐ ChIP-seq
☒	☐ Flow cytometry
☒	☐ MRI-based neuroimaging

Antibodies

Antibodies used	Gb5-006,GbD12,GbS2A4,GbEnhancer,GbH12,GbLysozyme,GbHIV.GbRBD3,GbMBP,GbC4,GbRBD1,GbRBD6,NbRECQL5
Validation	Nanobodies/Gluebodies were validated by biolayer interferometry, mass spectrometry, and structural studies, as described in the manuscript.

Eukaryotic cell lines

Policy information about cell lines and Sex and Gender in Research

Cell line source(s)	Spodoptera frugiperda (Sf9) cells (Thermo-Fisher Scientific, Cat. No. 11496015). Expi293F GnTI- cells (Cat# A39240; Thermo Fisher Scientific). HEK293T cells: HEK-293T (CRL-11268, ATCC(USA))
Authentication	Cell lines used (SF9/Expi293F/HEK293T) are standard laboratory model overexpression strains purchased from Thermo Fisher. These cell lines undergo quality control before dispatch. Cells were passaged a limited number of times before a new batch from the manufacturer was employed. Cells were monitored by regular visual inspection. These cell lines have not been authenticated
Mycoplasma contamination	The cell lines were not tested for mycoplasma contamination
Commonly misidentified lines (See ICLAC register)	No common misidentified cell lines were used in the study
